# Post-marketing withdrawal of 462 medicinal products because of adverse drug reactions: a systematic review of the world literature

**DOI:** 10.1186/s12916-016-0553-2

**Published:** 2016-02-04

**Authors:** Igho J. Onakpoya, Carl J. Heneghan, Jeffrey K. Aronson

**Affiliations:** Centre for Evidence-based Medicine, Nuffield Department of Primary Care Health Sciences, University of Oxford, New Radcliffe House, Radcliffe Observatory Quarter, Oxford, OX2 6GG UK

**Keywords:** Adverse drug reaction, Drug withdrawal, Systematic review, Voluntary recall

## Abstract

**Background:**

There have been no studies of the patterns of post-marketing withdrawals of medicinal products to which adverse reactions have been attributed. We identified medicinal products that were withdrawn because of adverse drug reactions, examined the evidence to support such withdrawals, and explored the pattern of withdrawals across countries.

**Methods:**

We searched PubMed, Google Scholar, the WHO’s database of drugs, the websites of drug regulatory authorities, and textbooks. We included medicinal products withdrawn between 1950 and 2014 and assessed the levels of evidence used in making withdrawal decisions using the criteria of the Oxford Centre for Evidence Based Medicine.

**Results:**

We identified 462 medicinal products that were withdrawn from the market between 1953 and 2013, the most common reason being hepatotoxicity. The supporting evidence in 72 % of cases consisted of anecdotal reports. Only 43 (9.34 %) drugs were withdrawn worldwide and 179 (39 %) were withdrawn in one country only. Withdrawal was significantly less likely in Africa than in other continents (Europe, the Americas, Asia, and Australasia and Oceania). The median interval between the first reported adverse reaction and the year of first withdrawal was 6 years (IQR, 1–15) and the interval did not consistently shorten over time.

**Conclusion:**

There are discrepancies in the patterns of withdrawal of medicinal products from the market when adverse reactions are suspected, and withdrawals are inconsistent across countries. Greater co-ordination among drug regulatory authorities and increased transparency in reporting suspected adverse drug reactions would help improve current decision-making processes.

**Electronic supplementary material:**

The online version of this article (doi:10.1186/s12916-016-0553-2) contains supplementary material, which is available to authorized users.

## Background

Drug regulatory authorities award marketing authorizations that license pharmaceutical companies to market medicinal products when there is sufficient evidence that the product has a favourable benefit-to-harm balance [1]. If a new adverse drug reaction is suspected after approval, several courses of action can be taken by the regulator and/or manufacturer, including adding a new product label with specific warnings [2], adding a new contraindication [3], issuing a Direct Healthcare Professional Communication [4], allowing patients to decide whether they will take the drug [5], and in the most serious cases, withdrawal or revocation of the licence [6].

Post-approval withdrawal of medicinal products because of adverse drug reactions can be triggered by evidence obtained from various sources – anecdotal reports, observational studies, clinical trials, systematic reviews, or animal data. The removal of previously approved products from the market can result in loss of confidence in medicines by the public, loss of effective compounds (i.e. effective for treating the specific indication, but for which the benefit-to-harm balance was considered unfavourable), and loss of revenue for drug manufacturers. When there is no concrete evidence linking drug use with a suspected adverse reaction, such withdrawals can be contentious.

We have previously reported inconsistencies in the pattern of withdrawals of 95 medicines to which deaths were attributed [7]. To date, there has been no comprehensive and systematic review of medicinal products that have been withdrawn because of adverse drug reactions in general. In addition, there is a paucity of data about the evidence on which such withdrawal decisions are based. Furthermore, the pattern of post-approval withdrawals across geographical regions worldwide has never been examined. Therefore, we performed a systematic review to identify medicinal products that have been withdrawn after approval because of any kinds of adverse drug reactions; to assess the types of evidence on which the withdrawal decisions were based; to identify the types of attributed adverse drug reactions responsible; to examine the patterns of withdrawal across geographical regions; to examine the intervals between launch dates and (1) the times of the first reports of adverse reactions and (2) the first dates of withdrawal; and to examine the intervals between the first reports of adverse drug reactions and the first withdrawals.

## Methods

### Search strategy

We searched for medicinal products withdrawn from the market because of adverse drug reactions between 1950 and December 2014 from the following sources:

- the World Health Organization’s (WHO’s) database of Consolidated List of Products whose consumption and/or sale have been banned, withdrawn, severely restricted, or not approved by governments (Issues 6, 8, 12, and 14, and the updated version of issue 14)
- the WHO’s Drug Information (Volumes 1–28)
- the WHO’s Pharmaceuticals Newsletter (1997–2014)
- Meyler’s Side Effects of Drugs: The International Encyclopaedia of Adverse Drug Reactions and Interactions, volumes 1–8 and editions 9–15, and the Side Effects of Drugs Annuals 1–36
- Stephens’ Detection of New Adverse Drug Reactions, 5th edition [8]
- the Pharmaceutical Manufacturing Encyclopedia, 3rd edition [9]
- The Merck Index, 15th Edition [10]
- the website of the UK Medicines and Healthcare products Regulatory Agency
- the website of the US Food and Drug Administration (FDA)
- the database of withdrawn drugs of the European Medicines Agency
- Health Canada Drug Product Database
- the website of the Indian Central Drugs Standard Control Organization
- the website of the Australian Therapeutic Goods Administration
- the website of the Nigeria National Agency for Food and Drug Administration and Control
- the website of the Ghana Food and Drugs Authority
- the website of the South Africa Medicines Control Council
- PubMed
- Medline
- Google Scholar

Please see Additional file 1: Web 1 for full list of drug regulatory websites assessed.

For each medicinal product withdrawn, we then searched PubMed, Medline, and Google Scholar for the first reported adverse drug reaction.

Search terms used included “drug withdrawal”, “fatal*”, “death(s)”, “side effect”, “adverse effect”, “adverse reaction”, “adverse event”, “poison”, “toxicity”, “voluntary recall”, “suspension”, “prohibition”, “banned”, “remov*”, “revoke*”, “discontinued” (a Medline search strategy is included as a Additional file 2: Web 2). If we could not find information for a medicinal product using its chemical name for searches, we used the trade name or code name. We also searched the references of retrieved full texts for any earlier dates of reports of suspected adverse reactions. If an article had evidence of an earlier reported date, that date was chosen as the first adverse reaction date. If a drug was withdrawn because of two or more adverse reactions, we used the first reported date of any such reactions.

To determine the accuracy of launch dates, we compared the information on the WHO database of Consolidated List of Products with the information in the Merck index, the Pharmaceutical Manufacturing Manual, and a newly developed database for withdrawn and discontinued products [11]. To determine the dates of first withdrawal and countries of withdrawal, we cross-checked WHO data with the database for withdrawn products; if the information was not found in that database, we compared the dates with the results of searches in PubMed and Google Scholar.

### Inclusion/exclusion criteria

We define a medicinal product as “*Any substance or combination of substances which may be used in, or administered to, human beings, either with a view to restoring, correcting or modifying physiological functions by exerting a pharmacological, immunological or metabolic action, or to making a medical diagnosis*” [12]. For the purpose of our review, such applications could be via oral, intravenous, intramuscular, sublingual, inhalational, rectal, or topical routes. To be included in the review, a product must have been withdrawn from the market because of reports of a suspected adverse reaction or reactions, or problems related to hazards or harms. We included medicinal products that had previously been withdrawn (by regulatory authorities and/or drug manufacturers) because of adverse reactions but had been re-introduced or made available in other, safer, formulations. When only one formulation of a product was withdrawn, the product was included in the list, and the formulation was noted; however, if all formulations of the product were subsequently withdrawn, we used the earliest date, irrespective of formulation, as the year of first withdrawal. We did not exclude products based on routes of administration. We excluded medicines for which there was documented regulatory evidence that they had been voluntarily withdrawn by marketing authorization holders solely for commercial reasons, or withdrawn based on contamination of the active ingredient by other agents (such as organisms and active or toxic compounds). We also excluded herbal products, non-human medicines, and non-prescription medicines.

### Assessing the types of evidence

We documented the highest level of available evidence before the year of first withdrawal of products, based on the Oxford Centre for Evidence-based Medicine (OCEBM) criteria [13] rating the levels of evidence of harms as follows: Level 5, mechanism-based reasoning (lowest); Level 4, case-series or case-control studies; Level 3, non-randomized, cohort, or follow-up studies; Level 2, randomized clinical trials; and Level 1, systematic reviews (highest). One reviewer (IJO) documented the levels of evidence, which were independently verified by a second reviewer (JKA). Discrepancies were resolved through discussion.

### Data extraction

For each withdrawn product, we extracted data on the date of marketing authorization, the launch date, or the date of first recorded use; the drug class and therapeutic indication [14]; the year in which an adverse drug reaction related to the reason for withdrawal was first reported; the year of first withdrawal; the country or countries of withdrawal; and the reported organ or system that was affected by the drug. When the exact launch date of a product was not available (16 cases), we used the first date of reported use in humans by cross-checking references on PubMed with Medline. If two or more adverse reactions were reported as reasons for withdrawal, we used the date of the first reported reaction.

One reviewer (IJO) extracted the data and a second reviewer (JKA) verified them independently. When there were discrepancies in the attributed dates, the reviewers re-checked the dates together and arrived at a consensus by discussion.

### Statistical analyses

We used summary tables to document the intervals between launch year and the year of first reports of adverse drug reactions, the interval between launch year and the year of first withdrawal, and the interval between the first report of an adverse drug reaction and the year of first withdrawal. Because these intervals were skewed, we used medians and interquartile ranges (IQR) as measures of central dispersion.

We used scatter plots to explore the relationships between launch dates and times to first reports of adverse drug reactions and withdrawals.

Because drug regulatory systems in most African countries are not well developed [15–17], we compared withdrawal rates in Africa with five other continents. We computed the relative rates (RR) and 95 % confidence intervals (CI) of withdrawals per country in Africa versus the other five continents. A *P* value <0.05 was considered statistically significant.

## Results

**Fig. 1:**
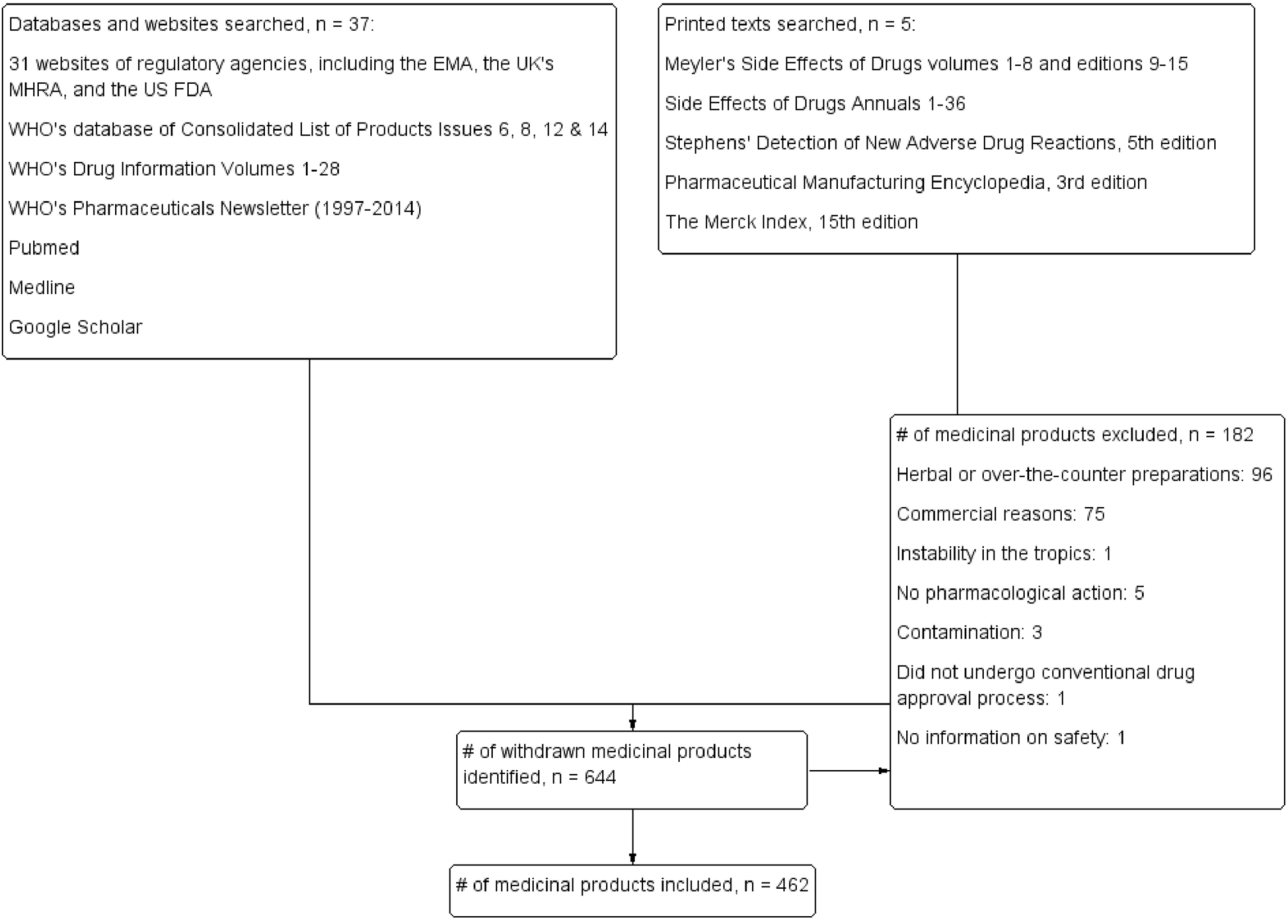
Schematic diagram showing process for inclusion of medicinal products withdrawn after approval because of adverse drug reactions

We identified 644 withdrawn medicinal products (Fig. 1), of which 96 were excluded because they were marketed as herbal or over-the-counter preparations, another 75 because they were withdrawn for commercial reasons, one (ergometrine) because of instability in tropical conditions, five because they had no pharmacological actions (e.g. colourings and artificial sweeteners), three because the reason for withdrawal was contamination, one because there was no information on adverse reactions, and one because it was not licensed through conventional drug approval procedures, leaving 462 products. The withdrawals occurred between 1953 and 2013 (except for dinitrophenol which was first withdrawn in 1938 in the USA, and prohibited for use in humans again by the FDA in 1986). The details of the withdrawn drugs are available in Additional file 3: Table S1.

### Levels of evidence used for drug withdrawals

The levels of available evidence that triggered drug withdrawal decisions, according to the OCEBM criteria, are shown in Table 1. Of the 462 included products, case reports were used as evidence for withdrawals in 330 instances (71 %); in 49 cases (11 %), withdrawal decisions were based on the results of animal studies. Of products launched after 1950 (n = 354), case reports were used as evidence in 247 instances (70 %). The comparable figures for each decade since 1950 are as follows: 1950s 85 % (58/68 products); 1960s 74 % (65/88); 1970s 69 % (54/77); 1980s 68 % (34/50); 1990s 64 % (27/45); 2000–2008, 35 % (9/26).

**Table 1:** Levels of evidence used to justify post-marketing withdrawal of medicinal products

Level of evidence^a^	Number (%) of withdrawals	
	All marketed drugs (n = 462)	Marketed drugs launched since 1950 (n = 286)
Level 1: Systematic reviews	6 (1.3)	6 (2.1)
Level 2: Randomized studies	27 (5.8)	25 (8.7)
Level 3: Non-randomized studies	43 (9.3)	30 (10.5)
Level 4: Case reports	330 (71.4)	189 (66.1)
Level 5: Mechanism-based reasoning	56 (12.1)	36 (12.6)

^a^Based on the Oxford Centre for Evidence-Based Medicine Levels of Evidence [13]. Level 1, Systematic review of randomized trials, systematic review of nested case-control studies; Level 2, Individual randomized trial or (exceptionally) observational study with dramatic effect; Level 3, Non-randomized controlled cohort/follow-up study (post-marketing surveillance); Level 4, Case-series, case-control, or historically controlled studies; Level 5, Mechanism-based reasoning

### Types of adverse drug reactions

Hepatotoxicity (81 cases; 18 %) was the most commonly reported adverse drug reaction that led to withdrawal (Additional file 3: Table S1), followed by immune-related reactions (79 cases; 17 %), cardiotoxicity (63 cases; 14 %), neurotoxicity (76 cases; 16 %), haematological toxicity (53 cases; 11 %), carcinogenicity (61 cases; 13 %), and drug abuse and dependence (52 cases; 11 %). Deaths were associated with withdrawals in 114 cases (25 %).

### Patterns of withdrawals

Of the 462 products, 43 (9.3 %) were withdrawn worldwide and 179 (39 %) were withdrawn in only one country; the remaining 240 (52 %) were withdrawn in two or more countries. In terms of withdrawals by geographical region, 63 products were withdrawn in Africa, 150 in Asia, 32 in Australasia and Oceania, 309 in Europe, 134 in North America, and 65 in South America (Table 1). The rate of withdrawals per country was significantly lower in Africa than in Asia, Australasia, Europe, North America, or South America (Table 2). However, there were no significant differences in the relative rates of withdrawals across the five African sub-regions (data not shown).

**Table 2:** Post-marketing withdrawal of medicinal products because of adverse drug reactions in different continents

Continent	No. of countries	Total population (millions)	No. of withdrawn products	Rate of withdrawals/million population	Rates of withdrawal/country	RR of withdrawal per country versus Africa (95 % CI)^a^	^a^ *P* value versus Africa
Africa	54	1111	63	0.06	1.17	–	–
Asia	46	4427	150	0.03	3.26	1.42 (1.18–1.71)	0.001
Australasia & Oceania	11	30	32	1.07	2.91	1.38 (1.08–1.76)	0.045
Europe	50	742.5	309	0.42	6.18	1.60 (1.34–1.90)	<0.0005
N. America	23	528.7	134	0.25	5.83	1.59 ( 1.32–1.90)	<0.0005
S. America	12	387.5	65	0.17	5.42	1.57 (1.29–1.90)	<0.0005

^a^The *P* values have been corrected for multiple tests using the Bonferroni method. The relative rates of withdrawal are calculated based on the assumption that if a medicinal product was withdrawn in one country, it should also have been withdrawn in all countries in that continent. The data for total populations are obtained from the 2013 World Population Data Sheet (http://www.prb.org/pdf13/2013-WPDS-infographic_MED.pdf). This analysis excludes 43 medicinal products withdrawn worldwide

### Interval between launch year and first reported adverse drug reaction

The median interval between launch year and the year in which an adverse reaction was first reported was 8 years (IQR, 2–20) for all the drugs and 4 years (IQR, 1–10) for drugs launched after 1960.

**Fig. 2:**
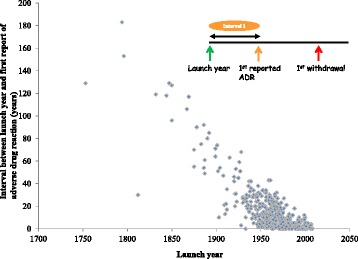
Launch year versus interval 1 (time lapse between launch year and first reported adverse drug reaction)

The more recent the launch date of a drug, the quicker a report of an adverse reaction appeared in the literature (Fig. 2). A similar trend was observed for drugs launched after 1960.

### Interval between launch year and first withdrawal

**Fig. 3:**
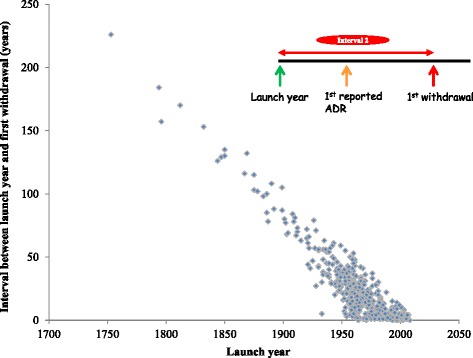
Launch year versus interval 2 (time lapse between launch year and date of the first withdrawal)

The median interval between first launch and first withdrawal was 18 years (IQR, 6–34) for all the drugs and 10 years for drugs introduced after 1960 (IQR, 3–19). There were trends towards shorter delays between the year of first launch and the year of first withdrawal for all 462 drugs and for the 286 products launched after 1960 (Fig. 3).

### Interval between the first reported adverse drug reaction and the first withdrawal

**Fig. 4:**
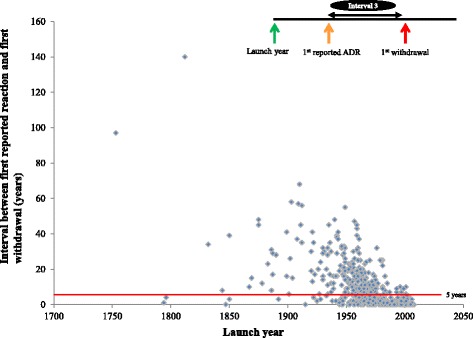
Launch year versus interval 3 (time lapse between the first reported adverse drug reaction and the date of first withdrawal from launch year)

The median interval between the first reported adverse reaction and the year of first withdrawal was 6 years (IQR, 1–15) for all drugs and 3 years for drugs launched after 1960 (IQR, 0–8). Figure 4 shows that there was a trend towards a shorter interval between the first reported adverse reaction and the first withdrawal. However, for drugs launched after 1960, there was no consistently shorter trend. Similar results were observed when we examined the delays to withdrawal after reports of adverse drug reactions in each of the six continents separately (data not shown).

### Relation between time of launch to first adverse drug reaction report versus the interval between the first adverse drug reaction report and the first withdrawal

**Fig. 5:**
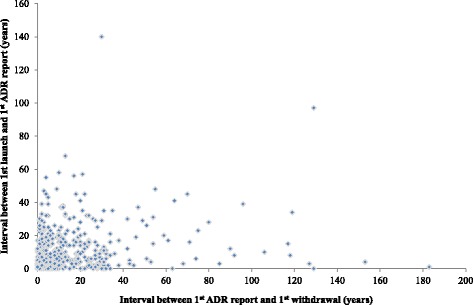
Interval between first launch and first ADR report (Interval 1) versus time to withdrawal after first ADR report (Interval 3)

Figure 5 shows that quicker reports of adverse drug reactions were not associated with a corresponding shortening of the time to regulatory action following such reports. This finding was also observed for medicinal products launched after 1960.

## Discussion

We have identified 462 medicinal products withdrawn because of adverse drug reactions between 1953 and 2013. Hepatotoxicity and immune-mediated reactions were responsible for over 30 % of withdrawals, and death was attributed as among the reasons for withdrawal in 25 % of instances. Withdrawals were significantly less common in Africa than in Asia, Europe, and North and South America.

### Evidence for withdrawal

Case reports were most often used as evidence on which withdrawal decisions were based, being used in 71 % of all the products, 70 % of those launched after 1950, and 66 % of those launched after 1960. This corroborates our previous finding that case reports were most commonly used as evidence for withdrawal of 95 medicinal products because of drug-attributed deaths [7], and confirms that formal studies are often not conducted when adverse drug reactions are reported anecdotally [18]. However, the frequency with which anecdotal reports have provided the dominant source of information has reduced with time, from 85 % in the 1950s to 64 % in the 1990s; since 2000, the frequency has fallen even further, to 35 %, but the number of products affected during the last few years is relatively small.

### Patterns of withdrawal

There were significantly fewer withdrawals in Africa than in the other five continents. This suggests that there is better co-ordination among drug regulatory authorities in those geographical regions than in Africa. Furthermore, the delays between the first report of an adverse reaction and first withdrawal were more often than not longer in African countries than in Europe or North America, which were not significantly different from each other. Thus, harmful drugs are likely to stay on the market for longer in Africa.

There was a lower rate of withdrawal in African countries than elsewhere. Factors that can affect withdrawals include the strength of a local regulatory agency, and the availability of proper monitoring facilities or preventive strategies. According to the WHO, only 4 % of African nations have moderately developed pharmacovigilance systems and 39 % lack adequate regulatory capacity [19]. Furthermore, the ability of a country to restrict access to harmful drugs is related to per capita gross national product [20], and this contributes to the so-called medical poverty trap (increases in overall out-of-pocket expenses for health care in families who are already poor) [21].

### Delays between launch dates and reports of adverse drug reactions

The interval between first launch and first report of adverse reactions has shortened over time (Fig. 2). This is probably largely due to improved pharmacovigilance, better methods of signal detection, and better reporting of suspected adverse drug reactions. However, at least 5 years elapsed before the first report of an adverse drug reaction in 31 % of instances for products launched after 1960, suggesting that detection of adverse reactions to approved medicinal products has improved with developments in drug regulation, but the improvements have not been substantial. This may be attributable to a variety of factors, such as selective reporting of benefits and harms in clinical trials [22] and flawed regulatory assessment procedures [23], which have led to calls for changes to current drug adverse event monitoring strategies [24, 25].

Regulatory assessment of clinical trials data of medicinal products is seldom conducted in many African countries, where the drug regulatory processes are geared to grant marketing licences for imported products that have been assessed in other places and populations.

### Under-reporting of adverse drug reactions

Under-reporting of adverse drug reactions could cause delays in making withdrawal decisions. There is evidence that clinicians selectively report adverse drug reactions [26, 27], and the authors of a review of hospital admissions due to adverse drug reactions concluded that physicians seldom report such events when they occur [28]. A low rate of reporting among health-care professionals could be due to poor knowledge of how to use the spontaneous reporting systems [29], conflicts of interest [30], forgetfulness, lack of time, and uncertainty about causal relationships between drugs and adverse events [31]. Proactive measures to encourage physicians to report suspected adverse drug reactions have been suggested [32]. Indeed, provision of economic incentives and/or educational activities improves the reporting of adverse drug reactions among hospital clinicians [33–37]. Patients are also likely to under-report suspected adverse reactions to medications [38], and empowerment of patients has been advocated [39, 40].

### Delays to withdrawals after reports of adverse drug reactions

**Fig. 6:**
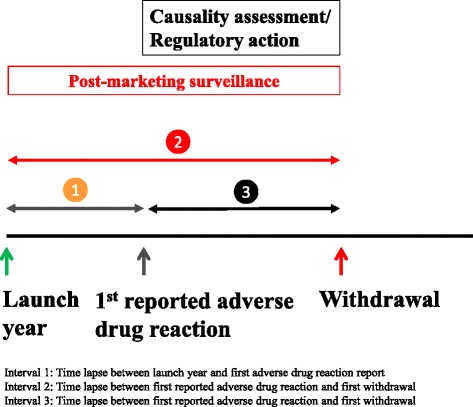
Schematic diagram of the intervals following the launch of a medicinal product. The shortening in Interval 2 is due to a shortening in Interval 1

There was no consistent reduction in the interval between the first report of an adverse drug reaction and the first withdrawal from the market (Fig. 5), suggesting that the shortened interval between the first launch and the first withdrawal was largely due to a shortening in the interval between the first launch and the first report of an adverse reaction. Therefore, difficulty in assessing causality and inconsistencies in how regulatory actions are enforced after reports of suspected adverse drug reactions could explain the observed delays and discrepancies (Fig. 6); this is corroborated by the absence of a relationship between the interval between first launch and first adverse drug reaction report (Interval 1) versus the interval between first adverse drug reaction report and first withdrawal (Interval 3; Fig. 6). We note that from 1985 onwards, over 80 % of withdrawals occurred within 5 years of initial adverse reaction reports compared with just over 50 % for the 462 products, suggesting that the delays to withdrawal following reports of adverse reactions has generally improved since the thalidomide incident of the 1960s. Difficulties in determining causality in part explain why drugs are withdrawn in one country but remain available in another. The need to develop a universally accepted algorithm for diagnosing adverse drug reactions has been highlighted [41, 42].

### Frequency of withdrawals compared with overall new drug approvals

The number of withdrawn medicinal products is probably only a small fraction of overall approvals. As examples, less than 2 % of new drug approvals by the FDA between 1950 and 2011 [43] and 3 % of approved products in Canada and the USA between 1992 and 2011 were withdrawn [44], suggesting that drug regulatory authorities have made considerable efforts at ensuring that harmful drugs are not marketed.

### Comparison with previous studies

We have confirmed and extended the result of previous studies, all of which have been considerably smaller and of more limited time spans. We have also documented the levels of evidence used for making the withdrawal decisions, analysed time courses, and included data from African countries, not previously reported.

For example, an analysis of 19 medicinal products withdrawn between 2002 and 2011 showed that case reports were commonly used to justify withdrawal decisions, but less so with time [45]. A review of 121 withdrawn products showed that hepatotoxicity, cardiotoxicity, and carcinogenicity were the most common reasons for withdrawals between 1960 and 1999 [46]. There were inconsistencies in the patterns of withdrawal of 26 products between 1971 and 1992 in the UK and the USA [47], and in drug withdrawal policies across countries [48], although an earlier study of 24 products suggested consistency in withdrawal patterns between the UK and USA from 1964 to 1983 [49]. In a study of 22 products withdrawn in Canada between 1990 and 2009 [44], the median interval between approval and withdrawal was 3.5 years (IQR, 1.9–7.9); our data, analysed over the same period, show a value of 3 years (IQR, 1–6; n = 72). Our results are consistent with all of these findings.

### Strengths and limitations

We used robust methods to search for medicinal products withdrawn because of adverse reactions and documented the evidence on which the withdrawal decisions were based. In addition, we accessed data from a variety of sources. However, we recognize some limitations. We do not have information on the delay between the actual occurrence of an adverse drug reaction due to a medicinal product and the date it first appeared in the literature. Nevertheless, such delays are unlikely to have significantly influenced the results. We do not have data from countries in Africa that are blighted by armed conflict, e.g. Burundi, the Central African Republic, and Somalia; indeed, we did not identify any information on withdrawn medicinal products from these countries in the WHO drug lists. Furthermore, as of 2009, over half of all African countries did not have a drug regulatory website [50]. We did not access data from non-English drug regulatory websites; however, we do not think that the information from such sites would significantly alter our results, since a majority of such countries report their data to the WHO. In addition, the accuracy of the information from the databases used to document launch and withdrawal dates has not been assessed, but we did use information from other selected texts to check for any inconsistencies. Furthermore, we do not have data on countries in which withdrawn drugs were approved by regulatory authorities, where such exist; this difficulty has previously been reported by other authors [47].

We may not have identified all medicinal products withdrawn in association with adverse drug reactions because of the possibility of negative publication bias, and we do not know how many patients in all were affected by adverse reactions. This could also have influenced the speed with which regulatory decisions were taken. Some medicines are available only on prescription in some regions and may be available over the counter elsewhere. For example, antibiotics are generally available as prescription-only medicines in the UK [51]; in contrast, the results of surveys in Nigeria showed that self-medication with antibiotics and antimalarial drugs was common both in the general public and among healthcare workers [52, 53].

### Recommendations

- Universal guidelines for determining when a drug should be withdrawn when serious adverse drug reactions are suspected should be developed and promoted.
- More efforts should be made towards strengthening drug monitoring systems in low- and middle-income economies, especially in Africa; the proposal by the WHO in collaboration with African Union countries to establish an African Medicines Agency by 2018 is a welcome development.
- Regulatory authorities and drug manufacturers should expedite action when adverse drug reactions are suspected; formal studies to test for such associations should be conducted sooner rather than later; temporary suspensions or restrictions could be considered.
- There should be more transparency in reporting adverse events observed during clinical trials; access to clinical study reports should be a priority for future drug regulation.
- More active engagement of health professionals and patients in reporting suspected adverse drug reactions should be encouraged.

## Conclusions

The interval between launch date and reports of adverse drug reactions has shortened over the past few decades, perhaps because of better reporting of suspected adverse reactions or stricter regulation. In addition, increasing numbers of individuals may have been exposed to the withdrawn products in recent years, leading to quicker detection of adverse reactions. However, withdrawal of products following reports of suspected adverse reactions, sufficiently serious to warrant withdrawal, has not improved consistently over the last 60 years. In addition, harmful drugs are less likely to be withdrawn in African countries. Greater co-ordination among drug regulatory authorities and increased transparency in the reporting of suspected adverse drug reactions would help improve decision-making processes.

## Additional files

Additional file 1:
**Web 1.** List of English Language drug regulatory websites searched for information on medicinal products withdrawn from the market because of adverse drug reactions. (PDF 299 kb) Additional file 2:
**Web 2.** Medline search strategy for identification of report dates for first report of adverse drug reaction or first date of withdrawal. (PDF 181 kb) Additional file 3:
**Table S1.** List of medicinal products withdrawn because of adverse drug reactions. (PDF 2375 kb)

## References

[1] Furberg CD (2011). Understanding drug safety and how to maximize it for patients. JAAPA.

[2] Sullivan JW (1990). A pharmaceutical manufacturer’s perspective on reporting adverse drug experiences. Am J Hosp Pharm.

[3] Anonymous (2011). New contraindication added to Reclast drug label. Reactions Weekly.

[4] Thomas SK, Hodson J, McIlroy G, Dhami A, Coleman JJ (2013). The impact of direct healthcare professional communication on prescribing practice in the UK hospital setting: an interrupted time series analysis. Drug Saf.

[5] Eyal N (2012). Reconciling informed consent with prescription drug requirements. J Med Ethics.

[6] Food and Drug Administration. Regulatory Procedures Manual – October 2013. Chapter 7 Recall Procedures. http://www.fda.gov/downloads/ICECI/ComplianceManuals/RegulatoryProceduresManual/UCM074312.pdf. Accessed 20 Mar 2015.

[7] Onakpoya IJ, Heneghan CJ, Aronson JK (2015). Delays in the post-marketing withdrawal of drugs to which deaths have been attributed: a systematic investigation and analysis. BMC Med.

[8] Talbot J, Waller P (2004). Stephens’ Detection of New Adverse Drug Reactions.

[9] Pharmaceutical Manufacturing Encyclopedia. Norwich, NY: William Andrew Publishing; 2007.

[10] The Merck Index: An Encyclopedia of Chemicals, Drugs, and Biologicals. Whitehouse Station, NJ: Merck Sharp & Dohme Corp, a subsidiary of Merck & Co, Inc.; 2013.

[11] Siramshetty VB, Nickel-Seeber J, Omieczynski C, Gohlke B-O, Drwal MN, Preissner R. WITHDRAWN—a resource for withdrawn and discontinued drugs Nucleic Acids Res (Database issue 2015); NAR. http://cheminfo.charite.de/withdrawn/. Accessed Nov–Dec, 2015. 10.1093/nar/gkv1192 PMC4702851 26553801

[12] Medicines and Healthcare products Regulatory Authority. A guide to what is a medicinal product. MHRA Guidance Note No. 8. Revised November 2012. https://www.gov.uk/government/uploads/system/uploads/attachment_data/file/398998/A_guide_to_what_is_a_medicinal_product.pdf. Accessed 12 Oct 2014.

[13] OCEBM Levels of Evidence Working Group. The Oxford 2011 Levels of Evidence. Oxford Centre for Evidence-Based Medicine. http://www.cebm.net/index.aspx?o=5653. Accessed 18 Jun 2015.

[14] WHO Collaborating Centre for Drug Statistics Methodology. Complete ATC/DDD Index 2008. http://www.whocc.no/atc_ddd_index/. Accessed 21 Apr 2013.

[15] Isah AO, Pal SN, Olsson S, Dodoo A, Bencheikh RS (2012). Specific features of medicines safety and pharmacovigilance in Africa. Ther Adv Drug Saf.

[16] Nwokike J, Choi HL. Safety of medicines in sub-Saharan Africa: assessment of pharmacovigilance systems and their performance. Africa Pharmacovigilance Meeting: Ensuring quality and safety of medicines in sub-Saharan Africa. Nairobi, Kenya: 2012. https://africapv2012.files.wordpress.com/2012/05/day-1_2_findings-from-the-ssa-study_nwokike.pdf. Accessed 26 Nov 2015.

[17] Appiah B (2012). Africa struggles to improve drug safety. CMAJ.

[18] Loke YK, Price D, Derry S, Aronson JK (2006). Case reports of suspected adverse drug reactions – systematic literature survey of follow-up. BMJ.

[19] World Health Organization (2014). African Medicines Agency: Setting Milestones Towards its Establishment, 1 April 2014. AUC/WHO/2014/Doc.2.

[20] Menkes DB (1997). Hazardous drugs in developing countries. BMJ.

[21] Whitehead M, Dahlgren G, Evans T (2001). Equity and health sector reforms: can low-income countries escape the medical poverty trap?. Lancet.

[22] Saini P, Loke YK, Gamble C, Altman DG, Williamson PR, Kirkham JJ (2014). Selective reporting bias of harm outcomes within studies: findings from a cohort of systematic reviews. BMJ.

[23] Public Citizen. Petition to Ban the Diet Drug Sibutramine (Meridia). http://www.fda.gov/ohrms/dockets/dailys/02/Mar02/032202/02p-0120_cp00001_vol1.pdf. Accessed 11 Apr 2015.

[24] Saito M, Hirata-Koizumi M, Miyake S, Hasegawa R (2005). Withdrawal of cerivastatin revealed a flaw of post-marketing surveillance system in the United States. Kokuritsu Iyakuhin Shokuhin Eisei Kenkyusho Hokoku.

[25] Anonymous (2004). US pharmacovigilance system in need of an overhaul?. Reactions Weekly.

[26] Williams D, Feely J (1999). Underreporting of adverse drug reactions: attitudes of Irish doctors. Ir J Med Sci.

[27] Martin RM, Kapoor KV, Wilton LV, Mann RD (1998). Underreporting of suspected adverse drug reactions to newly marketed (“black triangle”) drugs in general practice: observational study. BMJ.

[28] Brvar M, Fokter N, Bunc M, Mozina M (2009). The frequency of adverse drug reaction related admissions according to method of detection, admission urgency and medical department specialty. BMC Clin Pharmacol.

[29] Pérez García M, Figueras A (2011). The lack of knowledge about the voluntary reporting system of adverse drug reactions as a major cause of underreporting: direct survey among health professionals. Pharmacoepidemiol Drug Saf.

[30] Vallano A, Cereza G, Pedròs C, Agustí A, Danés I, Aguilera C (2005). Obstacles and solutions for spontaneous reporting of adverse drug reactions in the hospital. Br J Clin Pharmacol.

[31] Irujo M, Beitia G, Bes-Rastrollo M, Figueiras A, Hernández-Díaz S, Lasheras B (2007). Factors that influence under-reporting of suspected adverse drug reactions among community pharmacists in a Spanish region. Drug Saf.

[32] Hasford J, Goettler M, Munter KH, Müller-Oerlinghausen B (2002). Physicians’ knowledge and attitudes regarding the spontaneous reporting system for adverse drug reactions. J Clin Epidemiol.

[33] Pedrós C, Vallano A, Cereza G, Mendoza-Aran G, Agustí A, Aguilera C (2009). An intervention to improve spontaneous adverse drug reaction reporting by hospital physicians: a time series analysis in Spain. Drug Saf.

[34] Lopez-Gonzalez E, Herdeiro MT, Piñeiro-Lamas M, Figueiras A, GREPHEPI group (2015). Effect of an educational intervention to improve adverse drug reaction reporting in physicians: a cluster randomized controlled trial. Drug Saf.

[35] Herdeiro MT, Ribeiro-Vaz I, Ferreira M, Polónia J, Falcão A, Figueiras A (2012). Workshop- and telephone-based interventions to improve adverse drug reaction reporting: a cluster-randomized trial in Portugal. Drug Saf.

[36] Figueiras A, Herdeiro MT, Polónia J, Gestal-Otero JJ (2006). An educational intervention to improve physician reporting of adverse drug reactions: a cluster-randomized controlled trial. JAMA.

[37] Biagi C, Montanaro N, Buccellato E, Roberto G, Vaccheri A, Motola D (2013). Underreporting in pharmacovigilance: an intervention for Italian GPs (Emilia-Romagna region). Eur J Clin Pharmacol.

[38] Tapsfield J, Mathews T, Lungu M, van Oosterhout JJ (2011). Underreporting of side effects of standard first-line ART in the routine setting in Blantyre. Malawi Malawi Med J.

[39] Kiguba R, Karamagi C, Waako P, Ndagije HB, Bird SM (2014). Recognition and reporting of suspected adverse drug reactions by surveyed healthcare professionals in Uganda: key determinants. BMJ Open.

[40] Avery AJ, Anderson C, Bond CM, Fortnum H, Gifford A, Hannaford PC (2011). Evaluation of patient reporting of adverse drug reactions to the UK Yellow Card Scheme: literature review, descriptive and qualitative analyses, and questionnaire surveys. Health Technol Assess.

[41] Furberg BD, Furberg CD, Furberg BD, Furberg CD (2007). How are adverse drug reactions measured?. Evaluating Clinical Research: All that glitters is not gold.

[42] Khan LM, Al-Harthi SE, Osman AM, Sattar MA, Ali AS (2015). Dilemmas of the causality assessment tools in the diagnosis of adverse drug reactions. Saudi Pharm J.

[43] United States Food and Drug Administration. Summary of NDA Approvals & Receipts, 1938 to the present. http://www.fda.gov/%20AboutFDA/WhatWeDo/History/ProductRegulation/SummaryofNDAApprovalsReceipts1938tothepresent/default.htm#Notes. Accessed 25 Nov 2015.

[44] Rawson NS (2013). New drug approval times and safety warnings in the United States and Canada, 1992-2011. J Popul Ther Clin Pharmacol.

[45] McNaughton R, Huet G, Shakir S (2014). An investigation into drug products withdrawn from the EU market between 2002 and 2011 for safety reasons and the evidence used to support the decision-making. BMJ Open.

[46] Fung M, Thornton A, Mybeck K, Wu J, Hornbuckle K, Muniz E (2001). Evaluation of the characteristics of safety withdrawal of prescription drugs from worldwide pharmaceutical markets −1960 to 1999. Drug Inf J.

[47] Abraham J, Davis C (2005). A comparative analysis of drug safety withdrawals in the UK and the US (1971–1992): implications for current regulatory thinking and policy. Soc Sci Med.

[48] Ninan B, Wertheimer A (2012). Withdrawing drugs in the US verses other countries. Innov Pharm.

[49] Bakke OM, Wardell WM, Lasagna L (1984). Drug discontinuations in the United Kingdom and the United States, 1964 to 1983: Issues of safety. Clin Pharmacol Ther.

[50] World Health Organization. List of Globally identified Websites of Medicines Regulatory Authorities. http://www.who.int/medicines/areas/quality_safety/regulation_legislation/ListMRAWebsites.pdf. Accessed 25 Nov 2015.

[51] NHS Choices. The Antibiotic Awareness Campaign. http://www.nhs.uk/NHSEngland/ARC/Pages/AboutARC.aspx. Assessed 6 Mar 2015.

[52] Auta A, Omale S, Folorunsho TJ, David S, Banwat SB (2012). Medicine vendors: self-medication practices and medicine knowledge. N Am J Med Sci.

[53] Bamgboye EA, Amoran OE, Yusuf OB (2006). Self medication practices among workers in a tertiary hospital in Nigeria. Afr J Med Sci.

