# Correction to: Post-marketing withdrawal of 462 medicinal products because of adverse drug reactions: a systematic review of the world literature

**DOI:** 10.1186/s12916-019-1294-9

**Published:** 2019-03-02

**Authors:** Igho J. Onakpoya, Carl J. Heneghan, Jeffrey K. Aronson

**Affiliations:** Centre for Evidence-based Medicine, Nuffield Department of Primary Care Health Sciences, University of Oxford, New Radcliffe House, Radcliffe Observatory Quarter, Oxford, OX2 6GG UK

**Correction to: BMC Med**

**https://doi.org/10.1186/s12916-016-0553-2**

The original article [1] contains a minor error whereby the dates for year of first launch and year of first report of adverse reaction for iophendylate in e-Appendix Table 1 are mistakenly presented as 1946 and 1975 respectively. The actual dates for these are 1944 and 1945 respectively. The correct version of e-Appendix Table 1 is shown in the Additional file 1. This Table should be taken into account over the version of e-Appendix Table 1 as shown in the original article [1].

## Additional file

Additional file 1: E-appendix **Table 1.** List of medicinal products withdrawn because of adverse drug reactions. (DOCX 157 kb)
