# Supplementary material for: Post-marketing withdrawal of 462 medicinal products because of adverse drug reactions: a systematic review of the world literature
Source: BMC Med. 2016 Feb 4;14:10. doi: 10.1186/s12916-016-0553-2 (PMC4740994; doi:10.1186/s12916-016-0553-2)
Supplement: Additional file 1: — Web 1. List of English Language drug regulatory websites searched for information on medicinal products withdrawn from the market because of adverse drug reactions. (PDF 299 kb) [file 12916_2016_553_MOESM1_ESM.pdf]

Additional file 1: Web 1. List of English Language drug regulatory websites searched for information on medicinal products withdrawn from the market because of adverse drug reactions.

1. ***Australia:*** Therapeutic Goods Administration (TGA): https://www.tga.gov.au/
2. ***Bahamas:*** Bahamas National Drug Agency (BNDA): http://www.corp.phabahamas.org/hospitals-services/bnda/
3. ***Bhutan:*** Drug Regulatory Authority: http://dra.gov.bt/
4. ***Botswana:*** Ministry of Health, Republic of Botswana: http://www.moh.gov.bw/ (no Medicines Regulatory Authority)
5. ***Canada:*** Health Canada Drug Product Database (DPD): http://www.hc-sc.gc.ca/dhp-mps/prodpharma/databasdon/index-eng.php
6. ***Europe:*** European Medicines Agency (EMA): http://www.ema.europa.eu/ema/
7. ***Gambia:*** Medicine Regulatory Authority newly established
8. ***Ghana:*** Ghana Food and Drugs Authority (FDA): http://www.fdaghana.gov.gh/
9. ***India:*** Central Drugs Standard Control Organization (CDSCO): http://www.cdsco.nic.in/forms/Default.aspx
10. ***Jamaica:*** Ministry of Health, Kingston, Jamaica: http://moh.gov.jm/?s=drug+regulation
11. ***Kenya:*** Pharmacy and Poisons Board, Kenya: http://pharmacyboardkenya.org/
12. ***Liberia:*** Liberian Medicines Health Products Regulatory Authority: https://healthresearchweb.org/en/liberia/research_regulation_4284 (no drug information; email delivery system failed)
13. ***Malta:*** Malta Medicines Authority: http://www.medicinesauthority.gov.mt/home?l=1
14. ***Namibia:*** Namibia Medicines Regulatory Council: http://www.nmrc.com.na/
15. ***New Zealand:*** New Zealand Medicines and Medical Devices Safety Authority: http://www.medsafe.govt.nz/index.asp
16. ***Nigeria:*** National Agency for Food and Drug Administration and Control (NAFDAC): http://www.nafdac.gov.ng/
17. ***Pakistan:*** Drug Regulatory Authority of Pakistan (DRAP): http://www.dra.gov.pk/gop/index.php?q=aHR0cDovLzE5Mi4xNjguNzAuMTM2L2RyYXAv
18. ***Philippines:*** Food and Drug Administration, Philippines (FDA): http://www.fda.gov.ph/
19. ***Rwanda:*** Ministry of Health, Republic of Botswana: http://www.moh.gov.rw/index.php?id=2 (no Medicines Regulatory Authority)
20. ***Sierra Leone:*** Pharmacy Board of Sierra Leone: http://www.pharmacyboard.gov.sl/
21. ***Singapore:*** Health Sciences Authority: http://www.hsa.gov.sg/content/hsa/en.html
22. ***South Africa:*** South Africa Medicines Control Council (MCC): http://www.mccza.com/
23. ***Sri Lanka:*** Cosmetics, Devices and Drug Regulatory Authority: http://www.cdda.gov.lk/
24. ***Tanzania:*** Tanzania Food and Drugs Authority (TFDA): http://www.tfda.or.tz/
25. ***Thailand:*** Food and Drug Administration, Thailand: http://www.fda.moph.go.th/eng/index.stm
26. ***Trinidad and Tobago:*** Ministry of Health, Trinidad and Tobago: http://www.health.gov.tt/sitepages/default.aspx?id=93
27. ***Uganda:*** National Drug Authority: http://www.nda.or.ug/

28. ***United Kingdom:*** Medicines and Healthcare products Regulatory Agency (MHRA): https://www.gov.uk/government/organisations/medicines-and-healthcare-products-regulatory-agency
29. ***USA:*** Food and Drug Administration (FDA): http://www.fda.gov/
30. ***Zambia:*** Pharmaceutical Regulatory Authority (PRA): http://www.zamra.co/
31. ***Zimbabwe:*** Medicines Control Authority of Zimbabwe (MCAZ): http://www.mcaz.co.zw/
