# Supplementary material for: Post-marketing withdrawal of 462 medicinal products because of adverse drug reactions: a systematic review of the world literature
Source: BMC Med. 2016 Feb 4;14:10. doi: 10.1186/s12916-016-0553-2 (PMC4740994; doi:10.1186/s12916-016-0553-2)
Supplement: Additional file 2: — Web 2. Medline search strategy for identification of report dates for first report of adverse drug reaction or first date of withdrawal. (PDF 181 kb) [file 12916_2016_553_MOESM2_ESM.pdf]

Additional file 2: Web 2. Medline search strategy for identification of report dates for first report of adverse drug reaction or first date of withdrawal

“[Drug name]” AND “toxicity”

“[Drug name]” AND “adverse”

“[Drug name]” AND “side effect”

“[Drug name]” AND “poison”

“[Drug name]” AND “fatal*”

“[Drug name]” AND “fatality”

“[Drug name]” AND “death”

“[Drug name]” AND “withdrawal”

“[Drug name]” AND “withdrawn”

“[Drug name]” AND “recall”

“[Drug name]” AND “voluntary recall”

“[Drug name]” AND “banned”

“[Drug name]” AND “prohibited”

“[Drug name]” AND “remov*”

“[Drug name]” AND “discontinued”

“[Drug name]” AND “refus*”

[Sort by Publication date]
