# Supplementary material for: Post-marketing withdrawal of 462 medicinal products because of adverse drug reactions: a systematic review of the world literature
Source: BMC Med. 2016 Feb 4;14:10. doi: 10.1186/s12916-016-0553-2 (PMC4740994; doi:10.1186/s12916-016-0553-2)
Supplement: Additional file 3: — Table S1. List of medicinal products withdrawn because of adverse drug reactions. (PDF 2375 kb) [file 12916_2016_553_MOESM3_ESM.pdf]

E-appendix Table 1: List of medicinal products withdrawn because of adverse drug reactions.

Medicinal product	Class	Mechanism of action	Therapeutic indication	Launch date	Year of first ADR report	Year first withdrawn	Countries withdrawn	Reason for withdrawal	Level of evidence†
Acetanilide	Analgesic	Metabolised to paracetamol	Analgesia	1886	1940	1971	Japan	Aplastic anemia	4
Acetarsol	Antimicrobial	Unknown	Syphilis, trichomoniasis	1921	1941	1982	Mauritius	Hematologic	4
Acetylfuratrizine	Antimicrobial	Damages ribosomal proteins	Bacterial infection	1962	1977	1977	Japan, Saudi Arabia, Venezuela	Superseded by safer products: carcinogenic	5*
Acitretin	Retinoid	Binds to the retinoic acid receptor (RAR)	Psoriasis	1989	1989	1990	Netherlands, France	Teratogenicity	5*
Acridine derivatives	Antimicrobial		Topical disinfectant	1922	1975	1979	Denmark, Venezuela	Mutagenic	5*
Adenosine phosphate	Antiarrhythmic	Direct nodal inhibition	Cardiac arrhythmia	1930	1957	1973	USA	Cardiovascular	4
Alatrofloxacin	Antimicrobial	Inhibit DNA gyrase	Bacterial infection	1997	1998	2000	Armenia, Singapore, Europe	Liver	4
Alclofenac	Analgesic	Inhibit COX-1 & COX-2	Analgesia	1972	1974	1977	Ireland, Cyprus, Germany, UK, Italy, New Zealand, Egypt, Greece, Denmark, Indonesia, India, Jordan, Morocco	Immunologic, liver, skin, urinary tract	4
Aliskiren	Antihypertensive	Direct renin inhibitor	Hypertension	2007	2011	2011	Europe	Angioedema	4
Alosetron‡	Antispasmodic	5HT3 receptor antagonist	Irritable bowel syndrome	2000	2000	2000	USA	Gastrointestinal	4
Alphacetylmethadol	Analgesic	OP1 receptor agonist	Analgesia	1993	2001	2003	USA	Cardiovascular	4
Alpidem‡	Anxiolytic	GABA-A receptor antagonist	Anxiety	1991	1994	1994	France	Liver	4
Althesin (alphadolone/alphaxolone)	Sedative-hypnotic	Potentiation of the GABA-A chloride channel	Anesthesia	1972	1973	1984	UK, Germany, France	Immunologic	4
Amfepramone	Psychostimulant	Norepinephrine releasing agent	Obesity	1957	1974	1975	Turkey, Sweden, Oman, UAE, Norway, Venezuela, EU, France, UK	Cardiotoxicity	4
Amfetamine	Psychostimulant	TAAR1 agonist	Obesity	1939	1957	1973	USA, UAE, Turkey, Oman, Malaysia, Nigeria	Drug dependence	4
Amineptine	Antidepressant	Dopamine receptor antagonist	Depression	1960	1984	1999	France, Thailand, UAE, Bangladesh, Morocco, Vietnam	Drug dependence, liver, skin	4
Aminoglutethimide	Anxiolytic	Steroid synthesis inhibitor	Anxiety	1960	1966	1966	US, Saudi Arabia, Norway	Endocrine	4

Aminophenazone ‡ (aminopyrine)	Analgesic	Inhibit COX-1 & COX-2	Analgesia	1887	1936	1965	10 European, 14 Asian, & 4 African countries; Australia; USA; Canada; Chile; Brazil; Venezuela	Hematologic, carcinogenic	4
Aminophylline	Anti-asthmatic	Nonselective phosphodiesterase inhibitor	Asthma	1908	1955	1992	Netherlands	Immune-mediated reactions	4
Aminorex fumarate	Psychostimulant	Catecholamine release	Obesity	1962	1967	1967	Germany, Venezuela	Respiratory	4
Amobarbital‡	Sedative-hypnotic	GABA-A receptor agonist	Hypnosedation	1961	1979	1985	Sweden, New Zealand	Respiratory depression	4
Amoproxan	Antianginal	Nonselective β-blocker	Angina	1969	1970	1970	France	Sensory systems, skin	4
Anabolic steroids	Hormones	Androgen-receptor modulation	Aplastic anemia, breast cancer, osteoporosis	1952	1966	1989	Thailand, India	Androgenic effects	4
Anagestone acetate	Hormones	Inhibition of ovulation	Contraception	1968	1969	1969	Worldwide	Tumorigenicity	5*
Anti-D immunoglobulin	Immune prophylaxis	Binds to the erythrocyte D antigen	Idiopathic thrombocytopenic purpura	1968	2000	2009	Europe	Safety concerns: hemoglobinemia,	4
Anti-histamine (topical)	Phenothiazine derivatives	H1-antagonism	Hypersensitivity	1942	1947	1986	Malaysia, Sri Lanka	Immune-mediated reactions	4
Antrafenine‡	Analgesic	COX-1 & COX-2 inhibition	Analgesia	1977	1979	1984	France	Urinary tract	4
Aprobarbital‡	Sedative-hypnotic	GABA-A receptor receptor modulation	Hypnosedation	1920	1966	1985	Sweden	Respiratory depression	4
Aprotinin	Antifibrinolytic	Pancreatic trypsin inhibitor	Bleeding	1959	1964	2007	Worldwide	Anaphylaxis	4
Arsenate	Chemotherapeutic agent	Oncoprotein expression	Cancer	1951	1973	1988	Austria, Spain, Italy, France, Philippines	Drug abuse, carcinogenic	3
Aspirin	Analgesic	COX-1 & COX-2 inhibition	Analgesia	1899	1970	1986	UK, US, Hong Kong, Nigeria, Spain	Liver	3
Astemizole	Antihistamine	H1-receptor antagonist	Allergy	1977	1986	1987	Norway, New Zealand, Philippines, USA, Canada, South Africa, UAE, Mauritius, Brunei, Tanzania, Armenia, Brazil, Spain, Argentina, Singapore, India, Chile	Cardiovascular, drug interactions	4
Azaribine‡	Antipsoriatic	Inhibition of orotidine-5'-monophosphate decarboxylase	Psoriasis	1975	1976	1976	USA; Thailand; Mauritius; Saudi Arabia; Venezuela	Hematologic, psychiatric	4
Barbital	Sedative-hypnotic	GABA-A receptor modulation	Hypnosedation	1903	1913	1971	Italy	Respiratory depression	4
Beclobrate‡	Antilipemic	Activates PPAR	Hyperlipidemia	1985	1990	1990	Switzerland	Hepatotoxicity	4
Bendazac	Analgesic	COX-1 & COX-2 inhibition	Analgesia	1983	1987	1993	Spain	Liver	4
Benfluorex‡	Psychostimulant	Fenfluramine analogue	Obesity	1976	2003	2009	Europe	Cardiotoxicity	3
Benoxaprofen‡	Analgesic	COX-1 & COX-2 inhibition	Analgesia	1980	1982	1982	Worldwide	Liver, skin, urinary tract	4

Benzarone‡	Thrombolytic	Antagonistic on mediators of muscle contraction, direct action on smooth muscle cells	Varicose veins	1964	1987	1992	Germany, Portugal, France	Liver	4
Benzbromarone	Uricosuric agent	Xanthine oxidase inhibitor	Gout	1976	1994	2003	Portugal, France	Liver	4
Benziodarone	Uricosuric agent	Inhibits hURAT1	Gout	1962	1964	1964	UK, France, Spain	Liver	4
Benzydamine (Difflam)	Analgesic	COX-1 & COX-2 inhibition	Analgesia	1967	1980	1995	Germany	Skin, psychiatric, sensory	4
Benzyl alcohol‡	Antimicrobial	Alteration of bacterial membrane permeability	Decontamination of IV lines in neonates	1972	1981	1982	Israel, USA, Oman, Iraq	Metabolic acidosis	4
Benzylpenicillin sodium (topical preparations)	Antimicrobial	Inhibits cell wall biosynthesis	Bacterial infection	1944	1953	1972	USA, Italy, Philippines, Ethiopia, Bangladesh, India, Chile, Cyprus, Spain, Thailand, Venezuela	Skin, immune	4
Bepridil‡	Antiarrhythmic	Calcium channel blockers	Cardiac arrhythmia	1981	1982	2004	USA	Cardiovascular	4
Beta-ethoxyacetanilide	Analgesic	Metabolized to paracetamol?	Analgesia	1886	1978	1986	Germany	Tumorigenicity, urinary tract	5*
Bezitramide‡	Analgesic	Unknown	Analgesia	1961	1983	2004	Netherlands	Overdose	4
Bicalutamide‡	Chemotherapeutic agent	Androgen receptor modulation	Prostate cancer	1995	2001	2003	Canada, UK	Accelerated deaths	2
Bismuth salts‡	Antidyspepsia	Unclear. Forms insoluble complexes	Dyspepsia	1875	1930	1978	France; Egypt; Japan; Greece; Austria; Bangladesh; Turkey; Mauritius; Oman; Cuba; India	Nervous, cardiovascular	4
Bithionol	Antihelminth	Exact mechanism unclear	Worm infestation	1958	1960	1967	USA, Canada, Japan	Skin	4
Boric acid and borates‡	Antimicrobial	Destruction or inhibition of growth of pathogenic organisms	Skin rash	1875	1945	1990	Ireland, Malaysia, Korea, Philippines, Thailand, Germany, Japan, Costa Rica, UK, India, Peru, USA	Deaths from neurotoxicity	4
Bovine tissue-derived medicines‡	Various: heparin, glucagon, insulin, blood factors	Various	Various	1982	1986	1989	Ireland, Switzerland, France	Neurotoxicity	4
Bromfenac‡	Analgesic	COX-1 & COX-2 inhibition	Analgesia	1997	1998	1998	USA, Saudi Arabia	Liver	4
Bromisoval	Sedative-hypnotic	Similar to barbiturates	Hypnosedation	1909	1930	1987	Netherlands	Drug dependence	4
Bromocriptine mesylate‡	Anti-lactation	D2 and D3 agonist	Lactation prevention	1976	1983	1989	USA	Cardiovascular, drug interactions, nervous system, pregnancy, psychiatric	4
Brotizolam	Sedative-hypnotic	GABA-A receptor modulation	Hypnosedation	1982	1983	1989	UK	Tumorigenicity	5*
Broxyquinoline	Antiprotozoal	Production of free radicals	Amoebiasis	1960	1968	1970	Japan, UAE, Saudi Arabia	Sensory	4
Bucetin	Analgesic	Phenacetin analogue	Analgesia	1968	1985	1986	Germany	Urinary tract: Mutagenic, carcinogenic	5*

Budipine	Antiparkinsonian	Muscarinic & NMDA receptor antagonist	Parkinson's	1979	2000	2000	Germany	Cardiovascular	4
Bufexamac	Analgesic	COX-1 & COX-2 inhibition	Analgesia	1973	1973	1990	France	Skin	4
Buflomedil‡	Vasodilator	α-adrenergic blockade	Peripheral arterial occlusive disease	1970	1981	2006	France, Europe	Neurotoxicity; cardiotoxicity	4
Buformin‡	Hypoglycemic	Reduce gluconeogenesis	Diabetes	1950	1969	1978	Germany, Austria, Belgium, Ireland	Metabolism	4
Bumadizone injection	Analgesic	COX-1 & COX-2 inhibition	Rheumatism	1972	1978	1986	Oman	Hematologic	5*
Bunamiodyl‡	Radiography	Selective secretion in bile	Radiography	1958	1962	1964	USA, Sweden, Venezuela	Kidney	4
Buprenorphine‡	Analgesic	Agonist–antagonist opioid receptor modulator	Analgesia	1978	1983	1986	Egypt	Fatalities (IV use)	4
Bupropion	Antidepressant	Norepinephrine-dopamine reuptake inhibitor	Depression	1985	1985	1986	USA	Nervous system	4
Butamben	Anesthetic	↓ neuronal membrane permeability to sodium ions	Local anesthesia	1923	1947	1964	UK	Allergic, psychiatric, skin	4
Cadralazine	Antihypertensive	Peripheral arteriolar vasodilator	Hypertension	1989	1991	1992	Norway	Immunologic	2
Camazepam	Sedative-hypnotic	GABA-A receptor modulation	Hypnosedation	1978	1984	1984	Netherlands	Immunologic	4
Canrenone	Antihypertensive	Diuresis, aldosterone antagonist	Aldosteronism, CHF, hypertension	1966	1976	1986	Germany	Tumorigenicity	5*
Carbinoxamine	Antihistamine	Competes with free histamine for binding at HA-receptor sites	Allergy	1953	1987	2008	Iraq	Neurotoxicity	4
Carisoprodol	Muscle relaxant	Unknown	Sprain, muscle injury	1959	2002	2007	Sweden, EU, Indonesia	Abuse	4
Cartilage + bone marrow	Antiarthritic		Degenerative joint disease	1960	1989	1992	Germany	Skin	3
Catechic extract‡	Antiinflammatory	Unclear	Benign prostatic hyperplasia	1972	1979	1982	France	Hematologic	4
Cefaloridine	Antimicrobial	Disruption of cell wall synthesis	Bacterial infection	1964	1969	1989	Spain	Nephrotoxic	4
Celecoxib (Onsenal) ‡	Analgesic	COX-2 inhibitor	Familial adenomatous polyposis	2003	2006	2011	Europe	Increased risk of serious cardiovascular events	3
Cell preparations‡	Antiageing		Adjuvant; antiaging	1931	1957	1987	Austria, Germany, Switzerland	Immunologic	4
Cerivastatin‡	Antilipemic	HMG CoA reductase inhibition	Hyperlipidemia	1997	1998	2001	Worldwide	Renal, musculoskeletal	4
Chenodeoxycholic acid	Antilipemic	↓ cholesterol saturation of bile	Cholelithiasis (gallstones)	1975	1976	1987	Norway	Tumorigenicity	5*
Chloral hydrate	Sedative-hypnotic	GABA receptor modulation	Hypnosedation	1869	1986	2001	USA, France, India	Tumorigenicity	5*
Chloramphenicol	Antimicrobial	Disruption of cell wall synthesis	Bacterial infection	1947	1950	1978	France, Spain, Hungary, India	Hematologic	4

Chlormadinone acetate	Hormones	Inhibition of ovulation	Contraception	1965	1970	1972	USA, UK, Italy, Egypt, Venezuela	Tumorigenicity	5*
Chlormezanone	Muscle relaxant	GABA receptor modulation	Anxiolytic, muscle sprain & injury	1960	1983	1996	Worldwide	Skin, drug dependence, liver	4
Chlornaphazine	Chemotherapeutic agent	Cell hydrolysis	Polycythemia & Hodgkin's disease	1964	1964	1964	Denmark, Venezuela	Tumorigenicity	4
Chloroform (trichloromethane)	Anesthetic	Depression of the respiratory centres	Anesthesia	1847	1976	1976	Greece, Turkey, Japan, USA, Panama, Saudi Arabia, Brazil, Italy, Canada, Norway, Philippines, UK, Denmark, NZL, Ethiopia, Zimbabwe, Bangladesh, Dominican Republic, Belgium, Nigeria, Ireland, Oman, Cuba, India	Cardiovascular, liver, tumorigenicity, urinary tract	5*
Chloroquine	Antiprotozoal	Cell lysis	Malaria	1939	1959	1975	Japan, Guinea Bissau	Eyes	4
Chlorphentermine	Psychostimulant	TAAR1 agonist	Obesity	1962	1970§	1969	Germany, Venezuela	Respiratory, cardiovascular	5*
Chorionic gonadotrophin	Hormones	Spermatogenesis	Hypogonadism	1934	1961	1972	USA	Immunologic	4
Cianidanol‡	Antioxidant	Free radical scavenger	Hepatitis	1976	1980	1985	Worldwide	Hematologic	4
Cincophen	Analgesic	Adrenal stimulation	Gout	1910	1923	1991	Germany, Italy	Liver	4
Cinepazide	Vasodilator	Phosphodiesterase inhibition	Cerebrovascular disease	1974	1985	1988	Egypt, Spain	Hematologic	4
Cisapride monohydrate‡	Prokinetic agent	5-HT4 receptor agonist	Gastro-esophageal reflux	1993	1993	2000	Philippines, Oman, USA, Germany, UK, Serbia, Mauritius, Canada, Brunei, Turkey, Indonesia, Singapore, Japan, Cuba, Bahrain, Australia, Armenia, India	Cardiovascular, drug interactions	4
Cliobutinol	Antitussive	Unclear	Cough	1961	2004	2007	Europe, Argentina	Cardiovascular	4
Clioquinol	Antimicrobial	Inhibition of DNA replication	Diarrhea	1934	1964	1970	Japan, Norway, Sweden, Denmark, UAE, Nigeria, Bangladesh, Philippines, Italy, Nepal, Dominican Republic, Zimbabwe, Spain, Hong Kong, Ethiopia, Honduras, Oman, Pakistan, Ghana, Libya, Bahrain, Netherlands, Saudi Arabia, Canada	Nervous system	4
Clobenzorex	Psychostimulant	Similar to amphetamine	Obesity	1966	1986	2000	Mauritius, USA	Drug abuse, psychiatric	4
Clofenotane	Pesticide	Interfere with voltage-gated sodium channel	Pediculosis	1945	1969	1972	USA	Carcinogenicity	5*
Clofibrate‡	Antilipemic	Activates PPAR	Hyperlipidemia	1967	1973	1978	Germany, Israel, Norway, Bangladesh	Accelerated deaths	4

Cloforex	Psychostimulant	Similar to amphetamine	Obesity	1965	1967	1967	Germany, Sweden, Venezuela	Cardiovascular, drug abuse, psychiatric	4
Clomacron	Analgesic	COX-1 & COX-2 inhibition	Analgesia	1966	1970	1982	UK	Liver	4
Clometacin‡	Analgesic	COX-1 & COX-2 inhibition	Analgesia	1971	1981	1990	France	Liver, skin, urinary tract	4
Clozapine	Antipsychotic	Unknown	Schizophrenia	1972	1975	1975	Finland, Singapore, Norway	Cardiovascular, hematologic	4
Cobalt	Hematinic	As cobalamin	Iron-deficiency anemia	1951	1954	1967	USA, Kuwait	Cardiovascular, liver	4
Codeine	Analgesic	G-protein receptor modulation	Analgesia	1832	1951	1985	Bangladesh, Malaysia	Abuse	4
Co-proxamol (paracetamol + dextropropoxyphene) ‡	Analgesic	?COX-3 inhibition; agonist at mu opioid receptors	Analgesia	1957	1960	2007	Europe, India, USA, Zimbabwe, New Zealand, Iraq	Overdose; respiratory depression; cardio and hepatotoxicity	4
Coumarin (synthetic) ‡	Anticoagulant	Vitamin K reductase inhibition	Lymphedema post-radiation	1996	1996	1996	France, Australia	Liver	4
Cupric bisquinoline	Antiarthritic	Antiinflammatory with penicillamine complex	Rheumatism	1958	1967	1978	France	Neuromuscular	5*
Cyclandelate	Vasodilator	Direct effect on vascular smooth muscle	Raynaud's disease	1987	1989	1996	USA	Not effective for use	4
Cyclobarbital	Sedative-hypnotic	GABA-A receptor receptor agonist	Hypnosedation	1962	1974	1980	Norway, France	Drug dependence, drug overdose	4
Cyclofenil	Anti-hormone	Estrogen receptor modulation	Scleroderma	1970	1980	1987	France	Liver	3
Cyclovalone + retinol + tiratricol‡	Choleretic	?	Hyperlipidemia, dyspepsia, obesity	1964	1984	1988	France	Liver	4
Cyproheptadine	Antihistamine	Antihistamine, anticholinergic	Allergy, anorexia	1961	1977	1982	Bangladesh, Malaysia, UK	Drug abuse	4
Dalkon shield	Hormones	Prevention of fertilization	Contraception (IUD)	1971	1974	1974	USA, UK, New Zealand	Septic abortions	4
Danthron (chrysazin)	Laxative	?	Constipation	1959	1985	1987	Norway, Germany, Japan, USA, Singapore, Canada	Tumorigenicity	5*
Datura	Analgesic	Muscarinic receptor antagonist (atropine)	Asthma	1920	1962	1992	France	Drug abuse	4
Dequalinium chloride	Lozenges	?	Disinfectant	1956	1970	1984	Greece	Low efficacy, skin reactions	4
Desensitizing vaccines	Vaccines	?	Viral infection	1973	1985	1989	UK	Immunologic	4
Dexamfetamine	Psychostimulant	Adrenergic receptor agonist	Narcolepsy, ADHD	1937	1966	1973	USA, Turkey, Oman, Nigeria	Drug abuse and dependence	4

Dexfenfluramine	Psychostimulant	Serotonin receptor agonist	Obesity	1995	1995	1997	USA, Canada, Morocco, Philippines, Lithuania, India	Cardiovascular, respiratory	4
Diacetoxydiphenolisatin	Laxative	Phenacetin analogue	Diarrhea	1971	1971	1971	Australia	Liver	4
Diamthazole	Antimicrobial	?	Fungal infection	1951	1953	1972	US, France	Nervous system, psychiatric	4
Dibenzepine hydrochloride‡	Antidepressant	Norepinephrine reuptake inhibitor	Depression	1963	1967	1983	Sweden	Suicides	4
Dibromsalan	Antimicrobial	?	Bacterial infection	1962	1966	1975	USA, Japan	Skin	4
Diclofenac sodium	Analgesic	COX-1 & COX-2 inhibition	Analgesia	1979	1979	1983	Philippines, Norway	Hematologic, tumorigenicity	4
Dicycloverine	Antispasmodic	Muscarinic anticholinergic	Irritable bowel syndrome	1952	1974	1986	Bangladesh, Norway	Nervous, psychiatric	4
Dienestrol	Hormones	Estrogen receptor agonist	Hormone replacement therapy	1947	1972	1977	Austria, Italy, Kuwait, Saudi Arabia	Carcinogenicity	3
Diethyl-aminoethoxyhexestrol	Antianginal	?	Angina pectoris	1964	1970	1970	Japan	Liver	3
Diethylstilbestrol	Hormones	Uterine desensitization, local arteriolar constriction	Threatened abortion	1938	1971	1973	Panama, Austria, Kuwait, Italy	Tumorigenicity	3
Difemerine	Antispasmodic	Muscarinic antagonist	GI spasms	1967	1980	1986	Germany, France, USA	Nervous system	4
Difenoxin	Antispasmodic	Opioid receptor agonist	Diarrhea	1970	1979	1991	Pakistan, Oman, South Korea, Lebanon	Atropism	2
Difurazone	Antimicrobial	?	Bacterial infection	1966		1977	Japan, Saudi Arabia, Venezuela	Superseded by safer products	5
Dihydrostreptomycin	Antimicrobial	Protein synthesis inhibitor	Bacterial infection	1947	1949	1970	USA, Philippines, Spain, Dominican Republic, Italy, Peru	Nervous system, psychiatric, sensory systems	3
Dihydroxy-methylfuratrizine	Antimicrobial	Nitrofuran analogue	Bacterial infection	1975	1975	1977	Japan, Saudi Arabia, Venezuela	Tumorigenic	5*
Diiododiethyltin‡	Antimicrobial	?	Bacterial infection	1954	1955	1957	France	Nervous system	4
Dilevalol	Antihypertensive	Nonselective beta blocker; beta-2 receptor agonist	Hypertension	1989	1989	1990	Worldwide	Liver	4
Dinitrophenol	Organic compound	Uncoupling of oxidative phosphorylation	Obesity	1933	1933	1938[Δ]	USA	Cataracts, agranulocytosis	4
Dinoprostone	Prostaglandin	Direct vasodilator	Induction of labour	1971	1987	1990	UK	Fetal distress; uterine hypertonia	3
Diphenazine (quietidin)	Analgesic	?	Analgesia	1962	1967	1967	Hungary, Venezuela	Photosensitivity	4

Diphenoxylate	Antispasmodic	Opioid receptor agonist	Diarrhea	1956	1969	1990	Libya, Pakistan, Mexico, Nepal, Philippines, South Korea, Lebanon, Thailand	Nervous system	4
Dithiazanine iodide‡	Antihelminth	Interruption of glucose uptake in cells	Worm infestation	1959	1960	1964	USA; France; Chad; Italy; Cuba	Cardiovascular, metabolism	4
Dofetilide	Antiarrhythmic	Potassium channel blocker	Cardiac arrhythmia	1999	1999	2004	Europe	Cardiovascular	2
Dolansetron	Propulsive	5-HT3 receptor antagonist	Emesis	1997	2005	2011	Germany	Cardiovascular	4
Domperidone (injectable) ‡	Propulsive	Dopamine receptor antagonist	Emesis, GERD	1979	1982	1985	Worldwide	Cardiovascular, drug overdose, endocrine, nervous system	4
Doxycycline (IV) ‡	Antimicrobial	Protein synthesis inhibitor	Bacterial infection	1959	1984	1989	France, Morocco	Anaphylaxis	4
Doxylamine/dicyclomine	Antihistamine	H1-receptor blocker	Allergy, hyperemesis	1956	1981	1983	UK, USA	Teratogenicity	3
Drotrecogin alfa (activated)	Antithrombotic	Unclear	Sepsis	2001	2001	2011	Worldwide	Insufficient evidence; bleeding risk	1
Droperidol	Antipsychotic	Dopamine 2 receptor antagonist	Psychoses	1970	1991	2001	UK, Indonesia	Cardiovascular; deaths	4
Droxicam	Analgesic	COX-1 & COX-2 inhibition	Analgesia	1990	1992	1994	Europe	Liver	4
Ebrotidine‡	Anti-ulcer	H2 receptor antagonist	Peptic ulcer	1997	1998	1998	Spain, Peru	Liver	4
Efalizumab	Monoclonal antibody	Inhibits CD11a	Autoimmune disease	2003	2008	2009	EU, USA, Canada, Mexico	Leukoencephalopathy	4
Emetin (ipecac syrup)	Emetic	Stimulation of the CTZ, local irritation	Emesis	1912	1947	1982	Mauritius	Cardiotoxicity	3
Encainide‡	Antiarrhythmic	Na channel blocker	Cardiac arrhythmia	1985	1989	1991	UK	Cardiovascular	4
Epinephrine (topical)	Anesthetic	Vasoconstriction	Local anesthesia	1899	1963	2004	Ireland, Venezuela	Cardiovascular, nervous	4
Erythrityl tetranitrate	Antihypertensive	Nitric oxide synthesis	Angina pectoris	1955	1987	1998	USA	Insufficient evidence: skin	4
Erythromycin balleul	Antimicrobial	Protein synthesis inhibitor	Bacterial infection	1955	1975	1995	France	Teratogenic	4
Erythromycin estolate	Antimicrobial	Protein synthesis inhibitor	Bacterial infection	1958	1961	1976	Singapore, Greece, Sudan, Mauritius, Bangladesh, Bahrain, Denmark, Sweden	Liver	4
Ethanol	Antimicrobial	GABA-A receptor modulation, protein coagulation	Sedation, disinfectant	1850	1946	1985	Chile, Sri Lanka, Brazil, UAE	Neurotoxicity, abuse	4
Ethchlorvynil	Sedative-hypnotic	Unknown	Hypnosedation	1955	1959	1978	USA	Drug dependence, respiratory	4
Ethinyl estradiol	Hormones	?Gonadotropin suppression	Contraception	1943	1963	2004	Germany, France	Venous thrombosis	3

Ethyl nitrite (spirit) ‡	Antimicrobial	Increased GFR, nitric oxide synthesis	Diaphoresis, GI spasm	1850	1977	1980	USA	Methemoglobinemia	4
Ethylene dichloride (topical)	Antimicrobial		Bacterial infection	1794	1977	1978	Germany, Saudi Arabia	Carcinogenic	5*
Ethylestrenol	Hormones	Stimulate anabolism & inhibit catabolism	Testosterone deficiency	1964	1965	1982	Bangladesh	Inappropriate use in malnourished children	4
Etomidate	Anesthetic	GABA-A receptor modulation	Anesthesia	1972	1983	1985	UK	Endocrine	3
Etretinate	Retinoid	Inhibits cell differentiation & hyperkeratinization	Psoriasis	1981	1982	1992	Norway, Brazil, France, USA, Canada	Teratogenicity, tumorigenicity, death	5*
Exifone	Psychostimulant	Free radical scavenger?	Memory deficits	1988	1989	1989	France	Liver	4
Factor IX	Anti-hemophilic	Hydrolysis of disulfide bonds	Hemophilia	1952	1986	1986	Sweden	HIV	4
Factor VIII	Anti-hemophilic	Cofactor for factor IXa	Hemophilia	1965	1986	1986	UK	HIV	4
Febarbamate	Sedative-hypnotic	GABA-A receptor receptor modulation	Hypnosedation	1967	1991	1997	France	Liver	4
Felbamate‡	Anti-epileptic	NMDA receptor antagonist	Epilepsy	1993	1993	1994	USA, European Union	Hematologic, liver	4
Fenbutrazate	Psychostimulant	Phenmetrazine analogue	Obesity	1957	1963	1969	Europe	Drug abuse, psychiatric	2
Fenclofenac‡	Analgesic	COX-1 & COX-2 inhibition	Analgesia	1978	1984	1984	Worldwide	Skin, tumorigenicity, urinary tract	4
Fenclozic acid	Analgesic	COX-1 & COX-2 inhibition	Analgesia	1969	1970	1970	UK, USA	Liver	2
Fenetylline	Psychostimulant	Amphetamine + theophylline properties	ADHD, narcolepsy	1961	1962	1991	Oman, Bulgaria	Abuse	4
Fenfluramine	Psychostimulant	Serotonin receptor antagonist	Obesity	1973	1996	1997	Worldwide	Cardiovascular, respiratory	3
Fenoterol‡	Antiasthmatic	Beta 2 adrenergic agonist	Asthma	1971	1987	1990	New Zealand, Australia	Death	3
Fenproporex	Psychostimulant	Amphetamine analogue	Obesity	1966	1997	1999	Europe	Drug abuse, psychiatric	4
Fentanyl hydrochloride‡	Anagesic	μ-opioid receptor agonist	Analgesia, anesthesia	2006	2006	2009	Europe	Risk of overdose	4
Feprazone	Analgesic	COX-1 & COX-2 inhibition	Analgesia	1978	1981	1984	UK, Germany, Greece, Egypt	Liver, gastrointestinal, skin, urinary tract	4
Fipexide‡	Nootropic	TAAR1 receptor modulation?	Memory deficits	1973	1990	1991	France	Hematologic, liver	4

Floctafenine	Analgesic	COX-1 & COX-2 inhibition	Analgesia	1976	1976	1987	Belgium	Dermatologic	4
Flosequinan‡	Vasodilator	Direct relaxing effects	Congestive heart failure	1992	1992	1993	UK, USA	Death	2
Flosulide	Analgesic	COX-2 inhibitor	Analgesia	1994	1995	1996	Germany	Kidneys	5*
Flunitrazepam	Sedative-hypnotic	GABA-A receptor modulation	Hypnosedation	1974	1979	1986	South Africa, Sweden	Drug abuse	4
Fluproquazone	Analgesic	COX-1 & COX-2 inhibition	Analgesia	1979	1984	1989	USA	Liver	4
Fluvoxamine	Antidepressant	Selective serotonin re-uptake inhibitor	Obsessive Compulsive disorder; depression	1984	1987	1987	Iceland	Teratogenicity, renotoxicity	5*
Furazolidone	Antimicrobial	Disruption of bacterial DNA	Bacterial infection	1954	1965	1977	Japan, Iraq, Malaysia, South Korea, Lebanon, Yemen, India	Skin, hematologic, nervous, respiratory, sensory	4
Gadofosveset trisodium	Radiography	Binds to human serum albumin	Diagnostic imaging	1988	2006	2010	European Union	Kidney	3
Gallopamil	Antiarrhythmic	Calcium channel blockers	Cardiac arrhythmia	1983	1985	2001	Turkey	Not specified: Cardiovascular	2
Ganglioside (bovine)	Glycolipids	Dimerization of neurotrophic factor tyrosine kinases	Peripheral neuropathies	1975	1985	1989	Germany	Nervous system	3 & 5
Gatifloxacin	Antimicrobial	Inhibition of DNA gyrase	Bacterial infection	1999	2002	2011	Europe, India	Dysglycemia	3
Gelatin	Coagulant	?	Bleeding	1915	1978	1978	USA	Hematologic	4
Gemfibrozil	Anticholesterol	Activates peroxisome proliferator-activated receptor-alpha (PPARα)	Dyslipidemia	1982	1982	1987	Norway	Negative benefit-to-harm balance	2
Gemtuzumab ozogamicin‡	Monoclonal antibody	Binds to the CD33 antigen	Leukemia	2000	2010	2010	USA	Accelerated deaths	2
Genaconazole	Antimicrobial	Interferes with the fungal synthesis of ergosterol	Fungal infection	1970	1992	1992	USA	Hepatocellular cancer	5*
Gentamicin (topical)	Antimicrobial	Interruption of protein synthesis	Bacterial infection	1966	1976	1994	Netherlands, UAE	Resistance	3
Glafenine	Analgesic	COX-1 & COX-2 inhibition	Analgesia	1965	1973	1984	Worldwide	Urinary tract	4
Glucosamine sulphate (injection)	Antiarthritic	Precursor in biochemical synthesis of glycosylated proteins and lipids	Rheumatism	1980	1981	1986	Germany, Egypt	Immune: hypersensitivity	3
Glutethimide	Sedative-hypnotic	Similar to barbiturates	Hypnosedation	1955	1962	1980	Norway, Zimbabwe, Pakistan, France	Drug abuse, drug dependence, sensory systems	4
Glycosaminoglycan	Antiarthritic	?	Rheumatism	1975	1982	1982	Germany	Hematologic	3
Grepafloxacin‡	Antimicrobial	Inhibition of DNA gyrase	Bacterial infection	1997	1999	1999	Worldwide	Cardiovascular	4
Guanethidine	Antihypertensive	Sympathetic antagonist	Hypertension	1973	1978	1986	UK	Sensory systems	4

Guanofuracin	Antimicrobial	Disruption of bacterial DNA	Bacterial infection	1966		1977	Japan, Venezuela	Safer alternatives	5
HA-1A (Centoxin)	Monoclonal antibody	Endotoxin lipopolysaccharide	Sepsis	1991	1991	1993	US	Death	2
Halogenated hydroxyquinoline derivatives	Antimicrobial	Production of free radicals	Amoebic dysentery	1949	1971	1978	Denmark, Cyprus, Philippines, Bangladesh, Ghana, Turkey, Italy, Greece, Oman, UAE, India	Nervous	4
Halogenated salicylanilides	Antimicrobial	Production of free radicals	Bacterial infection	1954	1964	1975	USA, Japan	Skin, eyes	4
Heptabarb‡	Sedative-hypnotic	GABA-A receptor modulation	Convulsion	1956	1984	1984	Sweden	Drug abuse	4
Herpes simplex vaccines	Vaccines	Herpes simplex antibodies	Viral infection (HSV)	1964		1984	Germany, Saudi Arabia, Venezuela	Potential hazards	5
Hexachlorophene	Antimicrobial	Production of free radicals	Bacterial infection	1948	1968	1972	Germany, Japan, Turkey, Europe, USSR, Peru	Encephalopathy, mutagenicity, teratogenicity	3
Hexestrol	Chemotherapeutic agent	Nonsteroidal estrogen	Prostate CA	1946	1977	1977	Austria, Italy, Kuwait, Armenia, Saudi Arabia, Venezuela	Carcinogenic	4
Hexestrol bis (beta-diethylaminoethyl ether)	Vasodilator	Coronary vasodilatation	Hypertension	1952	1963	1969	Japan	Hepatotoxicity	4
Hexobarbital‡	Sedative-hypnotic	GABA-A receptor modulation	Convulsion	1945	1962	1984	Sweden	Drug abuse	4
Human placental extract	Placenta derived tissue	?	Arthritis, eczema, acne vulgaris	1956	1988	1994	Germany, UK, India	Neurotoxicity; Immune; skin	4
Hydrochlorothiazide + sotalol	Antihypertensive	Diuresis plus beta receptor antagonist	Hypertension	1970	1979	1986	France	Cardiovascular, drug interactions	4
Hydromorphone	Analgesic	Opioid receptor agonist	Analgesia	1926	1985	2005	USA, Switzerland	Overdose potential	4
Hyoscine methonitrate	Antispasmodic	Muscarinic receptor antagonist	GI spasms	1947	1964	1981	Sweden	Drug abuse	4
Ibufenac	Analgesic	COX-1 & COX-2 inhibition	Analgesia	1961	1964	1968	UK	Liver	4
Indalpine	Antidepressant	SSRI	Depression	1983	1985	1985	France	Gastrointestinal, hematologic	4
Indometacin (Osmosin) ‡	Analgesic	COX-1 & COX-2 inhibition	Analgesia	1963	1978	1983	UK	Gastrointestinal; deaths	4
Indoprofen‡	Analgesic	COX-1 & COX-2 inhibition	Analgesia	1976	1982	1983	Worldwide	Carcinogenic	4
Indoramin	Vasodilator	Alpha-1 adrenoceptor antagonist	BPH, Hypertension	1981	1986	2011	UK	Cardiovascular	4
Influenza virus, split, inactivated (pandremix)	Vaccines	Antibody production	Viral infection (influenza)	2006	2010	2010	Europe	Narcolepsy	3

Insulin, inhaled (Exubera)	Hormones	Decreased gluconeogenesis	Diabetes	2006	2008	2008	USA, Europe	Lung cancer	2
Interferon-gamma-1b‡	Immunomodulator	Binds directly to the type II interferon gamma receptor IFNGR1	Idiopathic pulmonary fibrosis	2002	2007	2007	USA	Accelerated deaths	2
Iodinated casein	Hormones	Thyroxine analogue	Obesity	1944	1964	1964	USA	Endocrine, metabolism	4
Iophendylate	Radiography		Radiocontrast imaging	1946	1976	1987	UK	Nervous system	4
Iproniazid	Antidepressant	MAOI	Depression	1952	1958	1959	Italy, Canada	Immunologic, liver	4
Isaxonine phosphate‡	Neurotrophic	Muscle reinnervation	Peripheral neuropathies	1981	1983	1984	France, Tunisia	Hematologic, liver	4
Isocarboxazid	Antidepressant	MAOI	Depression	1959	1966	1974	Japan, Cuba, Venezuela	Metabolic	4
Isoprenaline	Cardiac stimulant	Non-selective beta-adrenergic agonist	Bradycardia & heart block, asthma	1949	1968	1992	Sri Lanka, UK, Australia, New Zealand	Cardiovascular	3
Isotretinoin	Retinoid	Unknown; apoptosis	Cystic acne	1982	1983	1988	Denmark, Austria, Germany, Portugal, Norway,	Teratogenicity	3
Isoxicam‡	Analgesic	COX-1 & COX-2 inhibition	Analgesia	1983	1985	1985	Worldwide	Skin	4
Kaolin	Antidiarrheal		Diarrhea	1954	1968	1991	India, Sri Lanka	Metabolism	4
Ketoconazole‡	Antimicrobial	Inhibits the cytochrome P450 14α-demethylase	Fungal infection	1976	1981	2013	EU, Australia	Hepatotoxicity	1
Ketoprofen (gel)	Analgesic	COX-1 & COX-2 inhibition	Analgesia	1980	1983	2008	Egypt, France	Immunologic	4
Ketorolac (inj) ‡	Analgesic	COX-1 & COX-2 inhibition	Analgesia	1989	1992	1992	Germany, France, Jamaica	Gastrointestinal, skin	4
Lapdap (chlorproguanil + dapsone)	Antimalarial	Inhibits dihydrofolate reductase	Malaria	2003	2008	2008	Worldwide	Hemolytic anemia	2
Laropiprant / nicotinic acid	Antilipemic	Prostaglandin receptor blocker	Facial flushing	2008	2008	2008	Worldwide	Higher frequency of non-fatal but serious side effects	2
Lead oxide and lead salts (topical)	Astrigent		Skin tightening	1892	1977	1980	France, Denmark, Saudi Arabia, Venezuela	Nervous (encephalopathy)	4
Levacetylmethadol	Antidote	mu-opioid receptor agonist, nicotinic acetylcholine receptor antagonist	Opioid dependence	1995	2001	2001	Europe, USA	Cardiovascular	4
Levamfetamine	Psychostimulant	Amphetamine analogue	Obesity	1944	1954	1973	USA, Oman, UAE	Drug abuse and dependence	4
Levamisole‡	Antihelminth	Neuromuscular depolarizing blockade	Worm infestation	1966	1976	1999	US, Canada, Vietnam	Hematologic, nervous system	3
Levarterenol	Vasopressor	L-norepinephrine analogue	Nonhemorrhagic shock	1904	1958	1973	Ireland, Venezuela	Nervous, cardiovascular	4
Lindane	Insecticide	CNS stimulation	Head lice	1942	1972	2001	Brazil	Toxicity	4

Letrozole	Hormone antagonist	Non-steroidal aromatase inhibitor	Hormonally-responsive breast cancer	2001	2005	2011	India	Teratogenic	3
Loperamide (syrup & drops) ‡	Antidiarrheal	Opioid receptor agonist	Diarrhea	1975	1990	1990	Worldwide	Paralytic ileus	4
Loxoprofen sodium‡	Analgesic	COX-1 & COX-2 inhibition	Analgesia	1983	1993	2000	Singapore	Colonic ulceration	4
L-Tryptophan‡	Antidepressant	Converted into serotonin	Depression and sleep disorders	1963	1989	1989	Worldwide	Eosinophilia-myalgia syndrome (EMS)	4
Lumiracoxib‡	Analgesic	COX-2 inhibitor	Analgesia	2006	2007	2007	Australia, USA, Canada, Europe, New Zealand, Brazil, Philippines, Colombia	Liver	4
Lyme disease vaccine	Vaccines	Immunogenicity	Bacterial infection (Lyme disease)	1998	2001	2002	USA	Musculoskeletal (arthralgia)	3
Lynestrenol	Hormones	Inhibits gonadotropin, suppression of follicle maturation	Contraception	1962	1970	1980	Australia	Tumorigenicity	5*
Lysine amidotriazoate	Radiography		Vascular diagnostics	1975	1981	1995	Germany	Cardiovascular, hematologic, immunologic, urinary tract (safer alternatives)	5*
Mazindol	Psychostimulant	Norepinephrine & dopamine reuptake inhibitor	Obesity	1970	1980	1987	Oman	Drug abuse, psychiatric (interaction with lithium)	4
Mebanazine	Antidepressant	MAOI	Depression	1963	1964	1975	UK	Drug interactions, liver	4
Meclozine (meclizine)	Antihistamine	H1 receptor antagonist	Motion sickness	1953	1963	1963	Indonesia	Teratogenic potential	5*
Medifoxamine	Antidepressant	Dopamine reuptake inhibitor; 5HT-receptor agonist	Depression	1983	1991	1999	Morocco, France	Liver	4
Mefenorex	Psychostimulant	Amphetamine analogue	Obesity	1966	1995	1999	Europe	Drug abuse, psychiatric	4
Megestrol acetate	Hormones	Progesterone receptor agonist	Contraception	1963	1975	1976	Greece, Norway, Germany, New Zealand	Tumorigenicity	5*
Mepacrine (quinacrine)	Antimicrobial	Unclear	Non-surgical sterilization	1935	1953	1998	India	Carcinogenicity; ectopic pregnancy; possibly political	2
Mepazine	Antiepileptic	Unclear	Epilepsy	1955	1957	1970	USA	Cardiovascular, gastrointestinal, hematologic, liver, urinary tract	4
Mephenesin	Muscle relaxant	Spinal reflex inhibition	Sedation, muscle relaxation	1948	1955	1976	Japan, Saudi Arabia	Cardiotoxicity	4

Meprobamate	Muscle relaxant	GABA-A receptor modulation	Anxiety	1955	1957	1981	Sweden, Europe	Drug abuse	4
Mercurothiolate (thiomersal)	Antimicrobial	Organomercury analogue	Bacterial and fungal infections	1928	1996	1999	UK, Malaysia, Brazil	Nervous system, tumorigenicity, urinary tract	4
Mercury amide	Antimicrobial	Organomercury analogue	Disinfectant	1956	1964	1969	Japan, Brazil, Philippines, France, Nigeria, Ghana, Italy, Canada	Nervous system	4
Mercurous chloride	Antimicrobial	Organomercury analogue	Teething	1796	1949	1953	Worldwide	Nervous system	4
Mesna	Detoxifying agent	Antioxidation?	Antidote for chemotherapy	1984	1985	1991	Germany	Anaphylaxis	4
Metamfetamine (desoxyephedrine)	Psychostimulant	TAAR1 receptor agonist	ADHD, obesity	1944	1972	1973	USA, Turkey, Oman, Nigeria	Drug abuse, drug dependence	4
Metamizole (dipyrone) ‡	Analgesic	COX-1 & COX-2 inhibition	Analgesia	1921	1952	1965	9 European, 11 Asian, and 5 African countries; Australia; USA; Mexico; Canada	Hematologic, immunologic (agranulocytosis)	4
Methapyrilene	Sedative-hypnotic	Histamine and cholinergic receptors' antagonist	Insomnia	1947	1979	1979	Germany, Dominican Republic, UK, Italy, Canada, Singapore, Hong Kong, Australia, Egypt, Panama, Brazil, Philippines, UAE, India, Oman, Chile, New Zealand, USA, Venezuela	Tumorigenicity	5*
Methaqualone	Sedative-hypnotic	GABA receptor agonist	Hypnosedation	1965	1966	1979	Greece, Turkey, Oman, Zimbabwe, Pakistan, Ghana, UAE, India	Drug abuse	4
Methiodal sodium	Radiography		Urinary tract diagnostics	1964	1972	1975	Sweden	Muscle	4
Methylandrostenolone	Hormones	Protein synthesis	Tonic	1960	1982	1982	USA, France, UK, Germany,	Endocrine (masculinization)	4
Methylhexanamine (DMAA)	Nasal decongestant	Norepinephrine & dopamine transporter blockade	Nasal decongestion	1948	1950	1983	Worldwide	Cardiovascular, nervous	5*
Methylphenidate	Psychostimulant	Dopamine-norepinephrine reuptake inhibitor	ADHD	1956	1961	1982	Turkey, Oman, Nigeria	Drug abuse	4
Methylrosanilinium chloride	Antimicrobial		Bacterial & fungal infections	1890	1970	1998	Malaysia	Immunologic	4
Methyprylon	Sedative-hypnotic	GABA-A receptor modulation	Hypnosedation	1955	1961	1984	Zimbabwe	Drug abuse	4
Metipranolol	Antihypertensive	Non-selective beta blocker	Hypertension	1986	1991	1991	UK	Sensory systems (uveitis)	4
Metofoline	Analgesic	Opioid receptor agonist	Analgesia	1962	1965	1965	USA	Sensory systems	5*
Metrizamide	Radiography		Contrast medium	1977	1980	1992	UK, Netherlands	Nervous system, sensory systems	4

Metrodin	Hormones	GnRH agonist	Invitro fertilization (IVF)	1993	2003	2003	UK	Nervous system	4
Mianserin	Antidepressant	Norepinephrine & serotonin reuptake inhibitor	Depression	1975	1979	1988	Oman	Hematologic (agranulocytosis)	4
Mibefradil	Antihypertensive	Calcium channel blockers	Hypertension	1997	1998	1998	USA; UK; Peru; South Africa; Jamaica; Bulgaria; Armenia	Drug interactions, musculoskeletal	4
Miglustat	Enzyme inhibitor	Glucocerebrosidase	Gaucher's disease type 1	2002	2002	2002	Israel	Unexplained cognitive dysfunction	4
Minaprine	Antidepressant	Reversible inhibitor of MAO-A	Depression	1972	1983	1996	France, Spain, Germany	Drug abuse, nervous system	4
Minocycline	Antimicrobial	Protein synthesis inhibitor	Bacterial infection	1967	1974	1989	Norway	Dizziness, vertigo	4
Molsidomine	Antianginal	Nitric oxide synthesis	Angina pectoris	1972	1975	1985	Germany	Tumorigenicity	5*
Moxisylyte (thymoxamine; uroalpha)	Antianginal	Alpha1-adrenergic antagonist	Benign prostatic hyperplasia	1989	1991	1993	France	Liver	4
Mucopolysaccharide polysulfuric acid ester	Antiarthritic	Endogenous hyaluronate synthesis	Rheumatism	1962	1987	1988	Switzerland, France, Portugal, Austria	Skin: histochemical study	4
MMR vaccine (Urabe)	Vaccines	Antibody production	Mumps virus	1983	1987	1988	Worldwide	Nervous system, psychiatric	2
Muzolimine‡	Antihypertensive	Diuresis	Hypertension	1983	1987	1987	Worldwide	Nervous system	4
Naftidrofuryl oxalate (IV) ‡	Vasodilator	5HT2 receptor antagonist	Intermittent claudication	1974	1976	1992	France; Germany, Spain	Cardiovascular, immunologic, liver, urinary tract	4
Nandrolone decanoate (injectable)	Hormones	Stimulate anabolism & inhibit catabolism	Hypogonadism	1962	1989	1997	France, Bangladesh	Drug abuse	4
Nandrolone phenylpropionate (injectable)	Hormones	Stimulate anabolism & inhibit catabolism	Hypogonadism	1959	1972	1982	Bangladesh	Drug abuse	4
Natalizumab	Monoclonal antibody	Inhibits α4 integrin	Multiple sclerosis & Crohn's disease	2004	2005	2005	USA	Nervous (combination)	2
Nebacumab‡	Monoclonal antibody	Endotoxin-specific IgM	Sepsis	1991	1993	1993	Worldwide	Infection risk; accelerated deaths	2
Nefazodone‡	Antidepressant	5HT2 receptor antagonist	Depression	1994	1999	2003	European Union; Canada; Singapore	Hepatotoxicity	4
Neomycin sulfate (inj)	Antimicrobial	Protein synthesis inhibitor	Bacterial infection	1949	1967	1983	Bangladesh, Philippines, Nigeria, USA; Canada	Drug abuse, sensory systems, urinary tract	4
Nevirapine	Antimicrobial	Non-nucleoside reverse transcriptase inhibitor (NNRTI)	HIV	1996	1997	2007	Brazil	Liver	4

Nialamide	Antidepressant	MAOI	Depression	1959	1969	1974	Japan, India, Cuba, Denmark, Thailand, Venezuela, Canada	Drug interactions, liver	4
Nifedipine (10mg)	Antihypertensive	Calcium channel blockers	Hypertension	1975	1989	1996	Australia	Cardiovascular	4
Nifuroxazide (nifuroxazine)	Antimicrobial	Janus kinase inhibitor	Diarrhea and colitis	1966	1997	2008	Belgium	Immunologic, hematologic	4
Nikethamide	Respiratory stimulant	Unclear	Tranquilizer overdose	1922	1953	1988	Worldwide	Nervous system	4
Nimesulide‡	Analgesic	COX-2 inhibitor	Analgesia	1986	1998	1999	Portugal, Israel, Spain, Bangladesh, Nigeria, India, Ireland, Ghana, Thailand, Vietnam	Liver	4
Niperotidine	Anti-ulcer	H2 receptor antagonist	Peptic ulcer	1990	1995	1995	Italy	Hepatotoxicity	4
Nitrefazole‡	Anti-addiction	Inhibition of aldehyde dehydrogenase	Alcohol deterrent	1982	1984	1984	Germany, Austria	Liver	4
Nitrofurazone (Nitrofural)	Antimicrobial	Unclear	Bacterial infection	1946	1974	1977	Japan, USA, Armenia	Mutagenicity	5*
Nitroxoline	Antimicrobial	↓ in the biofilm density of *P. aeruginosa*	UTI	1965	1966	1973	Ireland, Thailand, Venezuela	Eyes	5*
Nomifensine‡	Antidepressant	Norepinephrine-dopamine reuptake inhibitor	Depression	1976	1980	1986	Worldwide	Hematologic, liver, respiratory	4
Norpseudoephedrine (Phenylpropanolamine)	Psychostimulant	Adrenoreceptor agonist, D1 receptor agonist	Nasal decongestion, obesity	1947	1985	1987	Germany, Brazil, Malaysia, Singapore, USA, Oman, Canada, Cuba, India, Cameroun, Nigeria, Portugal, Temor-Leste, South Korea	Hemorrhagic stroke	4
Noscapine	Antitussive	σ–receptor agonist	Cough	1959	1984	1990	Ireland, UK, Netherlands	Genotoxicity	5*
Novobiocin (cathomycin)	Antimicrobial	Inhibitors of bacterial DNA gyrase	Staphylococcal infections	1956	1957	1987	Malaysia	Hematologic	4
Omeprazole (injectable)	Anti-ulcer	Proton pump inhibitor	Peptic ulcer	1989	1994	1994	Germany	Endocrine, genotoxicity, liver, musculoskeletal, nervous system, sensory systems	4
Opium in antitussives‡	Antitussive	Opioid receptor agonist	Recreation	1812	1842	1982	Bangladesh, Italy	Drug dependence	4
Orciprenaline (metaprotenerol)	Bronchodilator	Beta2 adrenoceptor agonist	Asthma	1961	1972	2009	UK	Cardiovascular	4
Orgotein‡	Antiarthritic	Free radical scavenging	Osteoarthritis	1968	1987	1990	Switzerland, Germany, Portugal	Immunologic	4
Oxeladin	Antitussive	Unclear	Cough	1972	1991	1994	Germany, France, Armenia, Canada	Carcinogenic	5*

Oxolamine	Antitussive	Unclear	Cough	1969	1972	1984	Netherlands	Nervous system: hallucination in children	4
Oxomemazine‡	Antitussive	Histamine and cholinergic receptors' antagonist	Cough	1937	1979	1991	European Union; USA	Respiratory depression in children (SIDS)	4
Oxyphenbutazone‡	Analgesic	COX-1 & COX-2 inhibition	Analgesia	1955	1974	1984	Worldwide	Hematologic: Bone marrow suppression	4
Oxyphenisatin‡	Laxative	↑d mucosal tissue permeability	Constipation	1925	1970	1972	Worldwide	Liver	4
Pangamic acid	"Vitamin"	Transmethylation	Detoxification	1949	1980	1984	Greece	Mutagenic	5*
Paramomycin	Antimicrobial	Protein synthesis inhibitor	Bacterial infection	1959	1970	1989	Spain	Renal damage, neuromuscular blockage and ototoxicity	4
Parecoxib	Analgesic	COX-2 inhibitor	Analgesia	2002	2005	2005	USA, European Union	Cardiovascular, respiratory	2
Pargyline	Antihypertensive	Monoamine oxidase inhibitor	Hypertension	1963	1964	1979	Germany	Interaction with tyramine	4
Pectin	Antidiarrheal	↑d viscosity	Diarrhea	1936	1974	1991	Libya, India, Sri Lanka	Metabolic	4
Pemoline‡	Psychostimulant	Dopamine agonist?	ADHD, narcolepsy	1960	1973	2005	Worldwide	Liver: hepatotoxicity	4
Pentobarbital‡	Sedative-hypnotic	GABA-A receptor modulation	Hypnosedation	1930	1953	1985	Sweden	Drug overdose	4
Pentosan polysulfate sodium	Anti-thrombotic	Protective coating to the damaged bladder wall?	Cystitis, osteoarthritis	1965	1985	1994	France, USA	Hematologic: thrombocytopenia	4
Pentylenetetrazol	Circulatory & respiratory stimulant	Unclear	Convulsion	1934	1954	1982	USA	Convulsion	4
Pergolide Mesylate	Anti-parkinsonian	Dopamine receptor agonist	Parkinson's	2002	2002	2007	USA, Canada	Cardiovascular	3
Perhexiline lameate	Antianginal	Inhibits mitochondrial CPT1	Angina pectoris	1974	1974	1985	UK, Spain, France	Hypoglycemia, liver, musculoskeletal, nervous system	4
Phenacetin	Analgesic	Metabolized to paracetamol	Analgesia	1887	1948	1965	16 European, 14 Asian, 5 African, 4 S. American countries. New Zealand, USA, Canada	Hematologic, liver, tumorigenicity, urinary tract	4
Phenazone (antipyrine)	Analgesic	COX-1 & COX-2 inhibition	Analgesia	1883	1958	1981	UAE, Malaysia, Bahrain, Germany, Iraq	Hematologic	4
Phenazopyridine	Analgesic	Unclear	Urinary analgesia	1927	1978	1984	Greece	Carcinogenic	5*
Phendimetrazine	Psychostimulant	Norepinephrine-dopamine releasing agent	Obesity	1961	1979	1982	Turkey	Drug abuse	4
Phenformin‡	Hypoglycemic	↓d gluconeogenesis	Diabetes	1957	1963	1970	15 European & 6 Asian countries; Canada; New Zealand; Brazil; USA; Ethiopia	Metabolic acidosis	4

Phenicarbazide (phenylsemicarbazide)	Analgesic	Unclear	Analgesia	1948	1950	1979	Ireland	Serious adverse effects: hemolytic anemia	4
Phenmetrazine	Psychostimulant	Norepinephrine-dopamine releasing agent	Obesity	1956	1959	1982	Turkey, Oman, Nigeria	Drug abuse	4
Phenobarbital‡	Sedative-hypnotic	GABA-A receptor modulation	Hypnosedation, convulsion	1912	1929	1985	Sweden, Mauritius, France	Abuse and intoxication; skin	4
Phenol (topical)	Antimicrobial	Absorption by bacteria	Bacterial infection	1867	1973	1983	Dominican Republic, Lithuania	Safer alternatives	4
Phenolphthalein	Laxative	Direct action on intestinal muscles	Constipation	1902	1953	1979	Europe, Yemen, Bangladesh, Canada, France, Morocco, Oman, Japan, Saudi Arabia, Bahrain	Skin, metabolic, muscular	4
Phenoxypropazine	Antidepressant	MAOI	Depression	1961	1964	1966	UK	Drug interactions, liver	4
Phentermine	Psychostimulant	TAAR1 agonist	Obesity	1959	1964	1981	Sweden, UAE, Mauritius, Turkey, Oman, UK, Venezuela	Drug abuse	4
Phentolamine mesilate	Antihypertensive	Alpha-adrenergic antagonist	Erectile dysfunction	1998	1998	2000	Singapore	Carcinogenicity	5*
Phenylbutazone‡	Analgesic	COX-1 & COX-2 inhibition	Analgesia	1949	1953	1985	6 European, 6 Asian & 3 African countries; Chile; Panama	Hematologic, liver, urinary tract	4
Phenylephrine	Decongestant	α1-adrenergic receptor agonist	Mydriasis	1949	1968	1987	UK	Eyes	4
Phthalylsulfathiazole	Antimicrobial	Inhibits dihydropteroate synthetase	Bacterial infection	1946	1950	1982	Bangladesh	Granulocytopenia	4
Pifoxime	Analgesic	COX-1 & COX-2 inhibition	Analgesia	1975	1975	1976	France	Nervous system, psychiatric	4
Pioglitazone	Hypoglycemic	PPARs activation	Diabetes	1999	2011	2011	Europe	Risk of bladder CA	3
Pipamazine	Emetic	Phenothiazine analogue	Emesis	1959	1960	1969	USA	Lack of efficacy	5
Pipenzolate	Anti-ulcer	Muscarinic receptor antagonist	Peptic ulcer	1960	1990	1990	Pakistan	Overdose potential	4
Piperazine	Antihelminth	GABA receptor inhibition	Worm infestation	1949	1957	1983	Malaysia, Armenia	Immunologic, nervous system, tumorigenicity	4
Pipradrol	Psychostimulant	Norepinephrine-dopamine reuptake inhibitor	Obesity, narcolepsy, ADHD	1953	1968	1982	USA, Turkey, Denmark, Venezuela	Nervous, cardiovascular	4
Pirprofen‡	Analgesic	COX-1 & COX-2 inhibition	Analgesia	1982	1986	1990	Worldwide	Gastrointestinal, liver, urinary tract	4
Pituitary chorionic gonadotropin (injectable)	Hormones	Ovulation trigger	Infertility	1930	1958	1972	USA	Immunologic	4

Podophyllum resin	Laxative	Mitotic spindle poison	Constipation	1844	1962	1970	Italy, France, Egypt	Teratogenicity	4
Polidexide	Antilipemic	Anion exchange	Hyperlipidemia	1974	1977	1977	UK	Oculomucocutaneous syndrome	4
Polyoxyethylated castor oil	Adjuvant		Emulsifier	1970	1973	1984	Worldwide	Anaphylaxis, hyperlipidemia	4
Polyvinylpyrrolidone (Polyvidone; povidone)	Adjuvant	Permeability through circulatory system	Suspending and dispersing of IV drugs	1957	1967	1983	Germany, Pakistan, Egypt, USA	Metabolic: granulomatous lesions	4
Potassium arsenate	Tonic	Degradation of the aberrant retinoic acid receptor α fusion protein	Leukemia; Psoriasis	1878	1968	1980	USA	Tumorigenicity	4
Potassium canrenoate	Antihypertensive	Aldosterone antagonist	Hypertension, ascites	1968	1985	1986	Germany	Carcinogenic	5*
Potassium chloride	Electrolyte	Replenishment of potassium stores	Hypokalemia	1962	1964	1989	Belgium, France, USA, Canada	Gastrointestinal perforation	4
Potassium nitrate	Antihypertensive	Nitric oxide synthesis	Hypertension	1901	1975	1981	France, Egypt, Venezuela	Tumorigenicity	3
Practolol‡	Antihypertensive	Beta-blocker	Hypertension	1970	1972	1975	Greece, Turkey, New Zealand, Denmark, Thailand, Singapore, UK, Mauritius, India, Germany, Norway, Venezuela	Gastrointestinal, sensory systems, skin	4
Pramipexole dihydrochloride monohydrate	Antiparkinsonian	Dopamine receptor agonist	Parkinson's	1997	1999	2006	Europe	Sudden onset of sleep	3
Prenylamine‡	Antianginal	Calcium channel blocker	Angina pectoris	1960	1973	1989	Worldwide	Cardiovascular: multifocal ventricular tachycardia	4
Probucol	Antioxidant	Unclear	Hyperlipidemia	1980	1989	1989	Germany, France, USA	Cardiovascular: Torsade de pointes	4
Proglumide	Anti-ulcer	Cholecystokinin antagonist	Peptic ulcer	1970	1984	1989	Japan, Germany	Respiratory	3
Pronethalol	Antihypertensive	Beta-blockade	Angina pectoris	1963	1963	1965	UK	Tumorigenicity	5*
Propanidid	Anesthetic	Unclear	General anesthesia	1963	1965	1983	UK, Norway, France	Immunologic reactions	4
Propofol (children) ‡	Anesthetic	GABA-A receptor agonist, sodium channel blockade	Anesthesia	1987	1992	1992	Israel, Norway, UK, Lithuania	Metabolic, hepatic, nervous; deaths	4
Propyphenazone‡	Analgesic	COX-1 & COX-2 inhibition	Analgesia	1951	1980	1989	Turkey, UAE, Bahrain, Ireland	Hematologic, Immunologic: Lyell's syndrome	4
Proxibarbal	Sedative-hypnotic	GABA-A receptor modulation	Hypnosedation	1956	1990	1998	France, Italy, Spain, Portugal and Turkey	Hematologic, immunologic	4
Pseudoephedrine	Sympathomimetic	Direct action on adrenergic receptors	Nasal decongestion	1959	1963	2008	Iraq, Colombia, Thailand	Nervous, GIT, immunologic, cardiovascular, teratogenic	3

Pumactant‡	Surfactant	Lowers lung surface tension	Respiratory distress	1992	2000	2000	UK	"Accelerated deaths"	2
Purified hexavalent vaccine (Hexavac)	Vaccines	Immunogenicity	Bacterial and viral infections	2000	2005	2005	Europe	Decreased immunogenicity	2
Pyrithyldione/diphenhydramine	Sedative-hypnotic	Similar to barbiturates	Insomnia	1940	1949	1997	Spain	Hematologic: agranulocytosis	4
Pyritinol	Nootropic	Increased cerebrovascular blood flow	Dementia syndromes	1961	1973	1982	Bangladesh	Insufficient therapeutic value and risk of misuse	4
Pyrovalerone	Psychostimulant	Norepinephrine-dopamine reuptake inhibitor	Obesity, chronic fatigue syndrome	1974	1975	1979	France	Drug abuse	4
Pyrrolizidine	Antitussive	Coating effect	Cough, sinusitis	1967	1968	1992	Germany, Belgium, UK	Liver	5*
Rapacuronium bromide‡	Anesthetic	Non-depolarizing neuromuscular blocker	Anesthesia	1999	1999	2001	USA	Respiratory: Bronchospasm	3
Remoxipride	Antipsychotic	D2 receptor antagonist	Psychoses, schizophrenia	1991	1993	1994	Worldwide	Hematologic	4
Rimonabant	Psychostimulant	Cannabinoid receptor agonist	Obesity	2006	2006	2007	Europe, India	Psychiatric	1
Rofecoxib‡	Analgesic	COX-2 inhibitor	Analgesia, osteoarthritis	1999	2002	2004	Worldwide	Cardiotoxicity	1
Rosiglitazone‡	Hypoglycemic	↓d gluconeogenesis	Diabetes	1999	2007	2011	UK; New Zealand; South Africa, India	Cardiovascular	1
Rotavirus vaccine	Vaccines	Immunogenicity	Rotavirus	1998	1999	1999	USA, UK	Gastrointestinal: intussusception	3
Santonin‡	Antihelminth	Paralysis of nerve impulses	Ascariasis	1911	1933	1978	Singapore	Neurotoxicity	4
Sargramostim	Immunostimulator	Granulocyte macrophage colony-stimulating factor (GM-CSF)	Bone marrow transplantation	1993	1999	2008	USA	Immune-mediated	4
Secobarbital/ quinalbarbital	Sedative-hypnotic	GABA-A receptor modulation	Hypnosedation, convulsion	1934	1958	1990	France, Norway, Netherlands, Ghana, New Zealand, Oman	Drug abuse	4
Selegiline	Antiparkinsonian	MAO-B inhibitor	Parkinson's, depression, dementia	1982	1991	1997	USA	Drug interaction	4
Sertindole‡	Antipsychotic	5HT & D2 receptor antagonist	Psychoses	1996	1996	1998	UK, Bulgaria, Spain	Cardiovascular, death	2
Sibutramine‡	Psychostimulant	Serotonin-norepinephrine reuptake inhibitor	Obesity	2001	2002	2002	European Union; 4 Asian countries; Australia; Canada; Mexico; New Zealand; USA	Cardiovascular	4
Sitaxentan sodium‡	Antihypertensive	Endothelin receptor antagonist	Pulmonary arterial hypertension	2006	2009	2010	Worldwide	Hepatotoxicity	4

Sodium dibunate (ethyl dibunate)	Antitussive	Unclear	Cough	1963	1968	1982	Philippines	Nervous	5*
Sodium hydrogen bicarbonate (pediatric)	Antacid	Counteracts acidity	Indigestion	1966	1983	1997	Nepal	Safety concerns: metabolic	4
Somatropin (growth hormone) ‡	Hormones	Binds to the human growth hormone receptor	Hypopituitary dwarfism	1973	1985	1985	Europe; Egypt; New Zealand; USA; Oman; Thailand	Infection risk: Creutzfeldt-Jakob disease	4
Soruvidine‡	Antimicrobial	Inhibits DNA polymerase	Herpes viral infection	1993	1993	1993	Germany; Japan	Drug interactions with 5-fluorouracil	4
Sparfloxacin	Antimicrobial	Inhibits DNA gyrase	Bacterial infection	1993	1995	1995	Europe, USA	QTc prolongation, phototoxicity	4
Sparteine sulphate	Hormone analogue	Prostaglandin stimulation?	Labour	1939	1963	1979	USA	Uterine tetany	4
Strychnine and salts	Aphrodisiac	Acetylcholine receptor antagonist	Impotence	1753	1882	1979	Canada, Brazil, Bangladesh, Japan, Pakistan, UAE, Philippines	Nervous	4
Sulfacarbamide (Sulfanilylurea)	Antimicrobial	Inhibits dihydropteroate synthetase	Bacterial infection	1946	1959	1992	Germany	Hematologic, liver, skin, urinary tract	4
Sulfadicramide (sulfacetamide)	Antimicrobial	Inhibits dihydropteroate synthetase	Bacterial infection	1942	1976	1992	Germany	Negative benefit-to-harm balance: eye, skin, immune	4
Sulfadimethoxine‡	Antimicrobial	Inhibits dihydropteroate synthetase	Bacterial infection	1958	1961	1966	USA	Skin	4
Sulfadimidine	Antimicrobial	Inhibits dihydropteroate synthetase	Bacterial infection	1942	1954	1992	USA; Germany; Armenia	Hematologic: thrombocytopenia	4
Sulfaguanidine	Antimicrobial	Inhibits dihydropteroate synthetase	Bacterial infection	1941	1969	1971	Dominican Republic, Iran, Turkey, Pakistan, Nepal, Germany, UAE, Denmark, Venezuela	Hematologic: agranulocytosis	4
Sulfamerazine sodium	Antimicrobial	Inhibits dihydropteroate synthetase	Bacterial infection	1943	1955	1992	Germany	Hematologic; skin	4
Sulfamethizole	Antimicrobial	Inhibits dihydropteroate synthetase	Bacterial infection	1953	1963	1984	Sweden	Immunologic	4
Sulfamethoxydiazine	Antimicrobial	Inhibits dihydropteroate synthetase	Bacterial infection	1962	1968	1988	Germany	Skin	4
Sulfamethoxypyridazine	Antimicrobial	Inhibits dihydropteroate synthetase	Bacterial infection	1957	1958	1984	Sweden, Pakistan, UAE, UK, Canada, Argentina, France	Hematologic, skin	4
Sulfanilamide	Antimicrobial	Inhibits dihydropteroate synthetase	Bacterial infection	1936	1957	1992	Germany	Sensory, Immunologic	4

Sulfathiazole	Antimicrobial	Inhibits dihydropteroate synthetase	Bacterial infection	1941	1954	1970	USA, Philippines, Dominican Republic, France, UAE, India	Hematologic, liver, skin, urinary tract	4
Sulfisomidine	Antimicrobial	Inhibits dihydropteroate synthetase	Bacterial infection	1952	1972	1992	Germany	Negative benefit-to-harm balance	4
Sulfonamides (topical)	Antimicrobial	Inhibits dihydropteroate synthetase	Bacterial infection	1930	1957	1986	Chile	Immune-mediated	4
Suloctidil‡	Vasodilator	Calcium channel blockade, inhibits thromboxane B2	Intermittent claudication	1975	1983	1985	Worldwide	Liver: hepatotoxicity	4
Suprifen with tussilax	Antitussive		Cough	1971	1971	1972	France, Germany	Liver: hepatotoxicity	3
Suprofen	Analgesic	COX-1 & COX-2 inhibition	Analgesia	1983	1986	1986	Worldwide	Urinary tract	4
Suxibuzone	Analgesic	COX-1 & COX-2 inhibition	Analgesia	1974	1982	1986	Oman, Italy	Carcinogenesis	5*
Technetium (^{99m}Tc) fanolesomab‡	Radiography	Radioisotope	Radiographic imaging	2004	2005	2005	USA	Cardiovascular	4
Tegaserod	Antispasmodic	5-HT4 receptor agonist	Irritable bowel syndrome, constipation	2002	2004	2007	USA, Canada, India, Jordan, Australia, Switzerland, China, Argentina	Cardiovascular	1
Temafloxacin‡	Antimicrobial	Inhibits DNA gyrase	Bacterial infection	1991	1992	1992	Worldwide	Hematologic, immunologic, liver, metabolism, urinary tract	4
Temazepam (gel capsules) ‡	Sedative-hypnotic	GABA-A receptor binding	Hypnosedation	1969	1981	1999	Australia	Drug abuse	3
Terconazole	Antimicrobial	Disrupts cell membrane permeability by cytochrome P450 14-alpha-demethylase	Fungal infection	1980	1988	1991	Sweden, Germany	Immunologic, skin	4
Terfenadine‡	Antihistamine	H1-receptor antagonist	Allergy	1985	1985	1997	3 European, 2 African, 3 Asian & 3 South American countries; USA, Canada	Cardiovascular, immunologic, liver, skin	4
Terodiline‡	Antispasmodic	Calcium channel blockade, blocks cholinergic receptor	Urinary incontinence	1965	1989	1991	Worldwide	Cardiovascular	4
Testosterone propionate (injectable)	Hormones	Androgen-receptor modulation	Hypogonadism, sexual dysfunction	1937	1952	1982	Bangladesh	Drug abuse	4
Tetrabamate	Anxiolytic	Febarbamate, difebarbamate plus phenobarbital	Alcohol dependence	1981	1992	2001	France, Spain	Liver, skin	4
Tetrachlorosalicylanilide	Antimicrobial	Leakage of cell contents	Bacterial infection	1949	1961	1975	USA, Japan	Skin	4

Tetracycline (pediatric)	Antimicrobial	Protein synthesis inhibitor	Bacterial infection	1948	1959	1975	8 Asian, 2 African, 2 Australasian countries; USA, Italy	Teeth; bone	3
Tetrazepam	Sedative-hypnotic	Benzodiazepine site agonist	Hypnosedation	1960	1988	2013	EU	Dermatitis	4
Thalidomide	Immunomodulator	? CNS mechanism	Hyperemesis	1957	1961	1961	Belgium, Finland, Indonesia, Canada, Brazil, Denmark, India, Singapore, Venezuela, UK, Germany	Nervous system, teratogenicity	4
Thenalidine tartrate‡	Antihistamine	H1-receptor blocker	Allergy	1953	1958	1958	USA; UK; Sweden; France; Cyprus; Australia; Finland; Norway, Canada	Hematologic: neutropenia	4
Thiobutabarbital	Sedative-hypnotic	GABA-A receptor modulation	Hypnosedation	1939	1981	1993	Germany	Urinary tract	5*
Thioridazine	Antipsychotic	5HT2 receptor antagonist	Psychoses	1959	1961	2000	Worldwide	Retinopathy, cardiovascular	4
Thorium dioxide	Radiography	Radiocontrast	Diagnostic imaging	1928	1931	1955	UK	Tumorigenicity	4
Tick-borne encephalitis vaccine	Vaccines	Immunogenicity	Viral infection	1999	1999	2000	Germany	Hyperpyrexia	2
Tienilic acid (ticrynafen) ‡	Antihypertensive	Loop diuretic	Hypertension, kidney stones	1976	1979	1980	Greece; Philippines; USA; Brazil; Germany; Panama; France; India; Venezuela	Liver, urinary tract	4
Tilbroquinol	Antiprotozoal	?	Diarrhea (amoebiasis)	1969	1996	1997	France, Morocco, Saudi Arabia, Switzerland	Liver, nervous system	4
Timonacic acid (thioproline)	Antioxidant	Cysteine release and restoration of glutathione concentrations	Hepatitis	1975	1981	1982	France	Drug overdose (toxicity)	3
Tocainide‡	Antiarrhythmic	Sodium channel blockade	Cardiac arrhythmia	1981	1986	1986	USA	Hematologic: agranulocytosis, aplastic anemia	4
Tolcapone‡	Antiparkinsonian	COMT inhibitor	Parkinson's	1998	1998	1998	UK; Australia; Ireland; Spain; Portugal; Lithuania; Bulgaria; Canada	Liver	4
Tolrestat‡	Hypoglycemic	Aldase reductase inhibitor	Diabetes	1982	1995	1996	Worldwide	Liver	2
Tranylcypromine	Antidepressant	MAOI	Depression	1961	1963	1964	Italy, Belgium, Venezuela, Canada	Cardiovascular, drug interactions	4
Trazodone	Antidepressant	Binds at 5-HT2 receptor	Depression	1973	1983	1985	Norway	Carcinogenic	5*
Tretinoin	Retinoid	Modulates cell-cycle progression, cellular differentiation, cell survival and apoptosis	Acne	1973	1977	1988	Germany	Carcinogenic	5*
Triacetyldiphenolisatin	Laxative	↑d mucosal tissue permeability	Constipation	1961	1967	1971	Australia, Germany, Italy, Canada, Cyprus, New Zealand, Venezuela	Photosensitivity; hepatotoxicity	4
Triazolam	Sedative-hypnotic	GABA-A receptor binding	Hypnosedation	1978	1978	1979	Mauritius, Europe, Brazil, Oman	Nervous system, psychiatric	4

Trimethobenzamide (suppository)	Antihistamine	?Chemoreceptor trigger zone	Motion sickness, GI infections, medication-induced nausea	1959	1976	2007	USA	Due to new FDA regulation; lack of effectiveness. Teratogenicity	3
Triparanol	Antilipemic	δ 24-reductase inhibitor	Hyperlipidemia	1959	1961	1962	USA, France	Sensory systems, skin	4
Troglitazone‡	Hypoglycemic	PPARs activation	Diabetes	1997	1997	1997	UK; Switzerland; Peru; Jamaica; Chile, USA, Canada	Liver	4
Trovafloxacin‡	Antimicrobial	Inhibits DNA gyrase	Bacterial infection	1997	1997	1999	European Union; Philippines; Syria; Vietnam; Singapore, Canada, USA	Liver	4
Urethane	Solvent	?Interferes with pyrimidine metabolism	Cancer (CML)	1933	1943	1963	Brazil, Cuba, Denmark, Egypt, Japan, Thailand, USA, Canada, Italy, Greece, Denmark, Venezuela, France	Hematologic, liver, tumorigenicity	5*
Valdecoxib	Analgesic	COX-2 inhibitor	Analgesia	2001	2003	2005	USA, Canada, Europe, India	Cardiovascular; skin	2
Veralipride	Neuroleptic	Selective antagonism of hypothalamic D2 receptors	Climacteric	1979	1982	2007	Europe, Brazil (reference to EMA)	Psychiatric	4
Vigabatrin (gamma-vinyl-GABA)	Antiepileptic	Inhibits GABA transaminase (GABA-T)	Hypnosedation	1989	1989	1991	Norway	Not medically justified; neurotoxicity	5*
Vinbarbital‡	Sedative-hypnotic	GABA-A receptor modulation	Hypnosedation	1939	1975	1984	Sweden	Drug abuse	4
Vincamine	Nootropic	Unclear	Meniere's disease, vertigo	1955	1974	1980	Hungary, Germany	Cardiovascular, hematologic	4
Vitamin B complex (injectable)	Hematinic	Co-factor for biochemical transformation	Anemia	1949	1953	2008	Iraq	Immunologic	4
Vitamin E (injectable) ‡	Antioxidant	Downregulation of ICAM-1 and VCAM-1	Retinal deterioration in neonates	1983	1984	1984	USA	Death, hematologic, liver, urinary tract	3
Xenazoic acid (xenalamine)	Antimicrobial	? Inhibits intracellular stages viral biological cycle	Viral infection	1960	1965	1965	Belgium, France, Venezuela	Liver	4
Ximelagatran	Anticoagulant	Direct thrombin inhibitor	DVT, stroke	2005	2006	2006	Europe, Brazil	Liver	2
Zimeldine	Antidepressant	5 HT reuptake inhibitor	Depression	1982	1982	1983	Worldwide	Liver, nervous system	4
Zipeprol HCl‡	Antitussive	Laryngeal nerve stimulation; excitation of tracheo-bronchial receptors	Cough	1971	1984	1991	Philippines, Brazil, Spain, Switzerland, Turkey	Drug abuse, drug dependence, nervous system	4 & 5
Zomepirac‡	Analgesic	COX-1 & COX-2 inhibition	Analgesia	1979	1981	1983	Worldwide	Immunologic, urinary tract	4
Zopiclone	Sedative-hypnotic	GABA-A receptor binding	Hypnosedation	1985	1985	1986	Israel, Norway	Carcinogenicity	5*

ABBREVIATIONS: ADHD: Attention deficit hyperactivity disorder; COMT: Catechol-O-methyl transferase; COX-1: Cyclooxygenase 1; COX-2: Cyclooxygenase 2; CPT1: Carnitine palmitoyltransferase I; CTZ: Chemoreceptor trigger zone; DNA: Deoxyribonucleic acid; D1: Dopamine 1; D2: Dopamine 2; D3: Dopamine 3; DVT: Deep venous thrombosis; GABA: Gamma-aminobutyric acid; HMG CoA: 3-hydroxy-3-methyl-glutaryl-CoA; hURAT1: Human uric acid transporter 1; H1: Histamine 1;

ICAM-1: Intercellular Adhesion Molecule 1; MAOI: Monoamine oxidase inhibitor; NMDA: N-methyl-D-aspartate; PPAR: Peroxisome proliferator-activated receptors; SSRI: Selective serotonin reuptake inhibitor; TAAR 1: Trace amine-associated receptor 1; VCAM-1: Vascular cell adhesion protein 1; 5 HT: 5-hydroxytryptamine

[Δ]Re-prohibited again in 1986 following FDA legal action (http://www.atsdr.cdc.gov/toxprofiles/tp64.pdf)
†Based on the Oxford Centre for Evidence-Based Medicine Levels of Evidence [13]. Level 1: Systematic review of randomized trials, systematic review of nested case-control studies, Level 2: Individual randomized trial or (exceptionally) observational study with dramatic effect; Level 3: Non-randomized controlled cohort/follow-up study (post-marketing surveillance); Level 4: Case-series, case-control, or historically controlled studies; Level 5: Mechanism-based reasoning
*First withdrawal based on evidence from animal research
‡Products were withdrawn in association with deaths
§The first reported adverse reaction appeared after first withdrawal from the market
