# Supplementary material for: Correction to: Post-marketing withdrawal of 462 medicinal products because of adverse drug reactions: a systematic review of the world literature
Source: BMC Med. 2019 Mar 2;17:56. doi: 10.1186/s12916-019-1294-9 (PMC6397477; doi:10.1186/s12916-019-1294-9)
Supplement: Supplementary file 1 — E-appendix Table 1. List of medicinal products withdrawn because of adverse drug reactions. (DOCX 157 kb) [file 12916_2019_1294_MOESM1_ESM.docx]

**E-appendix Table 1: List of medicinal products withdrawn because of adverse drug reactions.**

| **Medicinal product** | **Class** | **Mechanism of action** | **Therapeutic indication** | **Launch date** | **Year of first ADR report** | **Year first withdrawn** | **Countries withdrawn** | **Reason for withdrawal** | **Level of evidence†** |
| --- | --- | --- | --- | --- | --- | --- | --- | --- | --- |
| Acetanilide | Analgesic | Metabolised to paracetamol | Analgesia | 1886 | 1940 | 1971 | Japan | Aplastic anemia | 4 |
| Acetarsol | Antimicrobial | Unknown | Syphilis, trichomoniasis | 1921 | 1941 | 1982 | Mauritius | Hematologic | 4 |
| Acetylfuratrizine | Antimicrobial | Damages ribosomal proteins | Bacterial infection | 1962 | 1977 | 1977 | Japan, Saudi Arabia, Venezuela | Superseded by safer products: carcinogenic | 5* |
| Acitretin | Retinoid | Binds to the retinoic acid receptor (RAR) | Psoriasis | 1989 | 1989 | 1990 | Netherlands, France | Teratogenicity | 5* |
| Acridine derivatives | Antimicrobial |  | Topical disinfectant | 1922 | 1975 | 1979 | Denmark, Venezuela | Mutagenic | 5* |
| Adenosine phosphate | Antiarrhythmic | Direct nodal inhibition | Cardiac arrhythmia | 1930 | 1957 | 1973 | USA | Cardiovascular | 4 |
| Alatrofloxacin | Antimicrobial | Inhibit DNA gyrase | Bacterial infection | 1997 | 1998 | 2000 | Armenia, Singapore, Europe | Liver | 4 |
| Alclofenac | Analgesic | Inhibit COX-1 & COX-2 | Analgesia | 1972 | 1974 | 1977 | Ireland, Cyprus, Germany, UK, Italy, New Zealand, Egypt, Greece, Denmark, Indonesia, India, Jordan, Morocco | Immunologic, liver, skin, urinary tract | 4 |
| Aliskiren | Antihypertensive | Direct renin inhibitor | Hypertension | 2007 | 2011 | 2011 | Europe | Angioedema | 4 |
| Alosetron‡ | Antispasmodic | 5HT3 receptor antagonist | Irritable bowel syndrome | 2000 | 2000 | 2000 | USA | Gastrointestinal | 4 |
| Alphacetylmethadol | Analgesic | OP1 receptor agonist | Analgesia | 1993 | 2001 | 2003 | USA | Cardiovascular | 4 |
| Alpidem‡ | Anxiolytic | GABA-A receptor antagonist | Anxiety | 1991 | 1994 | 1994 | France | Liver | 4 |
| Althesin (alphadolone/alphaxolone) | Sedative-hypnotic | Potentiation of the GABA-A chloride channel | Anesthesia | 1972 | 1973 | 1984 | UK, Germany, France | Immunologic | 4 |
| Amfepramone | Psychostimulant | Norepinephrine releasing agent | Obesity | 1957 | 1974 | 1975 | Turkey, Sweden, Oman, UAE, Norway, Venezuela, EU, France, UK | Cardiotoxicity | 4 |
| Amfetamine | Psychostimulant | TAAR1 agonist | Obesity | 1939 | 1957 | 1973 | USA, UAE, Turkey, Oman, Malaysia, Nigeria | Drug dependence | 4 |
| Amineptine | Antidepressant | Dopamine receptor antagonist | Depression | 1960 | 1984 | 1999 | France, Thailand, UAE, Bangladesh, Morocco, Vietnam | Drug dependence, liver, skin | 4 |
| Aminoglutethimide | Anxiolytic | Steroid synthesis inhibitor | Anxiety | 1960 | 1966 | 1966 | US, Saudi Arabia, Norway | Endocrine | 4 |
| Aminophenazone ‡ (aminopyrine) | Analgesic | Inhibit COX-1 & COX-2 | Analgesia | 1887 | 1936 | 1965 | 10 European, 14 Asian, & 4 African countries; Australia; USA; Canada; Chile; Brazil; Venezuela | Hematologic, carcinogenic | 4 |
| Aminophylline | Anti-asthmatic | Nonselective phosphodiesterase inhibitor | Asthma | 1908 | 1955 | 1992 | Netherlands | Immune-mediated reactions | 4 |
| Aminorex fumarate | Psychostimulant | Catecholamine release | Obesity | 1962 | 1967 | 1967 | Germany, Venezuela | Respiratory | 4 |
| Amobarbital‡ | Sedative-hypnotic | GABA-A receptor agonist | Hypnosedation | 1961 | 1979 | 1985 | Sweden, New Zealand | Respiratory depression | 4 |
| Amoproxan | Antianginal | Nonselective β-blocker | Angina | 1969 | 1970 | 1970 | France | Sensory systems, skin | 4 |
| Anabolic steroids | Hormones | Androgen-receptor modulation | Aplastic anemia, breast cancer, osteoporosis | 1952 | 1966 | 1989 | Thailand, India | Androgenic effects | 4 |
| Anagestone acetate | Hormones | Inhibition of ovulation | Contraception | 1968 | 1969 | 1969 | Worldwide | Tumorigenicity | 5* |
| Anti-D immunoglobulin | Immune prophylaxis | Binds to the erythrocyte D antigen | Idiopathic thrombocytopenic purpura | 1968 | 2000 | 2009 | Europe | Safety concerns: hemoglobinemia, | 4 |
| Anti-histamine (topical) | Phenothiazine derivatives | H1-antagonism | Hypersensitivity | 1942 | 1947 | 1986 | Malaysia, Sri Lanka | Immune-mediated reactions | 4 |
| Antrafenine‡ | Analgesic | COX-1 & COX-2 inhibition | Analgesia | 1977 | 1979 | 1984 | France | Urinary tract | 4 |
| Aprobarbital‡ | Sedative-hypnotic | GABA-A receptor receptor modulation | Hypnosedation | 1920 | 1966 | 1985 | Sweden | Respiratory depression | 4 |
| Aprotinin | Antifibrinolytic | Pancreatic trypsin inhibitor | Bleeding | 1959 | 1964 | 2007 | Worldwide | Anaphylaxis | 4 |
| Arsenate | Chemotherapeutic agent | Oncoprotein expression | Cancer | 1951 | 1973 | 1988 | Austria, Spain, Italy, France, Philippines | Drug abuse, carcinogenic | 3 |
| Aspirin | Analgesic | COX-1 & COX-2 inhibition | Analgesia | 1899 | 1970 | 1986 | UK, US, Hong Kong, Nigeria, Spain | Liver | 3 |
| Astemizole | Antihistamine | H1-receptor antagonist | Allergy | 1977 | 1986 | 1987 | Norway, New Zealand, Philippines, USA, Canada, South Africa, UAE, Mauritius, Brunei, Tanzania, Armenia, Brazil, Spain, Argentina, Singapore, India, Chile | Cardiovascular, drug interactions | 4 |
| Azaribine‡ | Antipsoriatic | Inhibition of orotidine-5'- monophosphate decarboxylase | Psoriasis | 1975 | 1976 | 1976 | USA; Thailand; Mauritius; Saudi Arabia; Venezuela | Hematologic, psychiatric | 4 |
| Barbital | Sedative-hypnotic | GABA-A receptor modulation | Hypnosedation | 1903 | 1913 | 1971 | Italy | Respiratory depression | 4 |
| Beclobrate‡ | Antilipemic | Activates PPAR | Hyperlipidemia | 1985 | 1990 | 1990 | Switzerland | Hepatotoxicity | 4 |
| Bendazac | Analgesic | COX-1 & COX-2 inhibition | Analgesia | 1983 | 1987 | 1993 | Spain | Liver | 4 |
| Benfluorex‡ | Psychostimulant | Fenfluramine analogue | Obesity | 1976 | 2003 | 2009 | Europe | Cardiotoxicity | 3 |
| Benoxaprofen‡ | Analgesic | COX-1 & COX-2 inhibition | Analgesia | 1980 | 1982 | 1982 | Worldwide | Liver, skin, urinary tract | 4 |
| Benzarone‡ | Thrombolytic | Antagonistic on mediators of muscle contraction, direct action on smooth muscle cells | Varicose veins | 1964 | 1987 | 1992 | Germany, Portugal, France | Liver | 4 |
| Benzbromarone | Uricosuric agent | Xanthine oxidase inhibitor | Gout | 1976 | 1994 | 2003 | Portugal, France | Liver | 4 |
| Benziodarone | Uricosuric agent | Inhibits hURAT1 | Gout | 1962 | 1964 | 1964 | UK, France, Spain | Liver | 4 |
| Benzydamine (Difflam) | Analgesic | COX-1 & COX-2 inhibition | Analgesia | 1967 | 1980 | 1995 | Germany | Skin, psychiatric, sensory | 4 |
| Benzyl alcohol‡ | Antimicrobial | Alteration of bacterial membrane permeability | Decontamination of IV lines in neonates | 1972 | 1981 | 1982 | Israel, USA, Oman, Iraq | Metabolic acidosis | 4 |
| Benzylpenicillin sodium (topical preparations) | Antimicrobial | Inhibits cell wall biosynthesis | Bacterial infection | 1944 | 1953 | 1972 | USA, Italy, Philippines, Ethiopia, Bangladesh, India, Chile, Cyprus, Spain, Thailand, Venezuela | Skin, immune | 4 |
| Bepridil‡ | Antiarrhythmic | Calcium channel blockers | Cardiac arrhythmia | 1981 | 1982 | 2004 | USA | Cardiovascular | 4 |
| Beta-ethoxyacetanilide | Analgesic | Metabolized to paracetamol? | Analgesia | 1886 | 1978 | 1986 | Germany | Tumorigenicity, urinary tract | 5* |
| Bezitramide‡ | Analgesic | Unknown | Analgesia | 1961 | 1983 | 2004 | Netherlands | Overdose | 4 |
| Bicalutamide‡ | Chemotherapeutic agent | Androgen receptor modulation | Prostate cancer | 1995 | 2001 | 2003 | Canada, UK | Accelerated deaths | 2 |
| Bismuth salts‡ | Antidyspepsia | Unclear. Forms insoluble complexes | Dyspepsia | 1875 | 1930 | 1978 | France; Egypt; Japan; Greece; Austria; Bangladesh; Turkey; Mauritius; Oman; Cuba; India | Nervous, cardiovascular | 4 |
| Bithionol | Antihelminth | Exact mechanism unclear | Worm infestation | 1958 | 1960 | 1967 | USA, Canada, Japan | Skin | 4 |
| Boric acid and borates‡ | Antimicrobial | Destruction or inhibition of growth of pathogenic organisms | Skin rash | 1875 | 1945 | 1990 | Ireland, Malaysia, Korea, Philippines, Thailand, Germany, Japan, Costa Rica, UK, India, Peru, USA | Deaths from neurotoxicity | 4 |
| Bovine tissue-derived medicines‡ | Various: heparin, glucagon, insulin, blood factors | Various | Various | 1982 | 1986 | 1989 | Ireland, Switzerland, France | Neurotoxicity | 4 |
| Bromfenac‡ | Analgesic | COX-1 & COX-2 inhibition | Analgesia | 1997 | 1998 | 1998 | USA, Saudi Arabia | Liver | 4 |
| Bromisoval | Sedative-hypnotic | Similar to barbiturates | Hypnosedation | 1909 | 1930 | 1987 | Netherlands | Drug dependence | 4 |
| Bromocriptine mesylate‡ | Anti-lactation | D2 and D3 agonist | Lactation prevention | 1976 | 1983 | 1989 | USA | Cardiovascular, drug interactions, nervous system, pregnancy, psychiatric | 4 |
| Brotizolam | Sedative-hypnotic | GABA-A receptor modulation | Hypnosedation | 1982 | 1983 | 1989 | UK | Tumorigenicity | 5* |
| Broxyquinoline | Antiprotozoal | Production of free radicals | Amoebiasis | 1960 | 1968 | 1970 | Japan, UAE, Saudi Arabia | Sensory | 4 |
| Bucetin | Analgesic | Phenacetin analogue | Analgesia | 1968 | 1985 | 1986 | Germany | Urinary tract: Mutagenic, carcinogenic | 5* |
| Budipine | Antiparkinsonian | Muscarinic & NMDA receptor antagonist | Parkinson's | 1979 | 2000 | 2000 | Germany | Cardiovascular | 4 |
| Bufexamac | Analgesic | COX-1 & COX-2 inhibition | Analgesia | 1973 | 1973 | 1990 | France | Skin | 4 |
| Buflomedil‡ | Vasodilator | α-adrenergic blockade | Peripheral arterial occlusive disease | 1970 | 1981 | 2006 | France, Europe | Neurotoxicity; cardiotoxicity | 4 |
| Buformin‡ | Hypoglycemic | Reduce gluconeogenesis | Diabetes | 1950 | 1969 | 1978 | Germany, Austria, Belgium, Ireland | Metabolism | 4 |
| Bumadizone injection | Analgesic | COX-1 & COX-2 inhibition | Rheumatism | 1972 | 1978 | 1986 | Oman | Hematologic | 5* |
| Bunamiodyl‡ | Radiography | Selective secretion in bile | Radiography | 1958 | 1962 | 1964 | USA, Sweden, Venezuela | Kidney | 4 |
| Buprenorphine‡ | Analgesic | Agonist–antagonist opioid receptor modulator | Analgesia | 1978 | 1983 | 1986 | Egypt | Fatalities (IV use) | 4 |
| Bupropion | Antidepressant | Norepinephrine-dopamine reuptake inhibitor | Depression | 1985 | 1985 | 1986 | USA | Nervous system | 4 |
| Butamben | Anesthetic | ↓ neuronal membrane permeability to sodium ions | Local anesthesia | 1923 | 1947 | 1964 | UK | Allergic, psychiatric, skin | 4 |
| Cadralazine | Antihypertensive | Peripheral arteriolar vasodilator | Hypertension | 1989 | 1991 | 1992 | Norway | Immunologic | 2 |
| Camazepam | Sedative-hypnotic | GABA-A receptor modulation | Hypnosedation | 1978 | 1984 | 1984 | Netherlands | Immunologic | 4 |
| Canrenone | Antihypertensive | Diuresis, aldosterone antagonist | Aldosteronism, CHF, hypertension | 1966 | 1976 | 1986 | Germany | Tumorigenicity | 5* |
| Carbinoxamine | Antihistamine | Competes with free histamine for binding at HA-receptor sites | Allergy | 1953 | 1987 | 2008 | Iraq | Neurotoxicity | 4 |
| Carisoprodol | Muscle relaxant | Unknown | Sprain, muscle injury | 1959 | 2002 | 2007 | Sweden, EU, Indonesia | Abuse | 4 |
| Cartilage + bone marrow | Antiarthritic |  | Degenerative joint disease | 1960 | 1989 | 1992 | Germany | Skin | 3 |
| Catechic extract‡ | Antiinflammatory | Unclear | Benign prostatic hyperplasia | 1972 | 1979 | 1982 | France | Hematologic | 4 |
| Cefaloridine | Antimicrobial | Disruption of cell wall synthesis | Bacterial infection | 1964 | 1969 | 1989 | Spain | Nephrotoxic | 4 |
| Celecoxib (Onsenal) ‡ | Analgesic | COX-2 inhibitor | Familial adenomatous polyposis | 2003 | 2006 | 2011 | Europe | Increased risk of serious cardiovascular events | 3 |
| Cell preparations‡ | Antiageing |  | Adjuvant; antiaging | 1931 | 1957 | 1987 | Austria, Germany, Switzerland | Immunologic | 4 |
| Cerivastatin‡ | Antilipemic | HMG CoA reductase inhibition | Hyperlipidemia | 1997 | 1998 | 2001 | Worldwide | Renal, musculoskeletal | 4 |
| Chenodeoxycholic acid | Antilipemic | ↓ cholesterol saturation of bile | Cholelithiasis (gallstones) | 1975 | 1976 | 1987 | Norway | Tumorigenicity | 5* |
| Chloral hydrate | Sedative-hypnotic | GABA receptor modulation | Hypnosedation | 1869 | 1986 | 2001 | USA, France, India | Tumorigenicity | 5* |
| Chloramphenicol | Antimicrobial | Disruption of cell wall synthesis | Bacterial infection | 1947 | 1950 | 1978 | France, Spain, Hungary, India | Hematologic | 4 |
| Chlormadinone acetate | Hormones | Inhibition of ovulation | Contraception | 1965 | 1970 | 1972 | USA, UK, Italy, Egypt, Venezuela | Tumorigenicity | 5* |
| Chlormezanone | Muscle relaxant | GABA receptor modulation | Anxiolytic, muscle sprain & injury | 1960 | 1983 | 1996 | Worldwide | Skin, drug dependence, liver | 4 |
| Chlornaphazine | Chemotherapeutic agent | Cell hydrolysis | Polycythemia & Hodgkin's disease | 1964 | 1964 | 1964 | Denmark, Venezuela | Tumorigenicity | 4 |
| Chloroform (trichloromethane) | Anesthetic | Depression of the respiratory centres | Anesthesia | 1847 | 1976 | 1976 | Greece, Turkey, Japan, USA, Panama, Saudi Arabia, Brazil, Italy, Canada, Norway, Philippines, UK, Denmark, NZL, Ethiopia, Zimbabwe, Bangladesh, Dominican Republic, Belgium, Nigeria, Ireland, Oman, Cuba, India | Cardiovascular, liver, tumorigenicity, urinary tract | 5* |
| Chloroquine | Antiprotozoal | Cell lysis | Malaria | 1939 | 1959 | 1975 | Japan, Guinea Bissau | Eyes | 4 |
| Chlorphentermine | Psychostimulant | TAAR1 agonist | Obesity | 1962 | 1970§ | 1969 | Germany, Venezuela | Respiratory, cardiovascular | 5* |
| Chorionic gonadotrophin | Hormones | Spermatogenesis | Hypogonadism | 1934 | 1961 | 1972 | USA | Immunologic | 4 |
| Cianidanol‡ | Antioxidant | Free radical scavenger | Hepatitis | 1976 | 1980 | 1985 | Worldwide | Hematologic | 4 |
| Cincophen | Analgesic | Adrenal stimulation | Gout | 1910 | 1923 | 1991 | Germany, Italy | Liver | 4 |
| Cinepazide | Vasodilator | Phosphodiesterase inhibition | Cerebrovascular disease | 1974 | 1985 | 1988 | Egypt, Spain | Hematologic | 4 |
| Cisapride monohydrate‡ | Prokinetic agent | 5-HT4 receptor agonist | Gastro-esophageal reflux | 1993 | 1993 | 2000 | Philippines, Oman, USA, Germany, UK, Serbia, Mauritius, Canada, Brunei, Turkey, Indonesia, Singapore, Japan, Cuba, Bahrain, Australia, Armenia, India | Cardiovascular, drug interactions | 4 |
| Cliobutinol | Antitussive | Unclear | Cough | 1961 | 2004 | 2007 | Europe, Argentina | Cardiovascular | 4 |
| Clioquinol | Antimicrobial | Inhibition of DNA replication | Diarrhea | 1934 | 1964 | 1970 | Japan, Norway, Sweden, Denmark, UAE, Nigeria, Bangladesh, Philippines, Italy, Nepal, Dominican Republic, Zimbabwe, Spain, Hong Kong, Ethiopia, Honduras, Oman, Pakistan, Ghana, Libya, Bahrain, Netherlands, Saudi Arabia, Canada | Nervous system | 4 |
| Clobenzorex | Psychostimulant | Similar to amphetamine | Obesity | 1966 | 1986 | 2000 | Mauritius, USA | Drug abuse, psychiatric | 4 |
| Clofenotane | Pesticide | Interfere with voltage-gated sodium channel | Pediculosis | 1945 | 1969 | 1972 | USA | Carcinogenicity | 5* |
| Clofibrate‡ | Antilipemic | Activates PPAR | Hyperlipidemia | 1967 | 1973 | 1978 | Germany, Israel, Norway, Bangladesh | Accelerated deaths | 4 |
| Cloforex | Psychostimulant | Similar to amphetamine | Obesity | 1965 | 1967 | 1967 | Germany, Sweden, Venezuela | Cardiovascular, drug abuse, psychiatric | 4 |
| Clomacron | Analgesic | COX-1 & COX-2 inhibition | Analgesia | 1966 | 1970 | 1982 | UK | Liver | 4 |
| Clometacin‡ | Analgesic | COX-1 & COX-2 inhibition | Analgesia | 1971 | 1981 | 1990 | France | Liver, skin, urinary tract | 4 |
| Clozapine | Antipsychotic | Unknown | Schizophrenia | 1972 | 1975 | 1975 | Finland, Singapore, Norway | Cardiovascular, hematologic | 4 |
| Cobalt | Hematinic | As cobalamin | Iron-deficiency anemia | 1951 | 1954 | 1967 | USA, Kuwait | Cardiovascular, liver | 4 |
| Codeine | Analgesic | G-protein receptor modulation | Analgesia | 1832 | 1951 | 1985 | Bangladesh, Malaysia | Abuse | 4 |
| Co-proxamol (paracetamol + dextropropoxyphene) ‡ | Analgesic | ?COX-3 inhibition; agonist at mu opioid receptors | Analgesia | 1957 | 1960 | 2007 | Europe, India, USA, Zimbabwe, New Zealand, Iraq | Overdose; respiratory depression; cardio and hepatotoxicity | 4 |
| Coumarin (synthetic) ‡ | Anticoagulant | Vitamin K reductase inhibition | Lymphedema post-radiation | 1996 | 1996 | 1996 | France, Australia | Liver | 4 |
| Cupric bisquinoline | Antiarthritic | Antiinflammatory with penicillamine complex | Rheumatism | 1958 | 1967 | 1978 | France | Neuromuscular | 5* |
| Cyclandelate | Vasodilator | Direct effect on vascular smooth muscle | Raynaud's disease | 1987 | 1989 | 1996 | USA | Not effective for use | 4 |
| Cyclobarbital | Sedative-hypnotic | GABA-A receptor receptor agonist | Hypnosedation | 1962 | 1974 | 1980 | Norway, France | Drug dependence, drug overdose | 4 |
| Cyclofenil | Anti-hormone | Estrogen receptor modulation | Scleroderma | 1970 | 1980 | 1987 | France | Liver | 3 |
| Cyclovalone + retinol + tiratricol‡ | Choleretic | ? | Hyperlipidemia, dyspepsia, obesity | 1964 | 1984 | 1988 | France | Liver | 4 |
| Cyproheptadine | Antihistamine | Antihistamine, anticholinergic | Allergy, anorexia | 1961 | 1977 | 1982 | Bangladesh, Malaysia, UK | Drug abuse | 4 |
| Dalkon shield | Hormones | Prevention of fertilization | Contraception (IUD) | 1971 | 1974 | 1974 | USA, UK, New Zealand | Septic abortions | 4 |
| Danthron ( chrysazin) | Laxative | ? | Constipation | 1959 | 1985 | 1987 | Norway, Germany, Japan, USA, Singapore, Canada | Tumorigenicity | 5* |
| Datura | Analgesic | Muscarinic receptor antagonist (atropine) | Asthma | 1920 | 1962 | 1992 | France | Drug abuse | 4 |
| Dequalinium chloride | Lozenges | ? | Disinfectant | 1956 | 1970 | 1984 | Greece | Low efficacy, skin reactions | 4 |
| Desensitizing vaccines | Vaccines | ? | Viral infection | 1973 | 1985 | 1989 | UK | Immunologic | 4 |
| Dexamfetamine | Psychostimulant | Adrenergic receptor agonist | Narcolepsy, ADHD | 1937 | 1966 | 1973 | USA, Turkey, Oman, Nigeria | Drug abuse and dependence | 4 |
| Dexfenfluramine | Psychostimulant | Serotonin receptor agonist | Obesity | 1995 | 1995 | 1997 | USA, Canada, Morocco, Philippines, Lithuania, India | Cardiovascular, respiratory | 4 |
| Diacetoxydiphenolisatin | Laxative | Phenacetin analogue | Diarrhea | 1971 | 1971 | 1971 | Australia | Liver | 4 |
| Diamthazole | Antimicrobial | ? | Fungal infection | 1951 | 1953 | 1972 | US, France | Nervous system, psychiatric | 4 |
| Dibenzepine hydrochloride‡ | Antidepressant | Norepinephrine reuptake inhibitor | Depression | 1963 | 1967 | 1983 | Sweden | Suicides | 4 |
| Dibromsalan | Antimicrobial | ? | Bacterial infection | 1962 | 1966 | 1975 | USA, Japan | Skin | 4 |
| Diclofenac sodium | Analgesic | COX-1 & COX-2 inhibition | Analgesia | 1979 | 1979 | 1983 | Philippines, Norway | Hematologic, tumorigenicity | 4 |
| Dicycloverine | Antispasmodic | Muscarinic anticholinergic | Irritable bowel syndrome | 1952 | 1974 | 1986 | Bangladesh, Norway | Nervous, psychiatric | 4 |
| Dienestrol | Hormones | Estrogen receptor agonist | Hormone replacement therapy | 1947 | 1972 | 1977 | Austria, Italy, Kuwait, Saudi Arabia | Carcinogenicity | 3 |
| Diethyl-aminoethoxyhexestrol | Antianginal | ? | Angina pectoris | 1964 | 1970 | 1970 | Japan | Liver | 3 |
| Diethylstilbestrol | Hormones | Uterine desensitization, local arteriolar constriction | Threatened abortion | 1938 | 1971 | 1973 | Panama, Austria, Kuwait, Italy | Tumorigenicity | 3 |
| Difemerine | Antispasmodic | Muscarinic antagonist | GI spasms | 1967 | 1980 | 1986 | Germany, France, USA | Nervous system | 4 |
| Difenoxin | Antispasmodic | Opioid receptor agonist | Diarrhea | 1970 | 1979 | 1991 | Pakistan, Oman, South Korea, Lebanon | Atropism | 2 |
| Difurazone | Antimicrobial | ? | Bacterial infection | 1966 |  | 1977 | Japan, Saudi Arabia, Venezuela | Superseded by safer products | 5 |
| Dihydrostreptomycin | Antimicrobial | Protein synthesis inhibitor | Bacterial infection | 1947 | 1949 | 1970 | USA, Philippines, Spain, Dominican Republic, Italy, Peru | Nervous system, psychiatric, sensory systems | 3 |
| Dihydroxy-methylfuratrizine | Antimicrobial | Nitrofuran analogue | Bacterial infection | 1975 | 1975 | 1977 | Japan, Saudi Arabia, Venezuela | Tumorigenic | 5* |
| Diiododiethyltin‡ | Antimicrobial | ? | Bacterial infection | 1954 | 1955 | 1957 | France | Nervous system | 4 |
| Dilevalol | Antihypertensive | Nonselective beta blocker; beta-2 receptor agonist | Hypertension | 1989 | 1989 | 1990 | Worldwide | Liver | 4 |
| Dinitrophenol | Organic compound | Uncoupling of oxidative phosphorylation | Obesity | 1933 | 1933 | 1938^∆^ | USA | Cataracts, agranulocytosis | 4 |
| Dinoprostone | Prostaglandin | Direct vasodilator | Induction of labour | 1971 | 1987 | 1990 | UK | Fetal distress; uterine hypertonia | 3 |
| Diphenazine (quietidin) | Analgesic | ? | Analgesia | 1962 | 1967 | 1967 | Hungary, Venezuela | Photosensitivity | 4 |
| Diphenoxylate | Antispasmodic | Opioid receptor agonist | Diarrhea | 1956 | 1969 | 1990 | Libya, Pakistan, Mexico, Nepal, Philippines, South Korea, Lebanon, Thailand | Nervous system | 4 |
| Dithiazanine iodide‡ | Antihelminth | Interruption of glucose uptake in cells | Worm infestation | 1959 | 1960 | 1964 | USA; France; Chad; Italy; Cuba | Cardiovascular, metabolism | 4 |
| Dofetilide | Antiarrhythmic | Potassium channel blocker | Cardiac arrhythmia | 1999 | 1999 | 2004 | Europe | Cardiovascular | 2 |
| Dolansetron | Propulsive | 5-HT3 receptor antagonist | Emesis | 1997 | 2005 | 2011 | Germany | Cardiovascular | 4 |
| Domperidone (injectable) ‡ | Propulsive | Dopamine receptor antagonist | Emesis, GERD | 1979 | 1982 | 1985 | Worldwide | Cardiovascular, drug overdose, endocrine, nervous system | 4 |
| Doxycycline (IV) ‡ | Antimicrobial | Protein synthesis inhibitor | Bacterial infection | 1959 | 1984 | 1989 | France, Morocco | Anaphylaxis | 4 |
| Doxylamine/dicyclomine | Antihistamine | H1-receptor blocker | Allergy, hyperemesis | 1956 | 1981 | 1983 | UK, USA | Teratogenicity | 3 |
| Drotrecogin alfa (activated) | Antithrombotic | Unclear | Sepsis | 2001 | 2001 | 2011 | Worldwide | Insufficient evidence; bleeding risk | 1 |
| Droperidol | Antipsychotic | Dopamine 2 receptor antagonist | Psychoses | 1970 | 1991 | 2001 | UK, Indonesia | Cardiovascular; deaths | 4 |
| Droxicam | Analgesic | COX-1 & COX-2 inhibition | Analgesia | 1990 | 1992 | 1994 | Europe | Liver | 4 |
| Ebrotidine‡ | Anti-ulcer | H2 receptor antagonist | Peptic ulcer | 1997 | 1998 | 1998 | Spain, Peru | Liver | 4 |
| Efalizumab | Monoclonal antibody | Inhibits CD11a | Autoimmune disease | 2003 | 2008 | 2009 | EU, USA, Canada, Mexico | Leukoencephalopathy | 4 |
| Emetin (ipecac syrup) | Emetic | Stimulation of the CTZ, local irritation | Emesis | 1912 | 1947 | 1982 | Mauritius | Cardiotoxicity | 3 |
| Encainide‡ | Antiarrhythmic | Na channel blocker | Cardiac arrhythmia | 1985 | 1989 | 1991 | UK | Cardiovascular | 4 |
| Epinephrine (topical) | Anesthetic | Vasoconstriction | Local anesthesia | 1899 | 1963 | 2004 | Ireland, Venezuela | Cardiovascular, nervous | 4 |
| Erythrityl tetranitrate | Antihypertensive | Nitric oxide synthesis | Angina pectoris | 1955 | 1987 | 1998 | USA | Insufficient evidence: skin | 4 |
| Erythromycin balleul | Antimicrobial | Protein synthesis inhibitor | Bacterial infection | 1955 | 1975 | 1995 | France | Teratogenic | 4 |
| Erythromycin estolate | Antimicrobial | Protein synthesis inhibitor | Bacterial infection | 1958 | 1961 | 1976 | Singapore, Greece, Sudan, Mauritius, Bangladesh, Bahrain, Denmark, Sweden | Liver | 4 |
| Ethanol | Antimicrobial | GABA-A receptor modulation, protein coagulation | Sedation, disinfectant | 1850 | 1946 | 1985 | Chile, Sri Lanka, Brazil, UAE | Neurotoxicity, abuse | 4 |
| Ethchlorvynil | Sedative-hypnotic | Unknown | Hypnosedation | 1955 | 1959 | 1978 | USA | Drug dependence, respiratory | 4 |
| Ethinyl estradiol | Hormones | ?Gonadotropin suppression | Contraception | 1943 | 1963 | 2004 | Germany, France | Venous thrombosis | 3 |
| Ethyl nitrite (spirit) ‡ | Antimicrobial | Increased GFR, nitric oxide synthesis | Diaphoresis, GI spasm | 1850 | 1977 | 1980 | USA | Methemoglobinemia | 4 |
| Ethylene dichloride (topical) | Antimicrobial |  | Bacterial infection | 1794 | 1977 | 1978 | Germany, Saudi Arabia | Carcinogenic | 5* |
| Ethylestrenol | Hormones | Stimulate anabolism & inhibit catabolism | Testosterone deficiency | 1964 | 1965 | 1982 | Bangladesh | Inappropriate use in malnourished children | 4 |
| Etomidate | Anesthetic | GABA-A receptor modulation | Anesthesia | 1972 | 1983 | 1985 | UK | Endocrine | 3 |
| Etretinate | Retinoid | Inhibits cell differentiation & hyperkeratinization | Psoriasis | 1981 | 1982 | 1992 | Norway, Brazil, France, USA, Canada | Teratogenicity, tumorigenicity, death | 5* |
| Exifone | Psychostimulant | Free radical scavenger? | Memory deficits | 1988 | 1989 | 1989 | France | Liver | 4 |
| Factor IX | Anti-hemophilic | Hydrolysis of disulfide bonds | Hemophilia | 1952 | 1986 | 1986 | Sweden | HIV | 4 |
| Factor VIII | Anti-hemophilic | Cofactor for factor IXa | Hemophilia | 1965 | 1986 | 1986 | UK | HIV | 4 |
| Febarbamate | Sedative-hypnotic | GABA-A receptor receptor modulation | Hypnosedation | 1967 | 1991 | 1997 | France | Liver | 4 |
| Felbamate‡ | Anti-epileptic | NMDA receptor antagonist | Epilepsy | 1993 | 1993 | 1994 | USA, European Union | Hematologic, liver | 4 |
| Fenbutrazate | Psychostimulant | Phenmetrazine analogue | Obesity | 1957 | 1963 | 1969 | Europe | Drug abuse, psychiatric | 2 |
| Fenclofenac‡ | Analgesic | COX-1 & COX-2 inhibition | Analgesia | 1978 | 1984 | 1984 | Worldwide | Skin, tumorigenicity, urinary tract | 4 |
| Fenclozic acid | Analgesic | COX-1 & COX-2 inhibition | Analgesia | 1969 | 1970 | 1970 | UK, USA | Liver | 2 |
| Fenetylline | Psychostimulant | Amphetamine + theophylline properties | ADHD, narcolepsy | 1961 | 1962 | 1991 | Oman, Bulgaria | Abuse | 4 |
| Fenfluramine | Psychostimulant | Serotonin receptor antagonist | Obesity | 1973 | 1981 | 1997 | Worldwide | Cardiovascular, respiratory | 3 |
| Fenoterol‡ | Antiasthmatic | Beta 2 adrenergic agonist | Asthma | 1971 | 1987 | 1990 | New Zealand, Australia | Death | 3 |
| Fenproporex | Psychostimulant | Amphetamine analogue | Obesity | 1966 | 1997 | 1999 | Europe | Drug abuse, psychiatric | 4 |
| Fentanyl hydrochloride‡ | Anagesic | μ-opioid receptor agonist | Analgesia, anesthesia | 2006 | 2006 | 2009 | Europe | Risk of overdose | 4 |
| Feprazone | Analgesic | COX-1 & COX-2 inhibition | Analgesia | 1978 | 1981 | 1984 | UK, Germany, Greece, Egypt | Liver, gastrointestinal, skin, urinary tract | 4 |
| Fipexide‡ | Nootropic | TAAR1 receptor modulation? | Memory deficits | 1973 | 1990 | 1991 | France | Hematologic, liver | 4 |
| Floctafenine | Analgesic | COX-1 & COX-2 inhibition | Analgesia | 1976 | 1976 | 1987 | Belgium | Dermatologic | 4 |
| Flosequinan‡ | Vasodilator | Direct relaxing effects | Congestive heart failure | 1992 | 1992 | 1993 | UK, USA | Death | 2 |
| Flosulide | Analgesic | COX-2 inhibitor | Analgesia | 1994 | 1995 | 1996 | Germany | Kidneys | 5* |
| Flunitrazepam | Sedative-hypnotic | GABA-A receptor modulation | Hypnosedation | 1974 | 1979 | 1986 | South Africa, Sweden | Drug abuse | 4 |
| Fluproquazone | Analgesic | COX-1 & COX-2 inhibition | Analgesia | 1979 | 1984 | 1989 | USA | Liver | 4 |
| Fluvoxamine | Antidepressant | Selective serotonin re-uptake inhibitor | Obsessive Compulsive disorder; depression | 1984 | 1987 | 1987 | Iceland | Teratogenicity, renotoxicity | 5* |
| Furazolidone | Antimicrobial | Disruption of bacterial DNA | Bacterial infection | 1954 | 1965 | 1977 | Japan, Iraq, Malaysia, South Korea, Lebanon, Yemen, India | Skin, hematologic, nervous, respiratory, sensory | 4 |
| Gadofosveset trisodium | Radiography | Binds to human serum albumin | Diagnostic imaging | 1988 | 2006 | 2010 | European Union | Kidney | 3 |
| Gallopamil | Antiarrhythmic | Calcium channel blockers | Cardiac arrhythmia | 1983 | 1985 | 2001 | Turkey | Not specified: Cardiovascular | 2 |
| Ganglioside (bovine) | Glycolipids | Dimerization of neurotrophic factor tyrosine kinases | Peripheral neuropathies | 1975 | 1985 | 1989 | Germany | Nervous system | 3 & 5 |
| Gatifloxacin | Antimicrobial | Inhibition of DNA gyrase | Bacterial infection | 1999 | 2002 | 2011 | Europe, India | Dysglycemia | 3 |
| Gelatin | Coagulant | ? | Bleeding | 1915 | 1978 | 1978 | USA | Hematologic | 4 |
| Gemfibrozil | Anticholesterol | Activates peroxisome proliferator-activated receptor-alpha (PPARα) | Dyslipidemia | 1982 | 1982 | 1987 | Norway | Negative benefit-to-harm balance | 2 |
| Gemtuzumab ozogamicin‡ | Monoclonal antibody | Binds to the CD33 antigen | Leukemia | 2000 | 2010 | 2010 | USA | Accelerated deaths | 2 |
| Genaconazole | Antimicrobial | Interferes with the fungal synthesis of ergosterol | Fungal infection | 1970 | 1992 | 1992 | USA | Hepatocellular cancer | 5* |
| Gentamicin (topical) | Antimicrobial | Interruption of protein synthesis | Bacterial infection | 1966 | 1976 | 1994 | Netherlands, UAE | Resistance | 3 |
| Glafenine | Analgesic | COX-1 & COX-2 inhibition | Analgesia | 1965 | 1973 | 1984 | Worldwide | Urinary tract | 4 |
| Glucosamine sulphate (injection) | Antiarthritic | Precursor in biochemical synthesis of glycosylated proteins and lipids | Rheumatism | 1980 | 1981 | 1986 | Germany, Egypt | Immune: hypersensitivity | 3 |
| Glutethimide | Sedative-hypnotic | Similar to barbiturates | Hypnosedation | 1955 | 1962 | 1980 | Norway, Zimbabwe, Pakistan, France | Drug abuse, drug dependence, sensory systems | 4 |
| Glycosaminoglycan | Antiarthritic | ? | Rheumatism | 1975 | 1982 | 1982 | Germany | Hematologic | 3 |
| Grepafloxacin‡ | Antimicrobial | Inhibition of DNA gyrase | Bacterial infection | 1997 | 1999 | 1999 | Worldwide | Cardiovascular | 4 |
| Guanethidine | Antihypertensive | Sympathetic antagonist | Hypertension | 1973 | 1978 | 1986 | UK | Sensory systems | 4 |
| Guanofuracin | Antimicrobial | Disruption of bacterial DNA | Bacterial infection | 1966 |  | 1977 | Japan, Venezuela | Safer alternatives | 5 |
| HA-1A (Centoxin) | Monoclonal antibody | Endotoxin lipopolysaccharide | Sepsis | 1991 | 1991 | 1993 | US | Death | 2 |
| Halogenated hydroxyquinoline derivatives | Antimicrobial | Production of free radicals | Amoebic dysentery | 1949 | 1971 | 1978 | Denmark, Cyprus, Philippines, Bangladesh, Ghana, Turkey, Italy, Greece, Oman, UAE, India | Nervous | 4 |
| Halogenated salicylanilides | Antimicrobial | Production of free radicals | Bacterial infection | 1954 | 1964 | 1975 | USA, Japan | Skin, eyes | 4 |
| Heptabarb‡ | Sedative-hypnotic | GABA-A receptor modulation | Convulsion | 1956 | 1984 | 1984 | Sweden | Drug abuse | 4 |
| Herpes simplex vaccines | Vaccines | Herpes simplex antibodies | Viral infection (HSV) | 1964 |  | 1984 | Germany, Saudi Arabia, Venezuela | Potential hazards | 5 |
| Hexachlorophene | Antimicrobial | Production of free radicals | Bacterial infection | 1948 | 1968 | 1972 | Germany, Japan, Turkey, Europe, USSR, Peru | Encephalopathy, mutagenicity, teratogenicity | 3 |
| Hexestrol | Chemotherapeutic agent | Nonsteroidal estrogen | Prostate CA | 1946 | 1977 | 1977 | Austria, Italy, Kuwait, Armenia, Saudi Arabia, Venezuela | Carcinogenic | 4 |
| Hexestrol bis (beta-diethylaminoethyl ether) | Vasodilator | Coronary vasodilatation | Hypertension | 1952 | 1963 | 1969 | Japan | Hepatotoxicity | 4 |
| Hexobarbital‡ | Sedative-hypnotic | GABA-A receptor modulation | Convulsion | 1945 | 1962 | 1984 | Sweden | Drug abuse | 4 |
| Human placental extract | Placenta derived tissue | ? | Arthritis, eczema, acne vulgaris | 1956 | 1988 | 1994 | Germany, UK, India | Neurotoxicity; Immune; skin | 4 |
| Hydrochlorothiazide + sotalol | Antihypertensive | Diuresis plus beta receptor antagonist | Hypertension | 1970 | 1979 | 1986 | France | Cardiovascular, drug interactions | 4 |
| Hydromorphone | Analgesic | Opioid receptor agonist | Analgesia | 1926 | 1985 | 2005 | USA, Switzerland | Overdose potential | 4 |
| Hyoscine methonitrate | Antispasmodic | Muscarinic receptor antagonist | GI spasms | 1947 | 1964 | 1981 | Sweden | Drug abuse | 4 |
| Ibufenac | Analgesic | COX-1 & COX-2 inhibition | Analgesia | 1961 | 1964 | 1968 | UK | Liver | 4 |
| Indalpine | Antidepressant | SSRI | Depression | 1983 | 1985 | 1985 | France | Gastrointestinal, hematologic | 4 |
| Indometacin (Osmosin) ‡ | Analgesic | COX-1 & COX-2 inhibition | Analgesia | 1963 | 1978 | 1983 | UK | Gastrointestinal; deaths | 4 |
| Indoprofen‡ | Analgesic | COX-1 & COX-2 inhibition | Analgesia | 1976 | 1982 | 1983 | Worldwide | Carcinogenic | 4 |
| Indoramin | Vasodilator | Alpha-1 adrenoceptor antagonist | BPH, Hypertension | 1981 | 1986 | 2011 | UK | Cardiovascular | 4 |
| Influenza virus, split, inactivated (pandremix) | Vaccines | Antibody production | Viral infection (influenza) | 2006 | 2010 | 2010 | Europe | Narcolepsy | 3 |
| Insulin, inhaled (Exubera) | Hormones | Decreased gluconeogenesis | Diabetes | 2006 | 2008 | 2008 | USA, Europe | Lung cancer | 2 |
| Interferon-gamma-1b‡ | Immunomodulator | Binds directly to the type II interferon gamma receptor IFNGR1 | Idiopathic pulmonary fibrosis | 2002 | 2007 | 2007 | USA | Accelerated deaths | 2 |
| Iodinated casein | Hormones | Thyroxine analogue | Obesity | 1944 | 1964 | 1964 | USA | Endocrine, metabolism | 4 |
| Iophendylate | Radiography |  | Radiocontrast imaging | 1944 | 1945 | 1987 | UK | Nervous system | 4 |
| Iproniazid | Antidepressant | MAOI | Depression | 1952 | 1958 | 1959 | Italy, Canada | Immunologic, liver | 4 |
| Isaxonine phosphate‡ | Neurotrophic | Muscle reinnervation | Peripheral neuropathies | 1981 | 1983 | 1984 | France, Tunisia | Hematologic, liver | 4 |
| Isocarboxazid | Antidepressant | MAOI | Depression | 1959 | 1966 | 1974 | Japan, Cuba, Venezuela | Metabolic | 4 |
| Isoprenaline | Cardiac stimulant | Non-selective beta-adrenergic agonist | Bradycardia & heart block, asthma | 1949 | 1968 | 1992 | Sri Lanka, UK, Australia, New Zealand | Cardiovascular | 3 |
| Isotretinoin | Retinoid | Unknown; apoptosis | Cystic acne | 1982 | 1983 | 1988 | Denmark, Austria, Germany, Portugal, Norway, | Teratogenicity | 3 |
| Isoxicam‡ | Analgesic | COX-1 & COX-2 inhibition | Analgesia | 1983 | 1985 | 1985 | Worldwide | Skin | 4 |
| Kaolin | Antidiarrheal |  | Diarrhea | 1954 | 1968 | 1991 | India, Sri Lanka | Metabolism | 4 |
| Ketoconazole‡ | Antimicrobial | Inhibits the cytochrome P450 14α-demethylase | Fungal infection | 1976 | 1981 | 2013 | EU, Australia | Hepatotoxicity | 1 |
| Ketoprofen (gel) | Analgesic | COX-1 & COX-2 inhibition | Analgesia | 1980 | 1983 | 2008 | Egypt, France | Immunologic | 4 |
| Ketorolac (inj) ‡ | Analgesic | COX-1 & COX-2 inhibition | Analgesia | 1989 | 1992 | 1992 | Germany, France, Jamaica | Gastrointestinal, skin | 4 |
| Lapdap (chlorproguanil + dapsone) | Antimalarial | Inhibits dihydrofolate reductase | Malaria | 2003 | 2008 | 2008 | Worldwide | Hemolytic anemia | 2 |
| Laropiprant / nicotinic acid | Antilipemic | Prostaglandin receptor blocker | Facial flushing | 2008 | 2008 | 2008 | Worldwide | Higher frequency of non-fatal but serious side effects | 2 |
| Lead oxide and lead salts (topical) | Astrigent |  | Skin tightening | 1892 | 1977 | 1980 | France, Denmark, Saudi Arabia, Venezuela | Nervous (encephalopathy) | 4 |
| Levacetylmethadol | Antidote | mu-opioid receptor agonist, nicotinic acetylcholine receptor antagonist | Opioid dependence | 1995 | 2001 | 2001 | Europe, USA | Cardiovascular | 4 |
| Levamfetamine | Psychostimulant | Amphetamine analogue | Obesity | 1944 | 1954 | 1973 | USA, Oman, UAE | Drug abuse and dependence | 4 |
| Levamisole‡ | Antihelminth | Neuromuscular depolarizing blockade | Worm infestation | 1966 | 1976 | 1999 | US, Canada, Vietnam | Hematologic, nervous system | 3 |
| Levarterenol | Vasopressor | L-norepinephrine analogue | Nonhemorrhagic shock | 1904 | 1958 | 1973 | Ireland, Venezuela | Nervous, cardiovascular | 4 |
| Lindane | Insecticide | CNS stimulation | Head lice | 1942 | 1972 | 2001 | Brazil | Toxicity | 4 |
| Letrozole | Hormone antagonist | Non-steroidal aromatase inhibitor | Hormonally-responsive breast cancer | 2001 | 2005 | 2011 | India | Teratogenic | 3 |
| Loperamide (syrup & drops) ‡ | Antidiarrheal | Opioid receptor agonist | Diarrhea | 1975 | 1990 | 1990 | Worldwide | Paralytic ileus | 4 |
| Loxoprofen sodium‡ | Analgesic | COX-1 & COX-2 inhibition | Analgesia | 1983 | 1993 | 2000 | Singapore | Colonic ulceration | 4 |
| L-Tryptophan‡ | Antidepressant | Converted into serotonin | Depression and sleep disorders | 1963 | 1989 | 1989 | Worldwide | Eosinophilia-myalgia syndrome (EMS) | 4 |
| Lumiracoxib‡ | Analgesic | COX-2 inhibitor | Analgesia | 2006 | 2007 | 2007 | Australia, USA, Canada, Europe, New Zealand, Brazil, Philippines, Colombia | Liver | 4 |
| Lyme disease vaccine | Vaccines | Immunogenicity | Bacterial infection (Lyme disease) | 1998 | 2001 | 2002 | USA | Musculoskeletal (arthralgia) | 3 |
| Lynestrenol | Hormones | Inhibits gonadotropin, suppression of follicle maturation | Contraception | 1962 | 1970 | 1980 | Australia | Tumorigenicity | 5* |
| Lysine amidotriazoate | Radiography |  | Vascular diagnostics | 1975 | 1981 | 1995 | Germany | Cardiovascular, hematologic, immunologic, urinary tract (safer alternatives) | 5* |
| Mazindol | Psychostimulant | Norepinephrine & dopamine reuptake inhibitor | Obesity | 1970 | 1980 | 1987 | Oman | Drug abuse, psychiatric (interaction with lithium) | 4 |
| Mebanazine | Antidepressant | MAOI | Depression | 1963 | 1964 | 1975 | UK | Drug interactions, liver | 4 |
| Meclozine (meclizine) | Antihistamine | H1 receptor antagonist | Motion sickness | 1953 | 1963 | 1963 | Indonesia | Teratogenic potential | 5* |
| Medifoxamine | Antidepressant | Dopamine reuptake inhibitor; 5HT-receptor agonist | Depression | 1983 | 1991 | 1999 | Morocco, France | Liver | 4 |
| Mefenorex | Psychostimulant | Amphetamine analogue | Obesity | 1966 | 1995 | 1999 | Europe | Drug abuse, psychiatric | 4 |
| Megestrol acetate | Hormones | Progesterone receptor agonist | Contraception | 1963 | 1975 | 1976 | Greece, Norway, Germany, New Zealand | Tumorigenicity | 5* |
| Mepacrine (quinacrine) | Antimicrobial | Unclear | Non-surgical sterilization | 1935 | 1953 | 1998 | India | Carcinogenicity; ectopic pregnancy; possibly political | 2 |
| Mepazine | Antiepileptic | Unclear | Epilepsy | 1955 | 1957 | 1970 | USA | Cardiovascular, gastrointestinal, hematologic, liver, urinary tract | 4 |
| Mephenesin | Muscle relaxant | Spinal reflex inhibition | Sedation, muscle relaxation | 1948 | 1955 | 1976 | Japan, Saudi Arabia | Cardiotoxicity | 4 |
| Meprobamate | Muscle relaxant | GABA-A receptor modulation | Anxiety | 1955 | 1957 | 1981 | Sweden, Europe | Drug abuse | 4 |
| Mercurothiolate (thiomersal) | Antimicrobial | Organomercury analogue | Bacterial and fungal infections | 1928 | 1996 | 1999 | UK, Malaysia, Brazil | Nervous system, tumorigenicity, urinary tract | 4 |
| Mercury amide | Antimicrobial | Organomercury analogue | Disinfectant | 1956 | 1964 | 1969 | Japan, Brazil, Philippines, France, Nigeria, Ghana, Italy, Canada | Nervous system | 4 |
| Mercurous chloride | Antimicrobial | Organomercury analogue | Teething | 1796 | 1949 | 1953 | Worldwide | Nervous system | 4 |
| Mesna | Detoxifying agent | Antioxidation? | Antidote for chemotherapy | 1984 | 1985 | 1991 | Germany | Anaphylaxis | 4 |
| Metamfetamine (desoxyephedrine) | Psychostimulant | TAAR1 receptor agonist | ADHD, obesity | 1944 | 1954 | 1973 | USA, Turkey, Oman, Nigeria | Drug abuse, drug dependence | 4 |
| Metamizole (dipyrone) ‡ | Analgesic | COX-1 & COX-2 inhibition | Analgesia | 1921 | 1952 | 1965 | 9 European, 11 Asian, and 5 African countries; Australia; USA; Mexico; Canada | Hematologic, immunologic (agranulocytosis) | 4 |
| Methapyrilene | Sedative-hypnotic | Histamine and cholinergic receptors' antagonist | Insomnia | 1947 | 1979 | 1979 | Germany, Dominican Republic, UK, Italy, Canada, Singapore, Hong Kong, Australia, Egypt, Panama, Brazil, Philippines, UAE, India, Oman, Chile, New Zealand, USA, Venezuela | Tumorigenicity | 5* |
| Methaqualone | Sedative-hypnotic | GABA receptor agonist | Hypnosedation | 1965 | 1966 | 1979 | Greece, Turkey, Oman, Zimbabwe, Pakistan, Ghana, UAE, India | Drug abuse | 4 |
| Methiodal sodium | Radiography |  | Urinary tract diagnostics | 1964 | 1972 | 1975 | Sweden | Muscle | 4 |
| Methylandrostenolone | Hormones | Protein synthesis | Tonic | 1960 | 1982 | 1982 | USA, France, UK, Germany, | Endocrine (masculinization) | 4 |
| Methylhexanamine  (DMAA) | Nasal decongestant | Norepinephrine & dopamine transporter blockade | Nasal decongestion | 1948 | 1950 | 1983 | Worldwide | Cardiovascular, nervous | 5* |
| Methylphenidate | Psychostimulant | Dopamine-norepinephrine reuptake inhibitor | ADHD | 1956 | 1961 | 1982 | Turkey, Oman, Nigeria | Drug abuse | 4 |
| Methylrosanilinium chloride | Antimicrobial |  | Bacterial & fungal infections | 1890 | 1970 | 1998 | Malaysia | Immunologic | 4 |
| Methyprylon | Sedative-hypnotic | GABA-A receptor modulation | Hypnosedation | 1955 | 1961 | 1984 | Zimbabwe | Drug abuse | 4 |
| Metipranolol | Antihypertensive | Non-selective beta blocker | Hypertension | 1986 | 1991 | 1991 | UK | Sensory systems (uveitis) | 4 |
| Metofoline | Analgesic | Opioid receptor agonist | Analgesia | 1962 | 1965 | 1965 | USA | Sensory systems | 5* |
| Metrizamide | Radiography |  | Contrast medium | 1977 | 1980 | 1992 | UK, Netherlands | Nervous system, sensory systems | 4 |
| Metrodin | Hormones | GnRH agonist | Invitro fertilization (IVF) | 1993 | 2003 | 2003 | UK | Nervous system | 4 |
| Mianserin | Antidepressant | Norepinephrine & serotonin reuptake inhibitor | Depression | 1975 | 1979 | 1988 | Oman | Hematologic (agranulocytosis) | 4 |
| Mibefradil | Antihypertensive | Calcium channel blockers | Hypertension | 1997 | 1998 | 1998 | USA; UK; Peru; South Africa; Jamaica; Bulgaria; Armenia | Drug interactions, musculoskeletal | 4 |
| Miglustat | Enzyme inhibitor | Glucocerebrosidase | Gaucher’s disease type 1 | 2002 | 2002 | 2002 | Israel | Unexplained cognitive dysfunction | 4 |
| Minaprine | Antidepressant | Reversible inhibitor of MAO-A | Depression | 1972 | 1983 | 1996 | France, Spain, Germany | Drug abuse, nervous system | 4 |
| Minocycline | Antimicrobial | Protein synthesis inhibitor | Bacterial infection | 1967 | 1974 | 1989 | Norway | Dizziness, vertigo | 4 |
| Molsidomine | Antianginal | Nitric oxide synthesis | Angina pectoris | 1972 | 1975 | 1985 | Germany | Tumorigenicity | 5* |
| Moxisylyte (thymoxamine; uroalpha) | Antianginal | Alpha1-adrenergic antagonist | Benign prostatic hyperplasia | 1989 | 1991 | 1993 | France | Liver | 4 |
| Mucopolysaccharide polysulfuric acid ester | Antiarthritic | Endogenous hyaluronate synthesis | Rheumatism | 1962 | 1987 | 1988 | Switzerland, France, Portugal, Austria | Skin: histochemical study | 4 |
| MMR vaccine (Urabe) | Vaccines | Antibody production | Mumps virus | 1983 | 1987 | 1988 | Worldwide | Nervous system, psychiatric | 2 |
| Muzolimine‡ | Antihypertensive | Diuresis | Hypertension | 1983 | 1987 | 1987 | Worldwide | Nervous system | 4 |
| Naftidrofuryl oxalate (IV) ‡ | Vasodilator | 5HT2 receptor antagonist | Intermittent claudication | 1974 | 1976 | 1992 | France; Germany, Spain | Cardiovascular, immunologic, liver, urinary tract | 4 |
| Nandrolone decanoate (injectable) | Hormones | Stimulate anabolism & inhibit catabolism | Hypogonadism | 1962 | 1989 | 1997 | France, Bangladesh | Drug abuse | 4 |
| Nandrolone phenylpropionate (injectable) | Hormones | Stimulate anabolism & inhibit catabolism | Hypogonadism | 1959 | 1972 | 1982 | Bangladesh | Drug abuse | 4 |
| Natalizumab | Monoclonal antibody | Inhibits α4 integrin | Multiple sclerosis & Crohn's disease | 2004 | 2005 | 2005 | USA | Nervous (combination) | 2 |
| Nebacumab‡ | Monoclonal antibody | Endotoxin-specific IgM | Sepsis | 1991 | 1993 | 1993 | Worldwide | Infection risk; accelerated deaths | 2 |
| Nefazodone‡ | Antidepressant | 5HT2 receptor antagonist | Depression | 1994 | 1999 | 2003 | European Union; Canada; Singapore | Hepatotoxicity | 4 |
| Neomycin sulfate (inj) | Antimicrobial | Protein synthesis inhibitor | Bacterial infection | 1949 | 1967 | 1983 | Bangladesh, Philippines, Nigeria, USA; Canada | Drug abuse, sensory systems, urinary tract | 4 |
| Nevirapine | Antimicrobial | Non-nucleoside reverse transcriptase inhibitor (NNRTI) | HIV | 1996 | 1997 | 2007 | Brazil | Liver | 4 |
| Nialamide | Antidepressant | MAOI | Depression | 1959 | 1969 | 1974 | Japan, India, Cuba, Denmark, Thailand, Venezuela, Canada | Drug interactions, liver | 4 |
| Nifedipine (10mg) | Antihypertensive | Calcium channel blockers | Hypertension | 1975 | 1989 | 1996 | Australia | Cardiovascular | 4 |
| Nifuroxazide (nifuroxazine) | Antimicrobial | Janus kinase inhibitor | Diarrhea and colitis | 1966 | 1997 | 2008 | Belgium | Immunologic, hematologic | 4 |
| Nikethamide | Respiratory stimulant | Unclear | Tranquilizer overdose | 1922 | 1953 | 1988 | Worldwide | Nervous system | 4 |
| Nimesulide‡ | Analgesic | COX-2 inhibitor | Analgesia | 1986 | 1998 | 1999 | Portugal, Israel, Spain, Bangladesh, Nigeria, India, Ireland, Ghana, Thailand, Vietnam | Liver | 4 |
| Niperotidine | Anti-ulcer | H2 receptor antagonist | Peptic ulcer | 1990 | 1995 | 1995 | Italy | Hepatotoxicity | 4 |
| Nitrefazole‡ | Anti-addiction | Inhibition of aldehyde dehydrogenase | Alcohol deterrent | 1982 | 1984 | 1984 | Germany, Austria | Liver | 4 |
| Nitrofurazone (Nitrofural) | Antimicrobial | Unclear | Bacterial infection | 1946 | 1974 | 1977 | Japan, USA, Armenia | Mutagenicity | 5* |
| Nitroxoline | Antimicrobial | ↓ in the biofilm density of *P. aeruginosa* | UTI | 1965 | 1966 | 1973 | Ireland, Thailand, Venezuela | Eyes | 5* |
| Nomifensine‡ | Antidepressant | Norepinephrine-dopamine reuptake inhibitor | Depression | 1976 | 1980 | 1986 | Worldwide | Hematologic, liver, respiratory | 4 |
| Norpseudoephedrine (Phenylpropanolamine) | Psychostimulant | Adrenoreceptor agonist, D1 receptor agonist | Nasal decongestion, obesity | 1947 | 1985 | 1987 | Germany, Brazil, Malaysia, Singapore, USA, Oman, Canada, Cuba, India, Cameroun, Nigeria, Portugal, Temor-Leste, South Korea | Hemorrhagic stroke | 4 |
| Noscapine | Antitussive | σ–receptor agonist | Cough | 1959 | 1984 | 1990 | Ireland, UK, Netherlands | Genotoxicity | 5* |
| Novobiocin (cathomycin) | Antimicrobial | Inhibitors of bacterial DNA gyrase | Staphylococcal infections | 1956 | 1957 | 1987 | Malaysia | Hematologic | 4 |
| Omeprazole (injectable) | Anti-ulcer | Proton pump inhibitor | Peptic ulcer | 1989 | 1994 | 1994 | Germany | Endocrine, genotoxicity, liver, musculoskeletal, nervous system, sensory systems | 4 |
| Opium in antitussives‡ | Antitussive | Opioid receptor agonist | Recreation | 1812 | 1842 | 1982 | Bangladesh, Italy | Drug dependence | 4 |
| Orciprenaline (metaprotenerol) | Bronchodilator | Beta2 adrenoceptor agonist | Asthma | 1961 | 1972 | 2009 | UK | Cardiovascular | 4 |
| Orgotein‡ | Antiarthritic | Free radical scavenging | Osteoarthritis | 1968 | 1987 | 1990 | Switzerland, Germany, Portugal | Immunologic | 4 |
| Oxeladin | Antitussive | Unclear | Cough | 1972 | 1991 | 1994 | Germany, France, Armenia, Canada | Carcinogenic | 5* |
| Oxolamine | Antitussive | Unclear | Cough | 1969 | 1972 | 1984 | Netherlands | Nervous system: hallucination in children | 4 |
| Oxomemazine‡ | Antitussive | Histamine and cholinergic receptors' antagonist | Cough | 1937 | 1979 | 1991 | European Union; USA | Respiratory depression in children (SIDS) | 4 |
| Oxyphenbutazone‡ | Analgesic | COX-1 & COX-2 inhibition | Analgesia | 1955 | 1974 | 1984 | Worldwide | Hematologic: Bone marrow suppression | 4 |
| Oxyphenisatin‡ | Laxative | ↑d mucosal tissue permeability | Constipation | 1925 | 1970 | 1972 | Worldwide | Liver | 4 |
| Pangamic acid | “Vitamin” | Transmethylation | Detoxification | 1950 | 1980 | 1984 | Greece | Mutagenic | 5* |
| Paramomycin | Antimicrobial | Protein synthesis inhibitor | Bacterial infection | 1959 | 1970 | 1989 | Spain | Renal damage, neuromuscular blockage and ototoxicity | 4 |
| Parecoxib | Analgesic | COX-2 inhibitor | Analgesia | 2002 | 2005 | 2005 | USA, European Union | Cardiovascular, respiratory | 2 |
| Pargyline | Antihypertensive | Monoamine oxidase inhibitor | Hypertension | 1963 | 1964 | 1979 | Germany | Interaction with tyramine | 4 |
| Pectin | Antidiarrheal | ↑d viscosity | Diarrhea | 1936 | 1974 | 1991 | Libya, India, Sri Lanka | Metabolic | 4 |
| Pemoline‡ | Psychostimulant | Dopamine agonist? | ADHD, narcolepsy | 1960 | 1973 | 2005 | Worldwide | Liver: hepatotoxicity | 4 |
| Pentobarbital‡ | Sedative-hypnotic | GABA-A receptor modulation | Hypnosedation | 1930 | 1953 | 1985 | Sweden | Drug overdose | 4 |
| Pentosan polysulfate sodium | Anti-thrombotic | Protective coating to the damaged bladder wall? | Cystitis, osteoarthritis | 1965 | 1985 | 1994 | France, USA | Hematologic: thrombocytopenia | 4 |
| Pentylenetetrazol | Circulatory & respiratory stimulant | Unclear | Convulsion | 1934 | 1954 | 1982 | USA | Convulsion | 4 |
| Pergolide Mesylate | Anti-parkinsonian | Dopamine receptor agonist | Parkinson's | 2002 | 2002 | 2007 | USA, Canada | Cardiovascular | 3 |
| Perhexiline lameate | Antianginal | Inhibits mitochondrial CPT1 | Angina pectoris | 1974 | 1974 | 1985 | UK, Spain, France | Hypoglycemia, liver, musculoskeletal, nervous system | 4 |
| Phenacetin | Analgesic | Metabolized to paracetamol | Analgesia | 1887 | 1948 | 1965 | 16 European, 14 Asian, 5 African, 4 S. American countries. New Zealand, USA, Canada | Hematologic, liver, tumorigenicity, urinary tract | 4 |
| Phenazone (antipyrine) | Analgesic | COX-1 & COX-2 inhibition | Analgesia | 1883 | 1958 | 1981 | UAE, Malaysia, Bahrain, Germany, Iraq | Hematologic | 4 |
| Phenazopyridine | Analgesic | Unclear | Urinary analgesia | 1927 | 1978 | 1984 | Greece | Carcinogenic | 5* |
| Phendimetrazine | Psychostimulant | Norepinephrine-dopamine releasing agent | Obesity | 1961 | 1979 | 1982 | Turkey | Drug abuse | 4 |
| Phenformin‡ | Hypoglycemic | ↓d gluconeogenesis | Diabetes | 1957 | 1963 | 1970 | 15 European & 6 Asian countries; Canada; New Zealand; Brazil; USA; Ethiopia | Metabolic acidosis | 4 |
| Phenicarbazide (phenylsemicarbazide) | Analgesic | Unclear | Analgesia | 1948 | 1950 | 1979 | Ireland | Serious adverse effects: hemolytic anemia | 4 |
| Phenmetrazine | Psychostimulant | Norepinephrine-dopamine releasing agent | Obesity | 1956 | 1959 | 1982 | Turkey, Oman, Nigeria | Drug abuse | 4 |
| Phenobarbital‡ | Sedative-hypnotic | GABA-A receptor modulation | Hypnosedation, convulsion | 1912 | 1929 | 1985 | Sweden, Mauritius, France | Abuse and intoxication; skin | 4 |
| Phenol (topical) | Antimicrobial | Absorption by bacteria | Bacterial infection | 1867 | 1973 | 1983 | Dominican Republic, Lithuania | Safer alternatives | 4 |
| Phenolphthalein | Laxative | Direct action on intestinal muscles | Constipation | 1902 | 1953 | 1979 | Europe, Yemen, Bangladesh, Canada, France, Morocco, Oman, Japan, Saudi Arabia, Bahrain | Skin, metabolic, muscular | 4 |
| Phenoxypropazine | Antidepressant | MAOI | Depression | 1961 | 1964 | 1966 | UK | Drug interactions, liver | 4 |
| Phentermine | Psychostimulant | TAAR1 agonist | Obesity | 1959 | 1964 | 1981 | Sweden, UAE, Mauritius, Turkey, Oman, UK, Venezuela | Drug abuse | 4 |
| Phentolamine mesilate | Antihypertensive | Alpha-adrenergic antagonist | Erectile dysfunction | 1998 | 1998 | 2000 | Singapore | Carcinogenicity | 5* |
| Phenylbutazone‡ | Analgesic | COX-1 & COX-2 inhibition | Analgesia | 1949 | 1953 | 1985 | 6 European, 6 Asian & 3 African countries; Chile; Panama | Hematologic, liver, urinary tract | 4 |
| Phenylephrine | Decongestant | α1-adrenergic receptor agonist | Mydriasis | 1949 | 1968 | 1987 | UK | Eyes | 4 |
| Phthalylsulfathiazole | Antimicrobial | Inhibits dihydropteroate synthetase | Bacterial infection | 1946 | 1950 | 1982 | Bangladesh | Granulocytopenia | 4 |
| Pifoxime | Analgesic | COX-1 & COX-2 inhibition | Analgesia | 1975 | 1975 | 1976 | France | Nervous system, psychiatric | 4 |
| Pioglitazone | Hypoglycemic | PPARs activation | Diabetes | 1999 | 2011 | 2011 | Europe | Risk of bladder CA | 3 |
| Pipamazine | Emetic | Phenothiazine analogue | Emesis | 1959 | 1960 | 1969 | USA | Lack of efficacy | 5 |
| Pipenzolate | Anti-ulcer | Muscarinic receptor antagonist | Peptic ulcer | 1960 | 1990 | 1990 | Pakistan | Overdose potential | 4 |
| Piperazine | Antihelminth | GABA receptor inhibition | Worm infestation | 1949 | 1957 | 1983 | Malaysia, Armenia | Immunologic, nervous system, tumorigenicity | 4 |
| Pipradrol | Psychostimulant | Norepinephrine-dopamine reuptake inhibitor | Obesity, narcolepsy, ADHD | 1953 | 1968 | 1982 | USA, Turkey, Denmark, Venezuela | Nervous, cardiovascular | 4 |
| Pirprofen‡ | Analgesic | COX-1 & COX-2 inhibition | Analgesia | 1982 | 1986 | 1990 | Worldwide | Gastrointestinal, liver, urinary tract | 4 |
| Pituitary chorionic gonadotropin (injectable) | Hormones | Ovulation trigger | Infertility | 1930 | 1958 | 1972 | USA | Immunologic | 4 |
| Podophyllum resin | Laxative | Mitotic spindle poison | Constipation | 1844 | 1962 | 1970 | Italy, France, Egypt | Teratogenicity | 4 |
| Polidexide | Antilipemic | Anion exchange | Hyperlipidemia | 1974 | 1977 | 1977 | UK | Oculomucocutaneous syndrome | 4 |
| Polyoxyethylated castor oil | Adjuvant |  | Emulsifier | 1970 | 1973 | 1984 | Worldwide | Anaphylaxis, hyperlipidemia | 4 |
| Polyvinylpyrrolidone (Polyvidone; povidone) | Adjuvant | Permeability through circulatory system | Suspending and dispersing of IV drugs | 1957 | 1967 | 1983 | Germany, Pakistan, Egypt, USA | Metabolic: granulomatous lesions | 4 |
| Potassium arsenate | Tonic | Degradation of the aberrant retinoic acid receptor α fusion protein | Leukemia; Psoriasis | 1878 | 1968 | 1980 | USA | Tumorigenicity | 4 |
| Potassium canrenoate | Antihypertensive | Aldosterone antagonist | Hypertension, ascites | 1968 | 1985 | 1986 | Germany | Carcinogenic | 5* |
| Potassium chloride | Electrolyte | Replenishment of potassium stores | Hypokalemia | 1962 | 1964 | 1989 | Belgium, France, USA, Canada | Gastrointestinal perforation | 4 |
| Potassium nitrate | Antihypertensive | Nitric oxide synthesis | Hypertension | 1901 | 1975 | 1981 | France, Egypt, Venezuela | Tumorigenicity | 3 |
| Practolol‡ | Antihypertensive | Beta-blocker | Hypertension | 1970 | 1972 | 1975 | Greece, Turkey, New Zealand, Denmark, Thailand, Singapore, UK, Mauritius, India, Germany, Norway, Venezuela | Gastrointestinal, sensory systems, skin | 4 |
| Pramipexole dihydrochloride monohydrate | Antiparkinsonian | Dopamine receptor agonist | Parkinson's | 1997 | 1999 | 2006 | Europe | Sudden onset of sleep | 3 |
| Prenylamine‡ | Antianginal | Calcium channel blocker | Angina pectoris | 1960 | 1973 | 1989 | Worldwide | Cardiovascular: multifocal ventricular tachycardia | 4 |
| Probucol | Antioxidant | Unclear | Hyperlipidemia | 1980 | 1989 | 1989 | Germany, France, USA | Cardiovascular: Torsade de pointes | 4 |
| Proglumide | Anti-ulcer | Cholecystokinin antagonist | Peptic ulcer | 1970 | 1984 | 1989 | Japan, Germany | Respiratory | 3 |
| Pronethalol | Antihypertensive | Beta-blockade | Angina pectoris | 1963 | 1963 | 1965 | UK | Tumorigenicity | 5* |
| Propanidid | Anesthetic | Unclear | General anesthesia | 1963 | 1965 | 1983 | UK, Norway, France | Immunologic reactions | 4 |
| Propofol (children) ‡ | Anesthetic | GABA-A receptor agonist, sodium channel blockade | Anesthesia | 1987 | 1992 | 1992 | Israel, Norway, UK, Lithuania | Metabolic, hepatic, nervous; deaths | 4 |
| Propyphenazone‡ | Analgesic | COX-1 & COX-2 inhibition | Analgesia | 1951 | 1980 | 1989 | Turkey, UAE, Bahrain, Ireland | Hematologic, Immunologic: Lyell's syndrome | 4 |
| Proxibarbal | Sedative-hypnotic | GABA-A receptor modulation | Hypnosedation | 1956 | 1990 | 1998 | France, Italy, Spain, Portugal and Turkey | Hematologic, immunologic | 4 |
| Pseudoephedrine | Sympathomimetic | Direct action on adrenergic receptors | Nasal decongestion | 1959 | 1963 | 2008 | Iraq, Colombia, Thailand | Nervous, GIT, immunologic, cardiovascular, teratogenic | 3 |
| Pumactant‡ | Surfactant | Lowers lung surface tension | Respiratory distress | 1992 | 2000 | 2000 | UK | “Accelerated deaths” | 2 |
| Purified hexavalent vaccine (Hexavac) | Vaccines | Immunogenicity | Bacterial and viral infections | 2000 | 2005 | 2005 | Europe | Decreased immunogenicity | 2 |
| Pyrithyldione/diphenhydramine | Sedative-hypnotic | Similar to barbiturates | Insomnia | 1940 | 1949 | 1997 | Spain | Hematologic: agranulocytosis | 4 |
| Pyritinol | Nootropic | Increased cerebrovascular blood flow | Dementia syndromes | 1961 | 1973 | 1982 | Bangladesh | Insufficient therapeutic value and risk of misuse | 4 |
| Pyrovalerone | Psychostimulant | Norepinephrine-dopamine reuptake inhibitor | Obesity, chronic fatigue syndrome | 1974 | 1975 | 1979 | France | Drug abuse | 4 |
| Pyrrolizidine | Antitussive | Coating effect | Cough, sinusitis | 1967 | 1968 | 1992 | Germany, Belgium, UK | Liver | 5* |
| Rapacuronium bromide‡ | Anesthetic | Non-depolarizing neuromuscular blocker | Anesthesia | 1999 | 1999 | 2001 | USA | Respiratory: Bronchospasm | 3 |
| Remoxipride | Antipsychotic | D2 receptor antagonist | Psychoses, schizophrenia | 1991 | 1993 | 1994 | Worldwide | Hematologic | 4 |
| Rimonabant | Psychostimulant | Cannabinoid receptor agonist | Obesity | 2006 | 2006 | 2007 | Europe, India | Psychiatric | 1 |
| Rofecoxib‡ | Analgesic | COX-2 inhibitor | Analgesia, osteoarthritis | 1999 | 2002 | 2004 | Worldwide | Cardiotoxicity | 1 |
| Rosiglitazone‡ | Hypoglycemic | ↓d gluconeogenesis | Diabetes | 1999 | 2007 | 2011 | UK; New Zealand; South Africa, India | Cardiovascular | 1 |
| Rotavirus vaccine | Vaccines | Immunogenicity | Rotavirus | 1998 | 1999 | 1999 | USA, UK | Gastrointestinal: intussusception | 3 |
| Santonin‡ | Antihelminth | Paralysis of nerve impulses | Ascariasis | 1911 | 1933 | 1978 | Singapore | Neurotoxicity | 4 |
| Sargramostim | Immunostimulator | Granulocyte macrophage colony-stimulating factor (GM-CSF) | Bone marrow transplantation | 1993 | 1999 | 2008 | USA | Immune-mediated | 4 |
| Secobarbital/ quinalbarbital | Sedative-hypnotic | GABA-A receptor modulation | Hypnosedation, convulsion | 1934 | 1958 | 1990 | France, Norway, Netherlands, Ghana, New Zealand, Oman | Drug abuse | 4 |
| Selegiline | Antiparkinsonian | MAO-B inhibitor | Parkinson's, depression, dementia | 1982 | 1991 | 1997 | USA | Drug interaction | 4 |
| Sertindole‡ | Antipsychotic | 5HT & D2 receptor antagonist | Psychoses | 1996 | 1996 | 1998 | UK, Bulgaria, Spain | Cardiovascular, death | 2 |
| Sibutramine‡ | Psychostimulant | Serotonin-norepinephrine reuptake inhibitor | Obesity | 2001 | 2002 | 2002 | European Union; 4 Asian countries; Australia; Canada; Mexico; New Zealand; USA | Cardiovascular | 4 |
| Sitaxentan sodium‡ | Antihypertensive | Endothelin receptor antagonist | Pulmonary arterial hypertension | 2006 | 2009 | 2010 | Worldwide | Hepatotoxicity | 4 |
| Sodium dibunate (ethyl dibunate) | Antitussive | Unclear | Cough | 1963 | 1968 | 1982 | Philippines | Nervous | 5* |
| Sodium hydrogen bicarbonate (pediatric) | Antacid | Counteracts acidity | Indigestion | 1966 | 1983 | 1997 | Nepal | Safety concerns: metabolic | 4 |
| Somatropin (growth hormone) ‡ | Hormones | Binds to the human growth hormone receptor | Hypopituitary dwarfism | 1973 | 1985 | 1985 | Europe; Egypt; New Zealand; USA; Oman; Thailand | Infection risk: Creutzfeldt-Jakob disease | 4 |
| Soruvidine‡ | Antimicrobial | Inhibits DNA polymerase | Herpes viral infection | 1993 | 1993 | 1993 | Germany; Japan | Drug interactions with 5-fluorouracil | 4 |
| Sparfloxacin | Antimicrobial | Inhibits DNA gyrase | Bacterial infection | 1993 | 1995 | 1995 | Europe, USA | QTc prolongation, phototoxicity | 4 |
| Sparteine sulphate | Hormone analogue | Prostaglandin stimulation? | Labour | 1939 | 1963 | 1979 | USA | Uterine tetany | 4 |
| Strychnine and salts | Aphrodisiac | Acetylcholine receptor antagonist | Impotence | 1753 | 1882 | 1979 | Canada, Brazil, Bangladesh, Japan, Pakistan, UAE, Philippines | Nervous | 4 |
| Sulfacarbamide (Sulfanilylurea) | Antimicrobial | Inhibits dihydropteroate synthetase | Bacterial infection | 1946 | 1959 | 1992 | Germany | Hematologic, liver, skin, urinary tract | 4 |
| Sulfadicramide (sulfacetamide) | Antimicrobial | Inhibits dihydropteroate synthetase | Bacterial infection | 1942 | 1976 | 1992 | Germany | Negative benefit-to-harm balance: eye, skin, immune | 4 |
| Sulfadimethoxine‡ | Antimicrobial | Inhibits dihydropteroate synthetase | Bacterial infection | 1958 | 1961 | 1966 | USA | Skin | 4 |
| Sulfadimidine | Antimicrobial | Inhibits dihydropteroate synthetase | Bacterial infection | 1942 | 1954 | 1992 | USA; Germany; Armenia | Hematologic: thrombocytopenia | 4 |
| Sulfaguanidine | Antimicrobial | Inhibits dihydropteroate synthetase | Bacterial infection | 1941 | 1969 | 1971 | Dominican Republic, Iran, Turkey, Pakistan, Nepal, Germany, UAE, Denmark, Venezuela | Hematologic: agranulocytosis | 4 |
| Sulfamerazine sodium | Antimicrobial | Inhibits dihydropteroate synthetase | Bacterial infection | 1943 | 1955 | 1992 | Germany | Hematologic; skin | 4 |
| Sulfamethizole | Antimicrobial | Inhibits dihydropteroate synthetase | Bacterial infection | 1953 | 1963 | 1984 | Sweden | Immunologic | 4 |
| Sulfamethoxydiazine | Antimicrobial | Inhibits dihydropteroate synthetase | Bacterial infection | 1962 | 1968 | 1988 | Germany | Skin | 4 |
| Sulfamethoxypyridazine | Antimicrobial | Inhibits dihydropteroate synthetase | Bacterial infection | 1957 | 1958 | 1984 | Sweden, Pakistan, UAE, UK, Canada, Argentina, France | Hematologic, skin | 4 |
| Sulfanilamide | Antimicrobial | Inhibits dihydropteroate synthetase | Bacterial infection | 1936 | 1957 | 1992 | Germany | Sensory, Immunologic | 4 |
| Sulfathiazole | Antimicrobial | Inhibits dihydropteroate synthetase | Bacterial infection | 1941 | 1954 | 1970 | USA, Philippines, Dominican Republic, France, UAE, India | Hematologic, liver, skin, urinary tract | 4 |
| Sulfisomidine | Antimicrobial | Inhibits dihydropteroate synthetase | Bacterial infection | 1952 | 1972 | 1992 | Germany | Negative benefit-to-harm balance | 4 |
| Sulfonamides (topical) | Antimicrobial | Inhibits dihydropteroate synthetase | Bacterial infection | 1930 | 1957 | 1986 | Chile | Immune-mediated | 4 |
| Suloctidil‡ | Vasodilator | Calcium channel blockade, inhibits thromboxane B2 | Intermittent claudication | 1975 | 1983 | 1985 | Worldwide | Liver: hepatotoxicity | 4 |
| Suprifen with tussilax | Antitussive |  | Cough | 1971 | 1971 | 1972 | France, Germany | Liver: hepatotoxicity | 3 |
| Suprofen | Analgesic | COX-1 & COX-2 inhibition | Analgesia | 1983 | 1986 | 1986 | Worldwide | Urinary tract | 4 |
| Suxibuzone | Analgesic | COX-1 & COX-2 inhibition | Analgesia | 1974 | 1982 | 1986 | Oman, Italy | Carcinogenesis | 5* |
| Technetium (^99m^Tc) fanolesomab‡ | Radiography | Radioisotope | Radiographic imaging | 2004 | 2005 | 2005 | USA | Cardiovascular | 4 |
| Tegaserod | Antispasmodic | 5-HT4 receptor agonist | Irritable bowel syndrome, constipation | 2002 | 2004 | 2007 | USA, Canada, India, Jordan, Australia, Switzerland, China, Argentina | Cardiovascular | 1 |
| Temafloxacin‡ | Antimicrobial | Inhibits DNA gyrase | Bacterial infection | 1991 | 1992 | 1992 | Worldwide | Hematologic, immunologic, liver, metabolism, urinary tract | 4 |
| Temazepam (gel capsules) ‡ | Sedative-hypnotic | GABA-A receptor binding | Hypnosedation | 1969 | 1981 | 1999 | Australia | Drug abuse | 3 |
| Terconazole | Antimicrobial | Disrupts cell membrane permeability by cytochrome P450 14-alpha-demethylase | Fungal infection | 1980 | 1988 | 1991 | Sweden, Germany | Immunologic, skin | 4 |
| Terfenadine‡ | Antihistamine | H1-receptor antagonist | Allergy | 1985 | 1985 | 1997 | 3 European, 2 African, 3 Asian & 3 South American countries; USA, Canada | Cardiovascular, immunologic, liver, skin | 4 |
| Terodiline‡ | Antispasmodic | Calcium channel blockade, blocks cholinergic receptor | Urinary incontinence | 1965 | 1989 | 1991 | Worldwide | Cardiovascular | 4 |
| Testosterone propionate (injectable) | Hormones | Androgen-receptor modulation | Hypogonadism, sexual dysfunction | 1937 | 1952 | 1982 | Bangladesh | Drug abuse | 4 |
| Tetrabamate | Anxiolytic | Febarbamate, difebarbamate plus phenobarbital | Alcohol dependence | 1981 | 1992 | 2001 | France, Spain | Liver, skin | 4 |
| Tetrachlorosalicylanilide | Antimicrobial | Leakage of cell contents | Bacterial infection | 1949 | 1961 | 1975 | USA, Japan | Skin | 4 |
| Tetracycline (pediatric) | Antimicrobial | Protein synthesis inhibitor | Bacterial infection | 1948 | 1959 | 1975 | 8 Asian, 2 African, 2 Australasian countries; USA, Italy | Teeth; bone | 3 |
| Tetrazepam | Sedative-hypnotic | Benzodiazepine site agonist | Hypnosedation | 1960 | 1988 | 2013 | EU | Dermatitis | 4 |
| Thalidomide | Immunomodulator | ? CNS mechanism | Hyperemesis | 1957 | 1961 | 1961 | Belgium, Finland, Indonesia, Canada, Brazil, Denmark, India, Singapore, Venezuela, UK, Germany | Nervous system, teratogenicity | 4 |
| Thenalidine tartrate‡ | Antihistamine | H1-receptor blocker | Allergy | 1953 | 1958 | 1958 | USA; UK; Sweden; France; Cyprus; Australia; Finland; Norway, Canada | Hematologic: neutropenia | 4 |
| Thiobutabarbital | Sedative-hypnotic | GABA-A receptor modulation | Hypnosedation | 1939 | 1981 | 1993 | Germany | Urinary tract | 5* |
| Thioridazine | Antipsychotic | 5HT2 receptor antagonist | Psychoses | 1959 | 1961 | 2000 | Worldwide | Retinopathy, cardiovascular | 4 |
| Thorium dioxide | Radiography | Radiocontrast | Diagnostic imaging | 1928 | 1931 | 1955 | UK | Tumorigenicity | 4 |
| Tick-borne encephalitis vaccine | Vaccines | Immunogenicity | Viral infection | 1999 | 1999 | 2000 | Germany | Hyperpyrexia | 2 |
| Tienilic acid (ticrynafen) ‡ | Antihypertensive | Loop diuretic | Hypertension, kidney stones | 1976 | 1979 | 1980 | Greece; Philippines; USA; Brazil; Germany; Panama; France; India; Venezuela | Liver, urinary tract | 4 |
| Tilbroquinol | Antiprotozoal | ? | Diarrhea (amoebiasis) | 1969 | 1996 | 1997 | France, Morocco, Saudi Arabia, Switzerland | Liver, nervous system | 4 |
| Timonacic acid (thioproline) | Antioxidant | Cysteine release and restoration of glutathione concentrations | Hepatitis | 1975 | 1981 | 1982 | France | Drug overdose (toxicity) | 3 |
| Tocainide‡ | Antiarrhythmic | Sodium channel blockade | Cardiac arrhythmia | 1981 | 1986 | 1986 | USA | Hematologic: agranulocytosis, aplastic anemia | 4 |
| Tolcapone‡ | Antiparkinsonian | COMT inhibitor | Parkinson's | 1998 | 1998 | 1998 | UK; Australia; Ireland; Spain; Portugal; Lithuania; Bulgaria; Canada | Liver | 4 |
| Tolrestat‡ | Hypoglycemic | Aldase reductase inhibitor | Diabetes | 1982 | 1995 | 1996 | Worldwide | Liver | 2 |
| Tranylcypromine | Antidepressant | MAOI | Depression | 1961 | 1963 | 1964 | Italy, Belgium, Venezuela, Canada | Cardiovascular, drug interactions | 4 |
| Trazodone | Antidepressant | Binds at 5-HT2 receptor | Depression | 1973 | 1983 | 1985 | Norway | Carcinogenic | 5* |
| Tretinoin | Retinoid | Modulates cell-cycle progression, cellular differentiation, cell survival and apoptosis | Acne | 1973 | 1977 | 1988 | Germany | Carcinogenic | 5* |
| Triacetyldiphenolisatin | Laxative | ↑d mucosal tissue permeability | Constipation | 1961 | 1967 | 1971 | Australia, Germany, Italy, Canada, Cyprus, New Zealand, Venezuela | Photosensitivity; hepatotoxicity | 4 |
| Triazolam | Sedative-hypnotic | GABA-A receptor binding | Hypnosedation | 1978 | 1978 | 1979 | Mauritius, Europe, Brazil, Oman | Nervous system, psychiatric | 4 |
| Trimethobenzamide (suppository) | Antihistamine | ?Chemoreceptor trigger zone | Motion sickness, GI infections, medication-induced nausea | 1959 | 1976 | 2007 | USA | Due to new FDA regulation; lack of effectiveness. Teratogenicity | 3 |
| Triparanol | Antilipemic | δ 24-reductase inhibitor | Hyperlipidemia | 1959 | 1961 | 1962 | USA, France | Sensory systems, skin | 4 |
| Troglitazone‡ | Hypoglycemic | PPARs activation | Diabetes | 1997 | 1997 | 1997 | UK; Switzerland; Peru; Jamaica; Chile, USA, Canada | Liver | 4 |
| Trovafloxacin‡ | Antimicrobial | Inhibits DNA gyrase | Bacterial infection | 1997 | 1997 | 1999 | European Union; Philippines; Syria; Vietnam; Singapore, Canada, USA | Liver | 4 |
| Urethane | Solvent | ?Interferes with pyrimidine metabolism | Cancer (CML) | 1933 | 1943 | 1963 | Brazil, Cuba, Denmark, Egypt, Japan, Thailand, USA, Canada, Italy, Greece, Denmark, Venezuela, France | Hematologic, liver, tumorigenicity | 5* |
| Valdecoxib | Analgesic | COX-2 inhibitor | Analgesia | 2001 | 2003 | 2005 | USA, Canada, Europe, India | Cardiovascular; skin | 2 |
| Veralipride | Neuroleptic | Selective antagonism of hypothalamic D2 receptors | Climacteric | 1979 | 1982 | 2007 | Europe, Brazil (reference to EMA) | Psychiatric | 4 |
| Vigabatrin (gamma-vinyl-GABA) | Antiepileptic | Inhibits GABA transaminase (GABA-T) | Hypnosedation | 1989 | 1989 | 1991 | Norway | Not medically justified; neurotoxicity | 5* |
| Vinbarbital‡ | Sedative-hypnotic | GABA-A receptor modulation | Hypnosedation | 1939 | 1975 | 1984 | Sweden | Drug abuse | 4 |
| Vincamine | Nootropic | Unclear | Meniere's disease, vertigo | 1955 | 1974 | 1980 | Hungary, Germany | Cardiovascular, hematologic | 4 |
| Vitamin B complex (injectable) | Hematinic | Co-factor for biochemical transformation | Anemia | 1949 | 1953 | 2008 | Iraq | Immunologic | 4 |
| Vitamin E (injectable) ‡ | Antioxidant | Downregulation of ICAM-1 and VCAM-1 | Retinal deterioration in neonates | 1983 | 1984 | 1984 | USA | Death, hematologic, liver, urinary tract | 3 |
| Xenazoic acid (xenalamine) | Antimicrobial | ? Inhibits intracellular stages viral biological cycle | Viral infection | 1960 | 1965 | 1965 | Belgium, France, Venezuela | Liver | 4 |
| Ximelagatran | Anticoagulant | Direct thrombin inhibitor | DVT, stroke | 2005 | 2006 | 2006 | Europe, Brazil | Liver | 2 |
| Zimeldine | Antidepressant | 5 HT reuptake inhibitor | Depression | 1982 | 1982 | 1983 | Worldwide | Liver, nervous system | 4 |
| Zipeprol HCl‡ | Antitussive | Laryngeal nerve stimulation; excitation of tracheo-bronchial receptors | Cough | 1971 | 1984 | 1991 | Philippines, Brazil, Spain, Switzerland, Turkey | Drug abuse, drug dependence, nervous system | 4 & 5 |
| Zomepirac‡ | Analgesic | COX-1 & COX-2 inhibition | Analgesia | 1979 | 1981 | 1983 | Worldwide | Immunologic, urinary tract | 4 |
| Zopiclone | Sedative-hypnotic | GABA-A receptor binding | Hypnosedation | 1985 | 1985 | 1986 | Israel, Norway | Carcinogenicity | 5* |

**ABBREVIATIONS:** ADHD: Attention deficit hyperactivity disorder; COMT: Catechol-O-methyl transferase; COX-1: Cyclooxygenase 1; COX-2: Cyclooxygenase 2; CPT1: Carnitine palmitoyltransferase I; CTZ: Chemoreceptor trigger zone; DNA: Deoxyribonucleic acid; D1: Dopamine 1; D2: Dopamine 2; D3: Dopamine 3; DVT: Deep venous thrombosis; GABA: Gamma-aminobutyric acid; HMG CoA: 3-hydroxy-3-methyl-glutaryl-CoA; hURAT1: Human uric acid transporter 1; H1: Histamine 1; ICAM-1: Intercellular Adhesion Molecule 1; MAOI: Monoamine oxidase inhibitor; NMDA: N-methyl-D-aspartate; PPAR: Peroxisome proliferator-activated receptors; SSRI: Selective serotonin reuptake inhibitor; TAAR 1: Trace amine-associated receptor 1; VCAM-1: Vascular cell adhesion protein 1; 5 HT: 5-hydroxytryptamine

^∆^Re-prohibited again in 1986 following FDA legal action (<http://www.atsdr.cdc.gov/toxprofiles/tp64.pdf>)

†Based on the Oxford Centre for Evidence-Based Medicine Levels of Evidence [13]. Level 1: Systematic review of randomized trials, systematic review of nested case-control studies, Level 2: Individual randomized trial or (exceptionally) observational study with dramatic effect; Level 3: Non-randomized controlled cohort/follow-up study (post-marketing surveillance); Level 4: Case-series, case-control, or historically controlled studies; Level 5: Mechanism-based reasoning

*First withdrawal based on evidence from animal research

‡Products were withdrawn in association with deaths

§The first reported adverse reaction appeared after first withdrawal from the market
